# The ER–Golgi intermediate compartment: a central hub integrating membrane trafficking and stress responses

**DOI:** 10.1038/s44319-026-00908-z

**Published:** 2026-08-22

**Authors:** Yingying Guo, Jianfei Zheng, Lei Liu, Shulin Li, Min Zhang, Liang Ge

**Affiliations:** 1 State Key Laboratory of Membrane Biology, Tsinghua-Peking Center for Life Sciences, School of Life Sciences, Tsinghua University, Beijing, China; 2 Department of Cell Biology and Genetics, School of Basic Medical Sciences, Xi’an Jiaotong University, Xi’an, China; 3 State Key Laboratory of Membrane Biology, Tsinghua-Peking Center for Life Sciences, School of Pharmaceutical Sciences, Tsinghua University, Beijing, China

**Keywords:** Membranes & Trafficking, Organelles

## Abstract

The endoplasmic reticulum–Golgi intermediate compartment (ERGIC) is a dynamic membrane system at the ER–Golgi interface, traditionally viewed as a transient station for COPII- and COPI-dependent trafficking. Emerging evidence redefines the ERGIC as a stress-responsive regulatory hub that integrates membrane trafficking with cellular adaptation. In addition to coordinating bidirectional transport and cargo sorting, the ERGIC actively participates in protein quality control during ER stress and remodels trafficking flux under perturbations. It serves as a platform linking secretory pathways to stress signaling, contributing to autophagosome biogenesis, facilitating unconventional protein secretion under stress conditions, and modulating innate immune responses, including STING activation. The ERGIC is also co-opted by pathogens such as coronaviruses, underscoring its role at the interface of membrane remodeling and host defense. These functions position the ERGIC as a central integrator of trafficking dynamics and stress responses, whose structural plasticity enables rapid adaptation to physiological and pathological challenges.

## Abstract (web summary)

This review provides a comprehensive discussion of the ERGIC, which emerges as a central hub integrating membrane trafficking and the cellular response to stress. It participates in protein quality control, autophagosome biogenesis, unconventional secretion and STING-dependent innate immune responses.

## Graphical abstract

**Figure:**
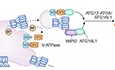

## Introduction

In the 1950s, pioneering work by Palade, Claude, and colleagues revolutionized cell biology through the optimization of electron microscopy fixation and staining techniques, coupled with the development of robust biochemical approaches (Claude, 1946; Claude and Fullam, 1946; Palade, 1955, 1956; Palade and Porter, 1954; Porter et al, 1945). These advances collectively led to the discovery of the secretory pathway, highlighting the central roles of the endoplasmic reticulum (ER) and the Golgi complex (Jamieson and Palade, 1967; Palade and Claude, 1949). Subsequent seminal studies by Schekman, Rothman, and Südhof marked a transformative breakthrough in the field of vesicular trafficking, identifying vesicular carriers and, in particular, characterizing the coat protein (COP)-coated vesicles as the principal mediators of ER-to-Golgi transport (Barlowe et al, 1994; Hata et al, 1993; Novick et al, 1980; Novick and Schekman, 1979; Waters et al, 1991; Zhang et al, 1994). Together, these discoveries established the foundation of modern membrane trafficking research and shaped the current understanding of the endomembrane system—concepts now central to every cell biology textbook.

While the ER–Golgi axis is widely regarded as the core conduit of secretory membrane trafficking, early ultrastructural studies also noted the presence of tubular-vesicular compartments adjacent to the cis-Golgi (Budnik and Stephens, 2009; Saraste and Kuismanen, 1984). This organelle, later termed the ER–Golgi intermediate compartment (ERGIC), has been extensively studied with respect to its morphology and spatial organization (Klumperman et al, 1998; Saraste and Svensson, 1991; Schweizer et al, 1988; Schweizer et al, 1991; Sesso et al, 1994). However, its precise functional role remained elusive for decades. More recently, with increasing attention to processes beyond canonical ER–Golgi transport, the ERGIC has emerged as a multifunctional platform implicated in diverse cellular events (Appenzeller-Herzog and Hauri, 2006; Saraste and Marie, 2018; Saraste and Prydz, 2026).

This review aims to synthesize and discuss recent advances in our understanding of the ERGIC, with a focus on its discovery, structural organization, functional diversity, and regulatory mechanisms.

## Early observations of a tubular and fenestrated juxta-Golgi structure

In the 1950s, the advent of electron microscopy enabled clear visualization of Golgi apparatus stacks, first in animal cells and later in plant cells (Dalton and Felix, 1954; Mollenhauer, 1959). While the Golgi apparatus in animal cells is typically concentrated in the perinuclear region, in plant cells it is more dispersed; nevertheless, in both systems the organelle appeared as stacked lamellae (Brandizzi and Barlowe, 2013). This led to the prevailing assumption that the Golgi structures in animal and plant cells were fundamentally equivalent.

A comprehensive ultrastructural analysis of *Anthoceros* meristems, published in 1960 by Irene Manton, confirmed the presence of Golgi stacks resembling those previously described, but also revealed a distinct subpopulation of unusual Golgi-related structures characterized by clusters of projecting tubules and fenestrated morphologies (Manton, 1960). These observations were later supported and extended by Hilton Mollenhauer and James Morré (Cunningham et al, 1966; Mollenhauer and Morre, 1966). Similar structures were subsequently identified in animal cells, first described in 1969 by Charles Flickinger in epididymal tissue (Flickinger, 1969) and later in mitotic cells and specialized secretory contexts, including protein and lipoprotein transport in nerve and liver cells (Claude, 1970; Maul and Brinkley, 1970; Zeligs and Wollman, 1979). Although functional studies were lacking, these early ultrastructural investigations suggested a close association, and possibly continuity, between the fenestrated tubular network and the cis-Golgi (Ladinsky et al, 1999; Mollenhauer and Morre, 1998) and implied a potential role in membrane trafficking.

## Establishment of the concept of the ER–Golgi intermediate compartment

Studies on viral protein trafficking laid the groundwork for the concept of a functionally distinct, intermediate compartment between the ER and the Golgi. In 1984, Jaakko Saraste and colleagues developed a temperature-shift assay in which membrane proteins of Semliki Forest virus (SFV) were successfully trapped in a pre-Golgi vacuolar element at a reduced temperature (15 °C) (Saraste and Kuismanen, 1984). In parallel, John Tooze, Sharon Tooze, and colleagues reported that murine hepatitis virus (MHV) particles were first observed budding within a tubular-vesicular structure located between the ER and the Golgi apparatus (Tooze et al, 1984). Their follow-up studies demonstrated that the tubular-vesicular compartment could modify the MHV glycoprotein E1 through N-acetylgalactosamine(GalNAc) addition, and importantly, that this tubular-vesicular compartment existed even in uninfected cells (Tooze et al, 1988). Collectively, these pioneering works—leveraging viral infection models and trafficking manipulation assays—enabled the detection of a transition compartment/pre-Golgi between the ER and cis-Golgi, and underscored its critical role in viral protein trafficking. Furthermore, they provided a clue that the transition compartment may be compositionally distinct from both the ER and Golgi, as reflected by its ability to support GalNAc modifications (Saraste et al, 1986; Tooze et al, 1984).

Subsequently, in 1987, Saraste and colleagues generated a polyclonal antibody against the cis-Golgi of rat pancreas, which recognized a 58 kDa protein (P58). Immunoelectron microscopy revealed that P58 localized to the fenestrated cis-most cisterna, likely a pre-Golgi region (Saraste et al, 1987). Shortly thereafter, in 1988, Hans-Peter Hauri’s group identified a monoclonal antibody (G1/93) during their development of Golgi markers. Interestingly, instead of recognizing bona fide Golgi proteins, this antibody detected a 53 kDa membrane protein (P53). Both fluorescence and EM studies showed that P53 was highly enriched in tubular-vesicular elements in close proximity to the cis-Golgi, suggesting the existence of a new compartment in the biosynthetic transport pathway between the ER and Golgi (Schweizer et al, 1988). Subsequent studies revealed that rat P58 and human P53 share 89% sequence identity, demonstrating that they represent homologous proteins (Lahtinen et al, 1996).

With these markers at hand, further functional assays using membrane trafficking models provided strong evidence for the role of these structures in protein transport. Temperature-sensitive mutants of vesicular stomatitis virus G protein (tsO45-VSV-G) (Hauri’s group) or the temperature-sensitive E1 glycoprotein of Semliki Forest virus (SFV ts-1) (Saraste and colleagues) were found to accumulate predominantly in P53- and P58-positive membranes, respectively (Saraste and Svensson, 1991; Schweizer et al, 1990).

Biochemical studies confirmed that these membranes possessed a protein composition distinct from both the ER and Golgi, demonstrating the existence of an independent compartment (Schweizer et al, 1991). Almost in parallel, Lippincott-Schwartz and colleagues identified a Brefeldin A (BFA) insensitive cycling compartment that was positive for P53 and distinct from Golgi proteins (Lippincott-Schwartz et al, 1990). Later, the Balch group found that COPI is enriched in a P58-positive pre-Golgi intermediate, which was named vesicular tubular cluster (VTC) (Aridor et al, 1995; Peter et al, 1993).

Together, these early findings provided definitive molecular evidence for the existence of a specialized intermediate organelle involved in ER-to-Golgi trafficking. This structure has been variably referred to as the ERGIC (Appenzeller-Herzog and Hauri, 2006), intermediate compartment (IC) (Saraste and Marie, 2018), or VTCs (Aridor et al, 1995). In this review, we will uniformly refer to it as the ERGIC, which is proposed by Hauri’s group and is more broadly utilized in the literature. The murine P58 and the human P53 (later designated ERGIC-53) remain canonical markers of the ERGIC based on their high abundance and stable association with ERGIC membranes.

## Subcellular localization and morphological characteristics of the ERGIC

As discussed above, early ultrastructural analyses by electron microscopy identified the ERGIC as clusters of vesicular–tubular structures positioned adjacent to the cis-Golgi (Lotti et al, 1992; Saraste and Svensson, 1991; Sesso et al, 1994). In 2005, live-cell fluorescence microscopy studies revealed that the ERGIC is highly dynamic, undergoing continuous morphological remodeling while actively participating in ER-to-Golgi transport (Ben-Tekaya et al, 2005). These dynamic and functional properties have been documented across multiple systems, including mammals (Presley et al, 1997), yeast (Tojima et al, 2024), and plants (Fougere et al, 2025).

The mechanisms underlying the maintenance of the ERGIC as a distinct organelle remain poorly understood. Classical models depict the ERGIC as a transient station formed by the convergence of COPII- and COPI-mediated transport carriers. However, a dedicated molecular framework responsible for preserving ERGIC identity has not been clearly defined. Recent evidence has identified the ubiquitously expressed protein TUG/UBXN9 as a key contributor to ERGIC organization. Loss of TUG compromises ERGIC membrane integrity, leading to the redistribution of resident ERGIC markers toward the cis-Golgi and an acceleration of anterograde trafficking. These findings support a model in which TUG functions as a molecular organizer that stabilizes the ERGIC as a structurally and functionally distinct compartment within the early secretory pathway (Parchure et al, 2025).

**Figure 1:**
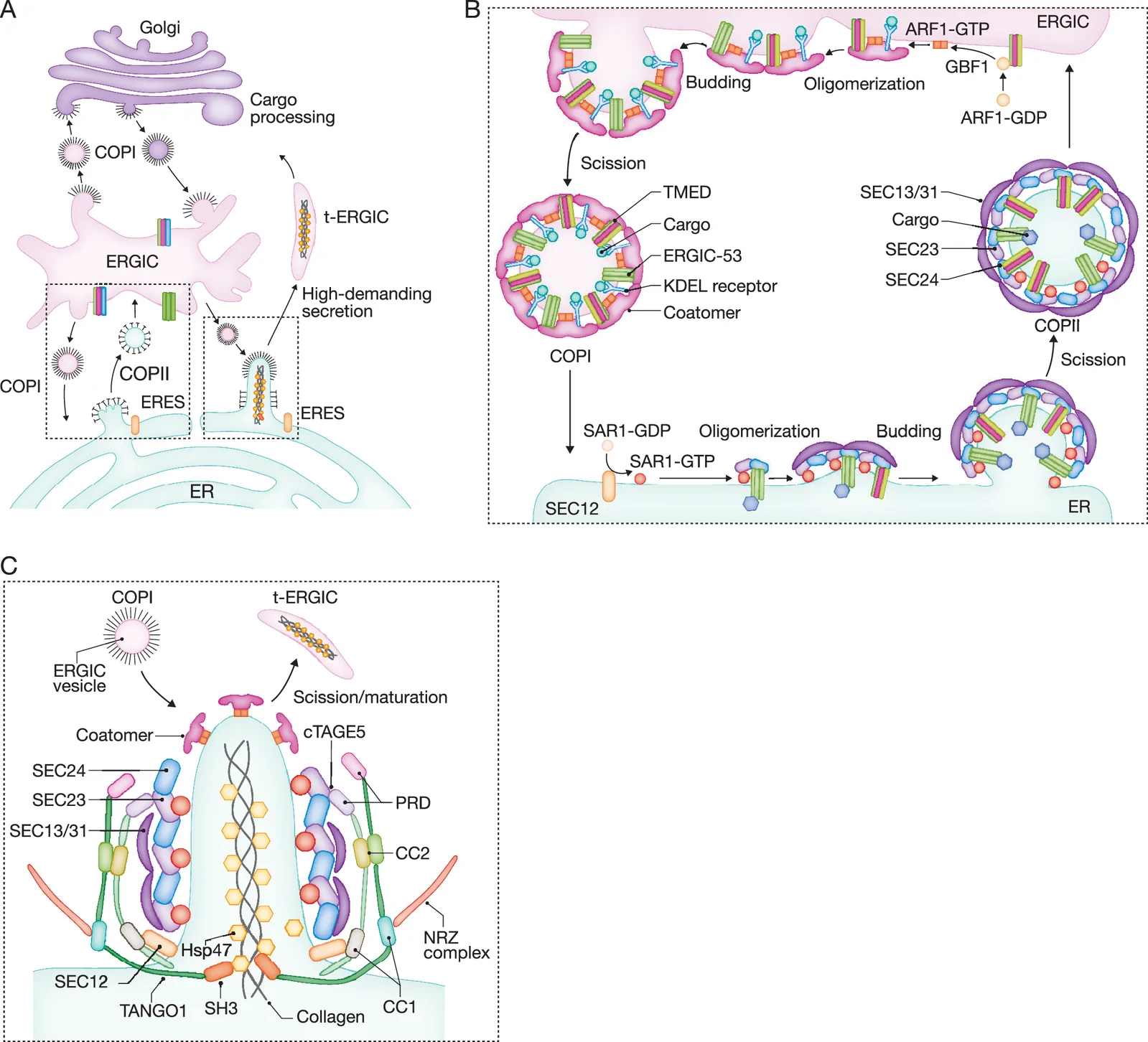
The ER–ERGIC–Golgi vesicular trafficking system. (**A**) Proteins and lipids exit the endoplasmic reticulum (ER) through COPII-coated vesicles generated at ERES, which are subsequently transported to the ERGIC. Upon arrival at the ERGIC, cargoes are sorted and further transported in the anterograde or retrograde direction via COPI-coated vesicles. (**B**) Enlarged inset showing the detailed mechanism of COPII and COPI vesicle trafficking between the ER–ERGIC interface. (**C**) Enlarged inset showing secretion of large cargoes. In addition to conventional COPII vesicles, ERES can also produce larger cargo carriers, which form the t-ERGIC to accommodate bulky cargoes. The first coiled-coil domain of TANGO1 (CC1), with the assistance of the NRZ complex, is thought to recruit additional membrane components required for the formation of t-ERGIC.

With the advent of super-resolution imaging, the ERGIC can now be interrogated with unprecedented spatiotemporal resolution. Using stochastic optical reconstruction microscopy (STORM), Xu and colleagues identified a previously unrecognized tubular ERGIC membrane domain, termed tubular ERGIC (t-ERGIC), which is marked by the cargo receptor SURF4 (Yan et al, 2022). The t-ERGIC exhibits a high surface-to-volume ratio, pronounced intracellular dynamics, and an enhanced capacity for bidirectional transport between the ER and Golgi (Fig. 1A). Functionally, this tubular compartment is required for efficient SURF4-dependent cargo trafficking (Yan et al, 2022). Tubular ERGIC-like transport carriers have also been observed under conditions of high secretory load, when bulk cargo is exported from ER exit sites (ERES). These transport carriers contain both COPII and COPI coat proteins, showing specific regional localization, which is described in more detail below (see the section “Transport of large cargoes and the t-ERGIC”) (Shomron et al, 2021; Weigel et al, 2021). Mechanistically, Ji and colleagues further demonstrated that Sec23IP mediates the recruitment of the lipid channel complex VPS13B/COH1 to the ER exit site–Golgi interface, a process essential for t-ERGIC biogenesis. Disruption of this recruitment abolishes t-ERGIC formation and markedly impairs ER-to-Golgi transport, underscoring a central role for this machinery in ERGIC morphogenesis and carrier function (Du et al, 2024).

Beyond morphological diversity, recent studies reveal pronounced functional and molecular heterogeneity within the ERGIC. In particular, super-resolution imaging has demonstrated nanoscale functional compartmentalization, showing that distinct ARF GTPase paralogs segregate into discrete membrane domains within the ERGIC. ARF4 and ARF5 define a class of ERGIC elements that lack ARF1, suggesting differential capacities for COPI recruitment and membrane trafficking regulation at this interface. At the ERGIC, ARF1 is proposed to primarily regulate retrograde transport, whereas ARF4 and ARF5 cooperate in anterograde trafficking (Ben-Tekaya et al, 2010; Wong-Dilworth et al, 2023). Consistent with this functional segregation, additional studies have identified two ERGIC subregions marked by ERGIC-53 and TMED10, which preferentially support conventional and unconventional secretion, respectively (Sun et al, 2024). This partitioning is regulated by Rab2A and the motor protein KIF5B (see “The function of ERGIC in unconventional protein secretion” for details). Collectively, these findings establish the ERGIC as a dynamic, multifunctional compartment whose activities are governed by coordinated morphological remodeling, regional specialization, and molecular segregation.

Revisiting the historical context of ERGIC discovery, its subcellular localization and tubulovesicular morphology—positioned between the ER and Golgi—are now well-established (Klumperman et al, 1998; Saraste et al, 1987; Schweizer et al, 1988). However, due to its highly dynamic behavior and context-dependent structural variability, characterizing ERGIC remains technically challenging. With continued advances in super-resolution fluorescence microscopy, electron microscopy, and correlative imaging approaches, it is anticipated that future studies will provide increasingly refined, quantitative, and multidimensional insights into the morphology and organization of this unique and functionally essential compartment.

## The role of ERGIC in ER–Golgi vesicle transport

In the ER, numerous secretory proteins, membrane proteins, and lipids are synthesized and then transported to specific intracellular destinations via the ER-to-Golgi vesicle transport pathway (Palade, 1975; Schekman and Orci, 1996). Acting as a “transit station” between the ER and Golgi, ERGIC plays a vital role in regulating molecular sorting and vesicular trafficking (Fig. 1A). During transport, cargoes are first gathered at ERES (Farhan et al, 2025), packaged into COPII-coated vesicles, and then sent to the ERGIC (Bannykh et al, 1996; Barlowe et al, 1994). In parallel, COPII vesicles can undergo homotypic fusion to generate ERGIC-like compartments, and COPII tethering and fusion events may occur at or near ERES, suggesting that ERGIC formation and cargo delivery are not strictly linear processes (Ben-Tekaya et al, 2005; Cai et al, 2007; Xu and Hay, 2004). An in-depth discussion of ERES organization and its role as a biosynthetic gateway can also be found in a recent comprehensive review (Aridor, 2026). The ERGIC serves as a key sorting station that determines the transport route of these cargoes. For example, if ER-resident proteins (such as those with a KDEL signal) are mistakenly transported to the ERGIC, the ERGIC generates COPI-coated vesicles to mediate retrograde transport, returning them to the ER (Lorente-Rodriguez and Barlowe, 2011; Yamamoto et al, 2001). Conversely, for correctly sorted molecules, the ERGIC delivers them forward via COPI vesicles to the cis-Golgi, from which they are routed to specific destinations like lysosomes, the plasma membrane, or secreted extracellularly (Bethune and Wieland, 2018). This dual-directional sorting mechanism ensures the accurate and efficient delivery of functional molecules to their intended cellular destinations, thereby maintaining the integrity and coordination of diverse intracellular systems (Lee et al, 2004; Rothman and Wieland, 1996).

### Bidirectional transport at the ERGIC: coats and small GTPases

COPII vesicles mediate transport from the ER to the ERGIC, constituting the first step of the mammalian secretory pathway. COPII vesicles form at the ERES, which is positioned in close proximity to the ERGIC (Brandizzi and Barlowe, 2013). This spatial organization is maintained by oligomerization of the Trk-fused gene (TFG) protein, which tethers ERES to the ERGIC (Johnson et al, 2015). Beyond serving as a structural scaffold, TFG has been shown to promote short-range, directional COPII vesicle transport via phase separation, generating dynamic protein condensates that guide vesicles toward the ERGIC independently of molecular motors (Qiu et al, 2024).

COPII coat assembly is initiated by activation of the small GTPase SAR1 by its guanine nucleotide exchange factor SEC12 at ERES (Lee et al, 2005). GTP-bound SAR1 recruits the SEC23–SEC24 heterodimer to form the inner coat, with SEC24 acting as the membrane cargo (e.g., ERGIC-53 and TMEDs) adapter, which directly binds the cytosolic COPII binding motifs of these cargoes (Miller et al, 2003). Subsequent recruitment of the SEC13–SEC31 complex generates the outer coat, whose polymerization drives vesicle budding and scission (Zanetti et al, 2011) (Fig. 1B). GTP hydrolysis on SAR1, stimulated by SEC23 and further accelerated by SEC31, regulates coat dynamics (Antonny et al, 2001). After vesicle release, TFG promotes disassembly of the outer coat, while the inner coat remains intact and interacts with the TRAPP tethering complex to facilitate targeting to the ERGIC (Cai et al, 2007; Hanna et al, 2017). Vesicle fusion with the ERGIC is ultimately mediated by SNARE proteins (Sudhof and Rothman, 2009; Zanetti et al, 2011).

From the ERGIC, cargoes can be transported anterogradely to the Golgi or recycled back to the ER via COPI vesicles (Lord et al, 2013; Scales et al, 1997). COPI vesicle formation depends on activation of the GTPase ARF1 by SEC7-domain-containing GEFs such as GBF1, which localize to the ERGIC and cis-Golgi (Claude et al, 1999; Saenz et al, 2009; Zhao et al, 2006). TMED proteins can bind and recruit ARF1-GDP to membranes to facilitate its activation (Gommel et al, 2001). GTP-bound ARF1 recruits coatomer to membranes. The coatomer is a heptameric complex, composed of seven COPI subunits arranged into inner and outer layers which directly bind to membrane cargoes like TMEDs at the cytosolic face (Beck et al, 2009; D’Souza-Schorey and Chavrier, 2006). This recruitment and coat assembly process is well-established in cell-free systems, although direct dynamic visualization in intact cells remains limited. Recent evidence indicates that ARF1 can also drive the formation of retrograde tubular carriers (rather than classical COPI vesicles) through a Src-GBF1 pathway, where Src phosphorylates GBF1 on multiple tyrosine residues to enhance its interaction with ARF1 and promote tubular transport intermediates (Chia et al, 2021). Whether these retrograde tubular carriers integrate into or become part of the ERGIC has not yet been experimentally tested (Chia et al, 2021). As with COPII, GTP hydrolysis on ARF1 triggers coat disassembly, enabling vesicle fusion (Tanigawa et al, 1993). Multiple ARF isoforms and COPI subunit variants contribute to transport regulation and sorting specificity, although their precise roles remain incompletely defined (Popoff et al, 2011b; Volpicelli-Daley et al, 2005). Together, COPII-mediated ER export and COPI-mediated bidirectional trafficking between the ERGIC and Golgi constitute the classical vesicular transport model (Fig. 1B).

Efficient ER–Golgi trafficking further relies on Rab small GTPases, particularly Rab1 and Rab2, which localize to the ERGIC and Golgi (Saraste, 2016). Through interactions with tethering factors such as TRAPP, GM130, GRASP55/65, p115, and giantin, Rab1/2 coordinate vesicle tethering, fusion, and compartment organization (Saraste, 2016). They also regulate coat recruitment and COPI vesicle biogenesis, thereby integrating anterograde and retrograde transport (Monetta et al, 2007; Tisdale and Jackson, 1998).

Beyond classical secretion, Rab1 and Rab2 play important roles in autophagy and unconventional secretion. Rab1 regulates ERGIC-derived pre-autophagosomal structures and autophagosome maturation (Wang et al, 2013; Zoppino et al, 2010), whereas Rab2 controls autophagosome biogenesis and autophagosome–lysosome fusion via interactions with ULK1 and downstream effectors (Ding et al, 2019; Lorincz et al, 2017). In addition, Rab1A-positive ERGIC compartments participate in Golgi-bypassing unconventional secretion (Marie et al, 2009), and Rab1/2 cooperate at the ERGIC to activate a TMED10-dependent unconventional secretion pathway (Sun et al, 2024). These diverse functions highlight the ERGIC as a central hub integrating vesicular trafficking, autophagy, and unconventional protein secretion.

### Transport of large cargoes and the t-ERGIC

Although the ERGIC is classically viewed as the recipient of COPII vesicles, accumulating evidence indicates that it also plays an active regulatory role in shaping COPII carrier biogenesis, particularly during the situation of high-demanding secretion, e.g., secretion of large cargoes (Malhotra and Erlmann, 2015; Miller and Schekman, 2013). Under standard conditions, COPII vesicles are relatively small, with diameters of ~60–80 nm. However, the export of oversized cargoes such as procollagen—whose length can reach 300–400 nm—requires specialized mechanisms to expand COPII carrier size and capacity (McCaughey and Stephens, 2019).

Multiple regulatory strategies have been identified that enable the formation of enlarged COPII carriers. These include ubiquitination of the outer-coat component SEC31 (Jin et al, 2012), selective utilization of SAR1 paralogs (SAR1A versus SAR1B) (Levy et al, 2019), and the action of dedicated cargo receptors such as TANGO1 (Transport and Golgi Organization Protein 1) (Saito et al, 2009). A unifying feature of these mechanisms is likely their ability to slow SAR1 GTP hydrolysis, thereby prolonging COPII coat assembly and delaying vesicle scission to allow carrier expansion.

TANGO1, identified by the Malhotra group, plays a central role in this process. Localized at ERES, TANGO1 coordinates cargo recruitment and COPII coat regulation across the ER membrane (Raote et al, 2018; Saito et al, 2009) (Fig. 1C). On the luminal side, its SH3 domain binds the collagen chaperone Hsp47, facilitating selective recruitment of procollagen (Ishikawa et al, 2016). More recently, the luminal SH3 domain of TANGO1 has also been shown to bind TMED family proteins, and their interaction regulates the ER–Golgi interface organization and efficient cargo transport (Yang et al, 2024). On the cytoplasmic side, TANGO1 engages cTAGE5 through its coiled-coil domain, and both proteins contain C-terminal proline-rich domains (PRDs) that interact with the SEC23–SEC24 inner COPII coat (Saito et al, 2011). This interaction competitively inhibits the association of SEC13–SEC31 with SEC23–SEC24, suppressing SAR1-GTP hydrolysis and thereby delaying vesicle budding (Ma and Goldberg, 2016). Functional disruption of this TANGO1–cTAGE5 module, either by peptide inhibitors or by perturbing PRD–SEC23A interactions, selectively impairs collagen secretion and alters ERES organization, highlighting its importance in exporting bulky cargoes and functionally segregating ERES by cargo size (Raote et al, 2024; Saxena et al, 2024) (Fig. 1C).

As COPII carriers enlarge, the requirement for additional membrane material becomes increasingly acute. Recent studies suggest that this demand is met, at least in part, by membrane input from the ERGIC (Raote et al, 2018; Santos et al, 2015). In addition to regulating SAR1 activity, TANGO1 directly promotes ERGIC recruitment to sites of large COPII carrier formation (Raote et al, 2018; Santos et al, 2015). The first coiled-coil domain of TANGO1 contains the TEER (tether for ERGIC at the ER) motif, which interacts with the NRZ tethering complex (NBAS–RINT1–ZW10) (Santos et al, 2015). Through this interaction, ERGIC membranes are positioned in close proximity to collagen-containing ERES, potentially serving as a membrane reservoir to support COPII carrier expansion (Raote et al, 2018; Santos et al, 2015) (Fig. 1C). However, the identity of the ERGIC membrane proteins that engage the TEER–NRZ machinery, the mechanism by which ERGIC membranes fuse with nascent COPII carriers, and how membrane flux from the ERGIC is regulated remain open questions.

Parallel insights from live-cell imaging suggest that, under conditions of high secretory demand, a hybrid transport architecture can form between ERES and the ERGIC in the form of tunnel-like carriers (the precursor of t-ERGIC) (Weigel et al, 2021). COPII components are largely confined to the neck region of ERES, where they function primarily in cargo selection and concentration rather than coating the entire transport carrier. In contrast, secretory cargoes are frequently detected within elongated tubular or continuous membrane structures extending from ERES that lack detectable COPII coats along their length (Shomron et al, 2021; Weigel et al, 2021). In these tubular carriers, COPII components localize predominantly to the basal region at the ERES interface, where they are thought to regulate cargo sorting and membrane remodeling. By contrast, COPI coats decorate more distal regions of the tubular ERGIC. This spatial separation of COPII and COPI along tubular carriers supports the proposed tunnel model, in which elongated ERES forms with COPII concentrated at the base and COPI at the distal end. These structures extend from the ER and give rise to t-ERGIC tubules, which act as conduits for directional cargo transport toward the Golgi apparatus (Shomron et al, 2021; Weigel et al, 2021) (Fig. 1C).

These observations have given rise to the tunnel model, in which ERES generate extended membrane conduits that either mature into transport carriers or establish transient, tunnel-like connections with downstream compartments such as the ERGIC (Raote et al, 2018). Within this framework, the ERGIC functions not only as a post-ER sorting station but also as a direct acceptor—or extension—of ERES-derived membranes, thereby facilitating the efficient export of oversized cargoes such as collagen and mucins (Raote and Malhotra, 2021; Raote et al, 2018; Yuan et al, 2018) (Fig. 1A,C).

While the tunnel model offers an attractive explanation for large-cargo export, several key questions remain unresolved. How receptor–cargo complexes are transferred from COPII-enriched ERES into COPI-positive or ERGIC-associated membranes is still unclear. In addition, the mechanisms by which ERES dynamically remodel their geometry to accommodate oversized cargoes, and the precise contribution of COPI to forward transport within this pathway, remain to be elucidated.

Taken together, these findings suggest that ER-to-Golgi transport employs a spectrum of carrier types—including vesicular, tubular, and tunnel-like intermediates—rather than a single uniform mechanism (Raote and Malhotra, 2021; Raote et al, 2018; Weigel et al, 2021). The ERGIC emerges as a central and dynamic hub within this system, functioning not only as a recipient of COPII carriers but also as a membrane donor and organizational platform for large-cargo export (Raote and Malhotra, 2021). This versatility likely endows the early secretory pathway with the robustness and adaptability needed to accommodate diverse cargo sizes and cellular demands, and may help reconcile seemingly conflicting observations reported across different experimental systems.

### Cargo receptors in ERGIC-mediated protein sorting

The functional complexity of the cell relies on the accurate delivery of distinct proteins to their designated destinations. While COP-coated vesicles provide the driving force for membrane trafficking at the ERGIC, the selective recognition and sorting of cargoes within these vesicles are equally critical for maintaining cellular homeostasis. In this context, a series of receptor molecules contribute to vesicular transport by mediating selective cargo recognition, thereby promoting the efficient dispatch of specific cargoes in space and time. Importantly, cargo receptors are not required for the existence of the ERGIC itself, but rather fine-tune cargo sorting for particular classes of proteins. These receptors contribute to cargo specificity through their unique physicochemical properties, highly specific interaction motifs, and finely regulated dynamics.

#### ERGIC-53

ERGIC-53, also known as LMAN1 (lectin mannose-binding protein 1), is one of the earliest identified ERGIC-resident marker proteins and functions as a cargo receptor that mediates the export of soluble glycoproteins from the ER (Appenzeller et al, 1999; Hauri et al, 2000; Schindler et al, 1993; Schweizer et al, 1988) (Fig. 1B). As a type I transmembrane protein, ERGIC-53 harbors a KKFF motif at its cytoplasmic C-terminal tail, which facilitates its interaction with COPI coat proteins and enables its selective packaging into COPII vesicles via specific Sec24 paralogs (Wendeler et al, 2007), enabling its participation in bidirectional transport cycles between the ER, ERGIC, and cis-Golgi compartments (Kappeler et al, 1997; Tisdale et al, 1997). The requirement of this C-terminal sorting motif and oligomerization for ERGIC-53 localization and function is evolutionarily conserved (Nie et al, 2018); for example, its fission yeast orthologue Emp43 relies on a C-terminal KYL motif for proper ERGIC targeting (Imamura et al, 2025).

On the luminal side, the N-terminal ectodomain of ERGIC-53 contains a lectin-like domain that binds mannose residues in a Ca²⁺-dependent manner (Itin et al, 1996). Structural studies of the lectin-like domain have revealed that ERGIC-53 belongs to the L-type lectin family and displays a relatively broad specificity for high-mannose glycans, distinguishing it from related cargo receptors such as VIP36 (Satoh et al, 2014). Moreover, the interaction between ERGIC-53 and its cargo (e.g., pro-cathepsin Z) is regulated in a pH-dependent manner (Appenzeller-Herzog et al, 2004). Specifically, cargo binding occurs in the high-pH and high-Ca²⁺ environment of the ER, while dissociation is triggered in the acidic, low-Ca²⁺ milieu of the ERGIC (Appenzeller-Herzog and Hauri, 2006; Saraste and Marie, 2018). This mechanism enables spatially and temporally coordinated cargo capture and release, which is critical for the fidelity of the intracellular protein transport system.

Early biochemical evidence indicates that newly synthesized ERGIC-53 molecules can assemble into dimers or hexamers within the ER (Hauri et al, 2000; Nufer et al, 2003). Recent structural work has substantially revised the oligomerization model of ERGIC-53. Cryo-EM analysis by Inaba and colleagues has resolved the structure of full-length ERGIC-53 in complex with its functional partner MCFD2, establishing ERGIC-53 as a homotetramer with a four-leaf clover head domain and a long, flexible stalk, overturning the previously proposed hexamer model. These findings provide new structural insights into the molecular architecture of the ERGIC-53–MCFD2 complex (Watanabe et al, 2024). The functional relevance of ERGIC-53 oligomerization and lectin activity is further underscored by genetic and disease-associated studies. Mutations in ERGIC-53 or MCFD2 underlie combined deficiency of coagulation factors V and VIII (F5F8D) (Nyfeler et al, 2008). Structural analyses further revealed that MCFD2 binds ERGIC-53 at a site distinct from the sugar-binding pocket, allowing the two proteins to engage cargo through complementary mechanisms: glycan recognition by ERGIC-53 and cargo-selective recognition by MCFD2 (Nishio et al, 2010). Zn²⁺ binding to the N-terminal lid of MCFD2 appears to further modulate cargo binding dynamics at the ER–Golgi interface (Watanabe et al, 2024). Thus, MCFD2 serves dual roles—acting as a cargo-selective co-receptor while also regulating cargo binding through Zn²⁺ coordination. Together, these features enable the ERGIC-53–MCFD2 complex to couple broad glycan recognition with cargo-specific selectivity, thereby enhancing transport efficiency and specificity.

#### p24/TMED (transmembrane emp24 domain) protein family

The p24/TMED (transmembrane emp24 domain) protein family comprises a group of type I transmembrane proteins that are broadly conserved across eukaryotes and play essential roles in protein transport and sorting from the ER to the Golgi apparatus (Strating and Martens, 2009). In vertebrates, this family consists of ten members, which can be categorized into four subfamilies based on sequence similarity: α (TMED4/9/11), β (TMED2), γ (TMED1/3/5/6/7), and δ (TMED10) (Aber et al, 2019; Pastor-Cantizano et al, 2016). Interestingly, the human TMED11 gene may not encode a functional protein due to a premature stop codon (Strating et al, 2009). TMED proteins are capable of forming various hetero-oligomeric complexes, such as TMED2–TMED10 heterodimers or cross-subfamily heterotetramers, which are critical for their proper intracellular localization and function (Pastor-Cantizano et al, 2016; Strating and Martens, 2009).

Structurally, TMED family members share conserved features, including a GOLD (Golgi dynamics) domain, a coiled-coil (CC) domain, a single transmembrane domain, and a short cytoplasmic tail (Pastor-Cantizano et al, 2016). The GOLD and CC domains are believed to mediate both cargo recognition and hetero-oligomerization among TMED proteins (Nagae et al, 2016; Theiler et al, 2014). Notably, the transmembrane domain of TMED2 selectively interacts with the sphingolipid SM18, which influences its dimerization (Contreras et al, 2012; Pannwitt et al, 2019). The cytoplasmic tail of TMED proteins contains conserved motifs resembling those found in ERGIC-53, enabling their interaction with COPI and COPII vesicles and facilitating efficient cycling between the ER, ERGIC, and cis-Golgi compartments (Dominguez et al, 1998). Members of the TMED family function as cargo receptors for a variety of proteins (Fig. 1B), including glycosylphosphatidylinositol (GPI)-anchored proteins (GPI-APs), Wnt proteins, G protein-coupled receptors (GPCRs), and Toll-like receptors (Pastor-Cantizano et al, 2016). Recent work has further identified TMED9 as a key quality-control receptor for misfolded GPI-anchored proteins, capturing aberrant GPI-APs released from calnexin in the ER and promoting their ER export via the RESET pathway, thereby enabling downstream Golgi trafficking and lysosomal degradation (Ronzier and Satpute-Krishnan, 2025). TMED10 has emerged as a regulator of selective cargo sorting in the early secretory pathway, directly controlling the incorporation of insulin-like growth factor 2 (IGF2) into COPII vesicles at the ER and modulating cargo receptor trafficking at the trans-Golgi network (TGN)(Li et al, 2023). Consistent with this role, TMED10 functions as a co-receptor during ER export by cooperating with the cargo receptor SURF4 to selectively recruit distinct SEC24 paralogs, thereby enabling cargo-specific COPII coat engagement and efficient secretion of defined soluble proteins (Maldutyte et al, 2025). Through both direct and indirect mechanisms, TMED10 influences IGF2 secretion and thereby affects myogenic differentiation.

In addition, TMED proteins participate in the formation of COPI vesicles (Popoff et al, 2011a), contribute to the structural organization of intracellular membrane systems, particularly in shaping the ERGIC and Golgi apparatus (Pastor-Cantizano et al, 2016; Strating and Martens, 2009), and participate in cargo translocation at the ERGIC (Zhang et al, 2020b; Zheng et al, 2026a). These multifaceted roles underscore the importance of the TMED family in maintaining the complexity and fidelity of the intracellular protein trafficking network.

#### KDEL receptor

The KDEL receptor, a seven-transmembrane domain protein, plays a pivotal role in the retrograde transport of proteins from the Golgi back to the ER. Its cytoplasmic C-terminal di-lysine motif mediates interaction with COPI vesicles, a process that is regulated by phosphorylation of a nearby serine residue (S209) by protein kinase A (PKA) (Cabrera et al, 2003). On the luminal side, the KDEL receptor recognizes and binds soluble proteins bearing a C-terminal KDEL signal, thereby facilitating their retrieval to the ER via COPI-coated vesicles (Lewis and Pelham, 1992). The interaction between the KDEL receptor and its cargo is pH-dependent: the mildly acidic environment of the ERGIC and Golgi (pH 6.5–6.8) induces conformational changes enabling cargo binding, whereas in the relatively alkaline ER lumen (pH 7.2–7.4), cargo dissociates from the receptor (Gomez-Navarro and Miller, 2016; Wilson et al, 1993). In addition, the KDEL receptor has been shown to interact with TMED family proteins to promote COPI vesicle formation (Pastor-Cantizano et al, 2016). In mammals, three isoforms of the KDEL receptor exist, and their differential cargo specificity enhances the efficiency of retrograde transport (Raykhel et al, 2007).

Beyond its role in protein retrieval, the KDEL receptor contributes to the homeostasis of anterograde and retrograde transport between the ER and Golgi (Cancino et al, 2014). Intriguingly, it also functions as a signaling molecule: by activating the small GTPase Gαo, it participates in vesicular trafficking pathways related to neurite outgrowth and cellular morphogenesis (Solis et al, 2017).

### ERGIC and protein quality control

The ERGIC works in concert with the ER to monitor protein folding and assembly, ensuring that only properly folded and functional proteins are transported to the Golgi apparatus (Saraste and Marie, 2018; Saraste and Marie, 2015). Glucosidase II (GlsII) and UDP-glucose glycoprotein glucosyltransferase play essential roles in the quality control of glycoproteins (Roth and Zuber, 2017). Immunoelectron microscopy studies have shown that both enzymes are not only abundantly present in the ER but also relatively enriched in the ERGIC, while they are scarcely distributed in the Golgi (Lucocq et al, 1986; Zuber et al, 2001). These findings suggest that ERGIC may also participate in glycoprotein quality control processes.

In addition, the ERGIC contains multiple molecular chaperones bearing the KDEL retention motif, such as immunoglobulin-binding protein (BiP), glucose-regulated protein 94 (GRP94), protein disulfide isomerase (PDI), and calreticulin (Breuza et al, 2004; Zuber et al, 2001). These proteins are responsible for retrieving misfolded proteins back to the ER for refolding and quality control, a process mediated by KDEL receptors (Saraste and Marie, 2018). The retrograde transport of misfolded tsO45-VSV-G proteins to the ER, for example, is regulated by the molecular chaperone BiP and involves the ERGIC (Hammond and Helenius, 1994). In addition, BiP and the ERp44 protein of the PDI family can bind incompletely assembled T-cell antigen receptor α chain (TCRα) and immunoglobulin M (IgM), returning them to the ER via KDEL receptor-mediated retrieval (Vavassori et al, 2013; Yamamoto et al, 2001). The retrieval transport of BiP is not only associated with neurodevelopment but is also closely linked to certain neurodegenerative diseases (Jin et al, 2017).

More broadly, ERGIC-mediated retrieval serves as an important quality control mechanism for membrane proteins, and can occur through mechanisms distinct from BiP-mediated chaperone retrieval. Interestingly, some mutant proteins—such as the V2 vasopressin receptor and the neurodegeneration-associated protein TREM2—can reach the ERGIC but fail to proceed to the plasma membrane. Instead, they are returned to the ER via COPI-dependent retrograde transport (Hermosilla et al, 2004; Sirkis et al, 2017). Moreover, several misfolded proteins—including the ΔF508 mutant of CFTR, MHC class I molecules, and proinsulin—have been reported to accumulate within the ERGIC (Gilbert et al, 1998; Saraste and Marie, 2015). There is also evidence indicating that the ERGIC is involved in the ubiquitination and degradation of misfolded MHC-I molecules (Raposo et al, 1995).

Beyond its role as a passive checkpoint for misfolded proteins, emerging evidence indicates that ERGIC-resident cargo receptors actively regulate protein quality control decisions. In particular, pharmacological targeting of the ERGIC-localized cargo receptor TMED9 has revealed a previously unrecognized mechanism by which misfolded secretory proteins are selectively retained and cleared. Greka and colleagues demonstrated that a disease-causing frameshift mutant of mucin-1 (MUC1-fs), responsible for autosomal dominant tubulointerstitial kidney disease, accumulates within TMED9-positive early secretory vesicles rather than being efficiently secreted or degraded. A small molecule, BRD4780, was shown to bind TMED9, disrupt its cargo-retention function, and release MUC1-fs from TMED-containing compartments, thereby redirecting the misfolded protein toward lysosomal degradation. Genetic deletion of TMED9 phenocopied the effects of BRD4780, establishing TMED9 as a key determinant of misfolded cargo entrapment within the early secretory pathway (Dvela-Levitt et al, 2019; Xiao et al, 2024). These findings provide direct functional evidence that TMED-mediated retention at the ER–ERGIC interface represents a modifiable node in protein quality control, linking cargo receptor function to lysosomal clearance pathways rather than ER-associated degradation alone(Dvela-Levitt et al, 2019). In addition, several TMED proteins have been shown to be involved in the clearance of misfolded GPI-anchored proteins (Ronzier and Satpute-Krishnan, 2025; Satpute-Krishnan et al, 2014; Zavodszky and Hegde, 2019). Extending this concept to human disease, recent work demonstrated that TMED2, TMED9, and TMED10 directly bind the secretory glycoprotein uromodulin (UMOD) and regulate its trafficking along the early secretory pathway. Pharmacological targeting of TMED cargo receptors alleviated intracellular retention of mutant UMOD, restored its apical secretion in patient-derived kidney organoids and knock-in mouse models, and mitigated ER stress and renal pathology, highlighting TMED proteins as therapeutically actionable cargo receptors in protein misfolding diseases (Bazua-Valenti et al, 2024).

## Regulation of autophagy by the ERGIC

Macroautophagy (hereafter referred to as autophagy) is a conserved intracellular catabolic pathway that involves the formation of a double-membrane vesicle known as the autophagosome (Nakatogawa, 2020). During autophagy, cellular components—including nucleic acids, proteins, and damaged organelles—are sequestered by autophagosomes and subsequently delivered to lysosomes (or the vacuole in yeast and plants) for bulk degradation (Nakatogawa, 2020). Autophagy plays a central role in maintaining cellular homeostasis and responding to various stress conditions. Dysregulation of autophagy has been implicated in a wide range of diseases, including neurodegenerative disorders, cancer, and diabetes (Levine and Kroemer, 2019).

### The ERGIC as a membrane source for autophagosome formation

**Figure 2:**
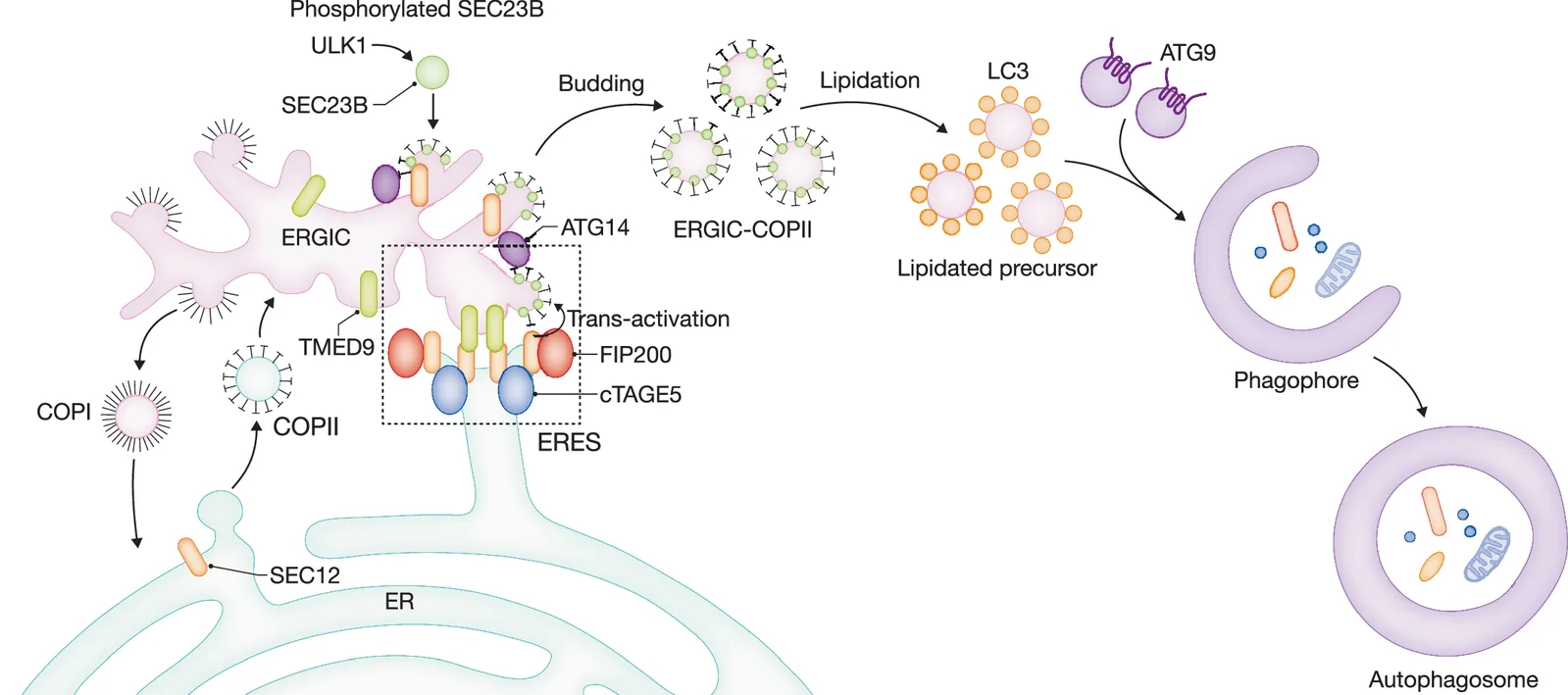
A model for ERGIC-mediated generation of autophagosome precursors. Under starvation conditions, ERES are enlarged and establish close interactions with the ERGIC, a process that depends on FIP200, cTAGE5, and the interaction between TMED9 and SEC12. Remodeling of ERES leads to the relocation of SEC12 to the ERGIC, thereby promoting the assembly of ERGIC-derived COPII vesicles (ERGIC-COPII vesicles). In this process, ULK1-dependent regulation of COPII components, including Sec23B, facilitates vesicle budding from the ERGIC. In addition, SEC12 remaining at the ERES can trans-activate the generation of ERGIC-COPII vesicles. These ERGIC-COPII vesicles serve as substrates for LC3 lipidation, and the orange dots shown on the lipidated precursors represent lipidated LC3. Together with other membrane sources, such as ATG9 vesicles, these LC3-positive precursors contribute to phagophore expansion and autophagosome formation.

The formation of autophagosomes is a critical step in the autophagic process that requires membrane contribution from the endomembrane system, which likely involves multiple membrane sources depending on cellular states (Ge et al, 2014a; Lamb et al, 2013; Nakatogawa, 2020). Recent studies have begun to shed light on the dynamic involvement of the ERGIC as a major membrane source during the early stages of autophagosome formation (Ge et al, 2014a, 2013, 2017) (Fig. 2).

To establish a functional connection between intracellular membranes and autophagosome biogenesis, Schekman and colleagues developed in vitro reconstitution systems that recapitulate autophagic regulation, including starvation-induced responses, PI3K signaling, and ULK complex activation. Using this approach, the investigators evaluated the capacity of various organelle-derived membrane fractions to support LC3 lipidation—a key molecular event in autophagosome formation (Brier et al, 2016; Kabeya et al, 2000). Surprisingly, classical organelles previously implicated in autophagy, such as the ER, Golgi, and mitochondria, exhibited relatively weak LC3 lipidation activity. In contrast, the ERGIC demonstrated strong LC3 lipidation capacity, implicating it as a major contributor to autophagosome membrane biogenesis (Ge et al, 2013; Ge and Schekman, 2014; Han et al, 2023a) (Fig. 2).

Super-resolution imaging has revealed close spatial associations between ERGIC membranes and autophagy-related proteins, including ATG13, a core component of the ULK1 complex, as well as ATG9-positive vesicles during the early stages of autophagy (Karanasios et al, 2016). These observations suggest that ERGIC-derived membranes cooperate with ATG9 vesicles to promote phagophore nucleation (Karanasios et al, 2016)(Fig. 2). These findings identify the ERGIC as an important membrane reservoir for autophagosome biogenesis under diverse autophagic stimuli. Consistently, using LC3 lipidation-based assays, Chen and colleagues demonstrated that the ERGIC also functions as a platform for cGAS–STING-induced autophagy (Gui et al, 2019). Upon pathway activation, STING relocalizes to the ERGIC, effectively converting this compartment into a source of autophagosomal membrane and promoting autophagosome formation.

### ERGIC-derived COPII vesicles: the ERGIC-COPII subtype

Although the ERGIC contributes membrane material to autophagosome biogenesis, its tubulovesicular architecture is structurally distinct from the double-membrane topology of autophagosomes. This raises the question: how does the ERGIC supply a membrane suitable for autophagosome formation? Recent studies have shown that the ERGIC recruits ATG14, a critical early autophagy regulator, to initiate phosphatidylinositol 3-phosphate (PI3P) production, promote LC3 lipidation, and drive downstream autophagosome maturation (Ge et al, 2013). Activation of the PI3K pathway induces the translocation of SEC12—a key factor in COPII vesicle formation—from ERES to the ERGIC. This relocalization promotes the formation of a distinct subset of COPII vesicles derived from the ERGIC, termed “ERGIC-COPII vesicles”, which exhibit enhanced capacity for LC3 lipidation and are functionally distinct from conventional COPII vesicles that mediate ER-to-Golgi transport. These specialized ERGIC-COPII vesicles likely function as direct membrane precursors for autophagosome formation (Ge et al, 2015; Ge et al, 2014b) (Fig. 2). The role of COPII vesicles in autophagy has also been supported by other studies showing that autophagosome formation depends on SAR1 function and the integrity of ERES (Stadel et al, 2015; Zoppino et al, 2010).

Accumulating evidence has begun to elucidate the molecular mechanisms responsible for the generation of ERGIC-derived COPII vesicles. Under basal conditions, the guanine nucleotide exchange factor SEC12 is predominantly localized to ERES, where it constitutively activates Sar1 to support conventional COPII-mediated ER-to-Golgi trafficking. Although SEC12 is intrinsically active, its function can be further enhanced under specific signaling conditions. For example, the ER-resident receptor tyrosine kinase leukocyte receptor tyrosine kinase (LTK) phosphorylates SEC12 to stimulate its activity (Centonze et al, 2019). In contrast, nutrient deprivation profoundly remodels the function of SEC12 through extensive reorganization of both ERES and the ERGIC, thereby repurposing COPII machinery for autophagosome biogenesis. During starvation, the autophagy factor FIP200 reshapes ERES architecture and promotes its close spatial association with the ERGIC, while the ERES-associated protein cTAGE5 interacts with SEC12 to maintain ERES integrity and support autophagy (Ge et al, 2017). More recently, a specialized membrane contact site between the ERGIC and ERES, termed the ERGIC–ERES membrane contact, was identified and shown to be mediated by the ERGIC-resident protein TMED9. TMED9 directly interacts with SEC12 and brings the two membrane compartments into close proximity (~2–5 nm), enabling trans-activation of SEC12 and promoting COPII vesicle formation at the ERGIC (Fig. 2, boxed region) (Li et al, 2022; Li et al, 2021; Puri and Rubinsztein, 2022). This starvation-induced structural remodeling also allows a fraction of SEC12 to escape its conventional confinement to ERES and relocate to the ERGIC, where it further drives the generation of ERGIC-derived COPII vesicles. Unlike canonical COPII vesicles, which function as transport carriers in ER-to-Golgi trafficking, ERGIC-derived COPII vesicles serve as membrane templates for LC3 lipidation. Following LC3 conjugation, these vesicles become building blocks for autophagosome assembly and likely cooperate with other membrane sources, such as ATG9-positive vesicles, to initiate and expand nascent autophagosomes (Ge et al, 2013; Ge et al, 2017; Ge et al, 2014b). Additional studies have further expanded our understanding of how the COPII machinery is rewired during autophagy. Notably, starvation-induced phosphorylation of SEC23B, a component of the COPII coat, by the autophagy-initiating kinase ULK1 promotes its relocalization to the ERGIC, further facilitating autophagosome biogenesis (Jeong et al, 2018).

Collectively, these findings underscore a central regulatory role for the ERES–ERGIC–COPII membrane interface in orchestrating membrane remodeling during autophagy (Fig. 2). The ability of the ERGIC to support specialized COPII-dependent autophagic pathways critically depends on its structural integrity, as disruption of ERGIC organization—for example, through loss of the ERGIC organizer TUG—impairs ERGIC-dependent autophagy(Parchure et al, 2025). These observations highlight the ERGIC not merely as a passive membrane reservoir but as an actively maintained platform that enables productive coupling between secretory trafficking and autophagosome biogenesis.

Notably, these principles echo regulatory mechanisms first uncovered in *Saccharomyces cerevisiae*. Seminal studies by Yoshinori Ohsumi and colleagues in the early 2000s identified core COPII components, including Sec12, Sec23, and Sec24, as key regulators of both ER export and autophagy initiation (Ishihara et al, 2001). Subsequent work demonstrated that the pre-autophagosomal structure (PAS) and the isolation membrane (phagophore) form in close association with ERES (Graef et al, 2013; Suzuki et al, 2013). More recently, direct evidence showed that the yeast transmembrane protein Axl2 is transported from the ER to autophagosomes via COPII vesicles, providing compelling support for the idea that COPII-derived membranes directly contribute to autophagosome biogenesis (Shima et al, 2019). Together, these studies establish the ERES–ERGIC–COPII membrane network as an evolutionarily conserved module supporting autophagy across eukaryotes.

Precise targeting of COPII vesicles to the PAS or phagophore represents a critical step in autophagosome formation. In yeast, this process is governed by the Rab1 homolog Ypt1 and its autophagy-specific guanine nucleotide exchange factor (GEF), the TRAPP III complex (Lynch-Day et al, 2010). Ypt1 and TRAPP III localize to the PAS, where they interact with core Atg1 complex components, including Atg1 and Atg11, as well as with the transmembrane protein Atg9 (Kakuta et al, 2012; Lipatova et al, 2012; Wang et al, 2013). These interactions are essential for PAS assembly and autophagy initiation. Mechanistically, TRAPP III directly associates with the COPII coat subunit Sec23, thereby physically linking COPII vesicles to autophagic membrane platforms (Tan et al, 2013).

Beyond vesicle tethering, Ypt1 also recruits and activates the casein kinase I homolog Hrr25 at the PAS (Wang et al, 2015). Hrr25 phosphorylates the COPII subunit Sec24, enhancing its affinity for Atg9 and promoting efficient incorporation of COPII vesicles into nascent autophagosomes (Davis et al, 2016). This coordinated regulation ensures the accurate positioning of COPII vesicles at the PAS and facilitates their functional cooperation with Atg9-positive vesicles during early phagophore formation (Hurley and Young, 2017). Once the membrane is seeded at the PAS, bulk lipid transport mediated by ATG2 supports autophagosome membrane growth (Melia, 2023; van Vliet et al, 2022).

Importantly, key elements of this regulatory architecture are conserved in mammalian cells. Both the TRAPP III complex and Rab1 have been implicated in autophagosome biogenesis, further supporting the notion that COPII vesicles play a fundamental role in autophagy beyond their classical function in ER-to-Golgi transport (Lamb et al, 2016; Zoppino et al, 2010). Together, these mechanistic insights reveal an intricate and evolutionarily conserved network of molecular interactions that directs COPII vesicle targeting and membrane contribution during autophagy, providing a conceptual framework for understanding how secretory pathway components are repurposed to drive intracellular degradation pathways.

## The function of ERGIC in unconventional protein secretion

Protein secretion is an evolutionarily conserved mechanism that enables intercellular communication. In eukaryotic cells, the majority of secretory proteins contain an N-terminal signal peptide that directs their co-translational translocation into the ER through the SEC61 translocon. These proteins subsequently undergo folding, processing, and post-translational modification as they traffic through the ER–Golgi vesicular transport system before being delivered to their final destinations or secreted extracellularly (see section “The role of ERGIC in ER–Golgi vesicle transport”). This route is referred to as the conventional secretory pathway (Viotti, 2016).

In contrast, accumulating evidence demonstrates that numerous cytoplasmic proteins lacking signal peptides can also be released into the extracellular space through pathways that bypass the canonical ER-to-Golgi route. These processes are collectively termed unconventional protein secretion (UcPS) (Saraste and Prydz, 2026; Zhang and Schekman, 2013; Zheng and Ge, 2022). UcPS is often activated under conditions of cellular stress and plays a critical role in maintaining protein homeostasis. Proteins secreted via UcPS participate in diverse physiological processes, including immune responses, viral infection, and lipid metabolism, and dysregulation of UcPS has been implicated in a wide range of diseases, such as inflammation, neurodegenerative disorders, and cancer (Chen et al, 2025; Kim et al, 2018).

UcPS can be broadly classified into two major categories. The first comprises vesicle-independent mechanisms, in which cargo proteins are directly translocated across the plasma membrane. Two representative pathways have been well characterized. In one mechanism, activated gasdermin D (GSDMD) forms pores in the plasma membrane, allowing the release of small cytoplasmic proteins—particularly pro-inflammatory cytokines—into the extracellular space (Ding et al, 2016; Evavold et al, 2018); excessive pore formation ultimately leads to pyroptotic cell death (Kayagaki et al, 2015; Shi et al, 2015). In a second mechanism exemplified by fibroblast growth factor 2 (FGF2), the cargo protein binds phosphatidylinositol-4,5-bisphosphate [PI(4,5)P₂] at the inner leaflet of the plasma membrane and undergoes autonomous translocation across the lipid bilayer (Lolicato et al, 2022). The molecular details of these vesicle-independent UcPS pathways have been extensively reviewed elsewhere (Bai et al, 2025a; Dimou and Nickel, 2018; Pallotta and Nickel, 2020; Sparn et al, 2022; Wang et al, 2022).

The second category encompasses vesicle-dependent unconventional secretion pathways, including secretion mediated by secretory autophagosomes, secretory lysosomes, and multivesicular bodies (MVBs) (Zhang and Schekman, 2013). In these pathways, signal peptide-deficient proteins are packaged into membrane-bound carriers that ultimately fuse with the plasma membrane to release their cargo (Rabouille, 2017). Despite significant progress, two fundamental questions remain unresolved: how unconventional cargo proteins are selectively recognized and loaded into vesicular carriers, and the site within the cell where vesicle-dependent unconventional secretion is initiated.

### UcPS translocators at the ERGIC

Recent mechanistic studies of IL-1β secretion have uncovered a noncanonical pathway for cargo entry into vesicular carriers that is fundamentally distinct from classical endocytosis. This pathway involves protein unfolding followed by direct transmembrane translocation into intracellular membrane compartments, implying the existence of a SEC61-like protein-conducting machinery specialized for unconventional secretion (Zhang et al, 2015). IL-1β secretion is initiated by its encapsulation within a precursor membrane structure that subsequently matures into a secretory autophagosome, defining a vesicle-dependent unconventional protein secretion route (Zhang et al, 2015).

**Figure 3:**
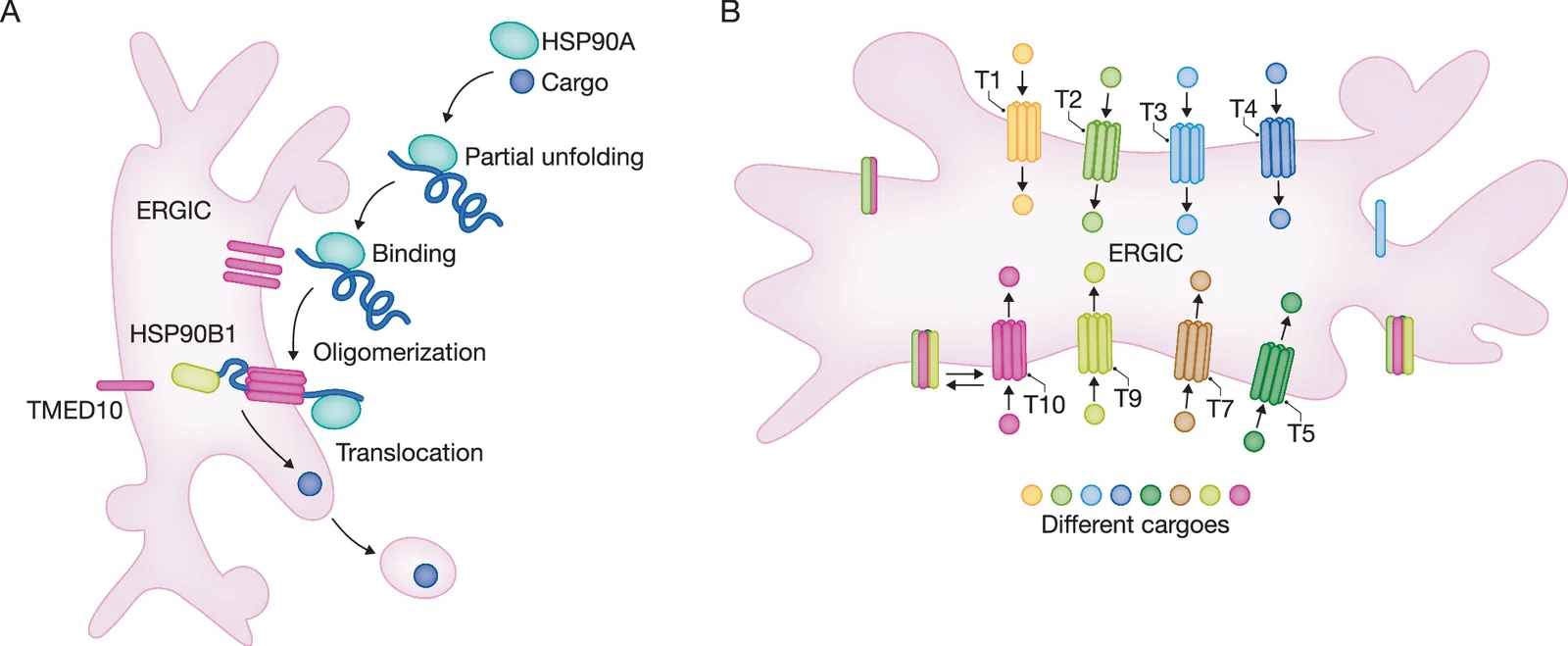
TMEDs-dependent unconventional protein translocation at the ERGIC. (**A**) In the cytosol, unconventional secretory cargo proteins undergo partial unfolding assisted by HSP90A and subsequently bind to TMED10, thereby triggering TMED10 oligomerization and formation of a protein-conducting translocator. With the assistance of HSP90B1, cargo proteins are translocated through the TMED10 translocator into the ERGIC lumen. (**B**) A schematic overview of TMEDs-mediated selective UcPS cargo translocation at the ERGIC to accommodate cargo diversity. Homo-oligomeric TMED assemblies function as active translocators, whereas hetero-oligomeric or monomeric forms are proposed to be non-productive or suppressive for UcPS. However, the hetero-oligomeric TMED can act as cargo receptors for conventional trafficking.

An advance in this field was the identification of TMED10, an ERGIC–resident transmembrane protein, as a central mediator of IL-1β secretion. TMED10 is required for the release of a broad spectrum of unconventional secretory cargoes, including IL-1 family cytokines, galectins, annexin A1, HSPB5, and Tau. Biochemical reconstitution and structural analyses demonstrated that oligomerized TMED10 assembles into a dedicated protein translocator on ERGIC membranes, enabling the translocation of cytosolic cargoes into the ERGIC lumen. In this process, cytosolic HSP90A promotes cargo unfolding, while ERGIC-localized HSP90B1 cooperates with TMED10 to facilitate membrane translocation. This previously unrecognized pathway has been termed TMED10-channeled UcPS (THU) (Zhang et al, 2020b) (Fig. 3A).

Pathologically, the THU pathway can be exploited by viral pathogens. Envelope proteins from highly pathogenic coronaviruses directly enhance TMED10 oligomerization, thereby promoting excessive release of IL-1 family cytokines and driving hyperinflammatory responses (Liu et al, 2024). These findings provide a mechanistic link between viral infection and dysregulated unconventional cytokine secretion.

Beyond inflammation, THU fulfills essential physiological functions in tissue homeostasis. In the intestinal epithelium, TMED10-mediated secretion of IL-33 regulates secretory lineage differentiation, maintains mucus barrier integrity, and confers resistance to colitis, revealing a developmental role for UcPS-derived cytokines (Wang et al, 2024). In the central nervous system, astrocyte-derived IL-33 is actively secreted through TMED10 translocators to suppress astrocyte overactivation and preserve synaptic and neuroimmune homeostasis (Jiao et al, 2024). Extending the functional scope of THU beyond cytokines, the tumor suppressor PTEN is secreted via TMED10 translocators to reprogram tumor-associated macrophages and enhance antitumor immunity, highlighting a cytokine-like role for noncanonically secreted enzymes (Zhang et al, 2024). A new work also suggested a sphingomyelin-activated regulation of TMED10-mediated UcPS of IL-1b in metabolic inflammation (Zheng et al, 2026b).

TMED10 is one member of a nine-protein TMED family (TMED1–7, TMED9, and TMED10), most of which display intrinsic protein translocation activity in vitro, suggesting that multiple TMED proteins contribute to unconventional secretion. Cargo selectivity is partially encoded by sequence features within the cytosolic domains of these proteins. Like TMED10, other TMED family members function at the ERGIC, and this localization is essential for their translocator activity; forced relocalization to the ER abolishes their ability to support UcPS. HSP90 likely cooperates broadly with multiple TMED proteins during cargo translocation. Importantly, TMED proteins act as homo-oligomeric translocators during UcPS, whereas their hetero-oligomeric assemblies—classically involved in conventional secretory cargo transport—suppress unconventional secretion, indicating a functional switch controlled by oligomeric state (Zheng et al, 2026a) (Fig. 3B).

### ERGIC—the “Hub” of UcPS

**Figure 4:**
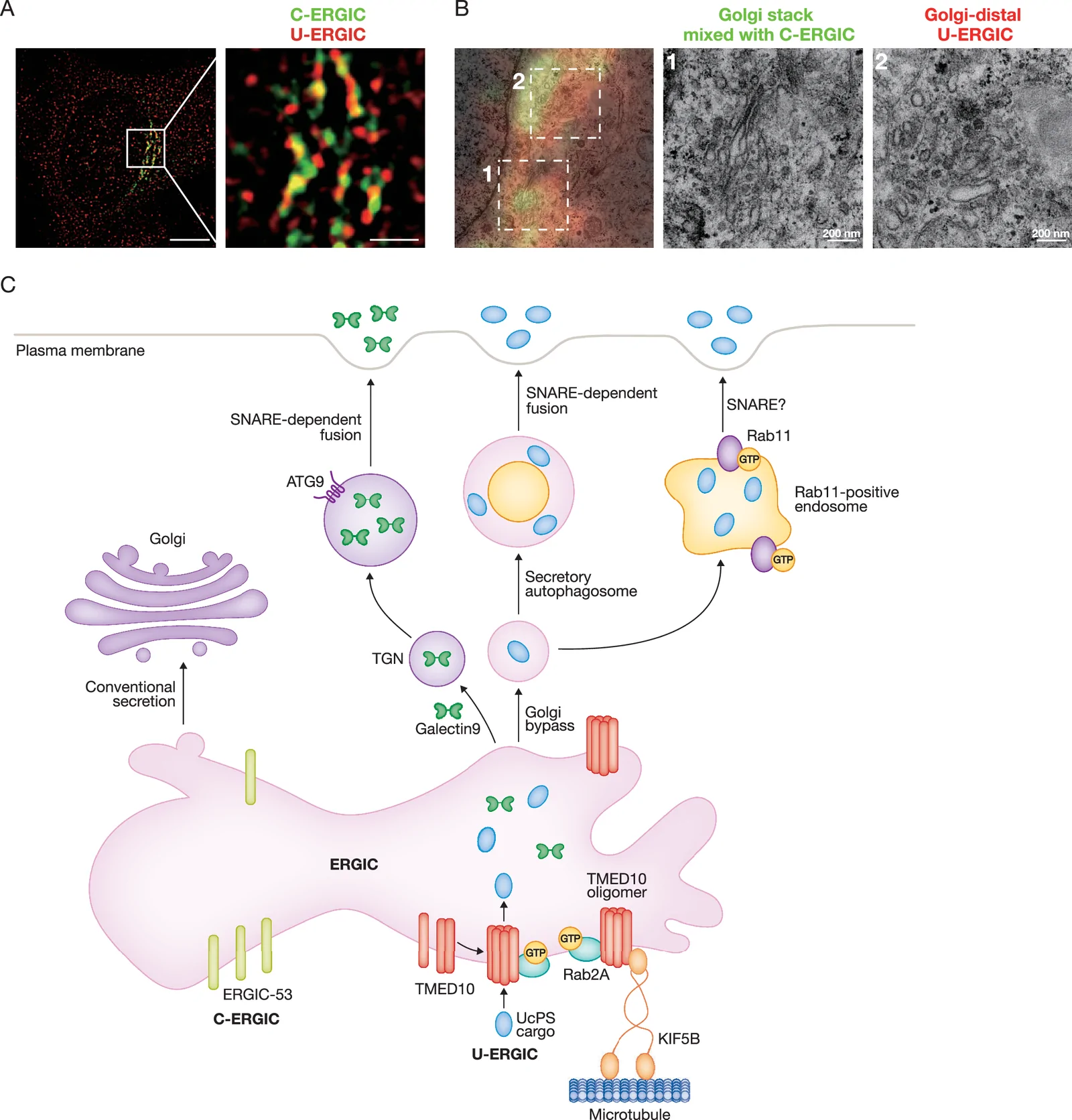
Localization, ultrastructure, and working model of UcPS at the ERGIC. (**A**) Structured illumination microscopy (SIM) imaging showing the distribution of endogenous ERGIC-53 (green) and TMED10 (red) in HeLa cells. ERGIC-53 marks conventional secretory ERGIC domains (C-ERGIC), whereas TMED10 labels unconventional secretion ERGIC domains (U-ERGIC). Right panel shows a magnified view (Zoom) of the boxed region. Scale bars, 5 μm (overview) and 2 μm (inset) (Sun et al, 2024). (**B**) Correlative light and electron microscopy (CLEM) imaging of HeLa cells expressing GFP–ERGIC-53 and mRuby2–TMED10. ERGIC-53-positive structures (region 1) are located in close proximity to the Golgi stack, consistent with conventional Golgi-directed trafficking, whereas TMED10 single-positive regions (region 2) are associated with Golgi-distal tubulovesicular clusters. These structural differences highlight the distinct spatial organization of conventional ERGIC (C-ERGIC) and unconventional ERGIC (U-ERGIC) domains. Scale bar, 200 nm. (**C**) Working model of unconventional protein secretion (UcPS) at the ERGIC. In mammalian cells, the ERGIC is functionally divided into a Golgi-adjacent conventional secretion region (C-ERGIC) and a Golgi-distal unconventional secretion region (U-ERGIC), a process regulated by Rab2A and the motor protein KIF5B along microtubules. Within the U-ERGIC, TMED10 undergoes self-oligomerization to form a translocation machinery that enables cytosolic UcPS cargoes to enter the ERGIC lumen. After entry into the ERGIC lumen, cargoes are exported to the extracellular space via multiple Golgi-bypassing pathways involving recycling endosomes, secretory autophagosomes, or TGN-derived vesicles.

ERGIC and its associated membrane proteins play a pivotal role in vesicle-mediated UcPS. To fulfill this function, the ERGIC must segregate its conventional secretory activities from those dedicated to unconventional secretion. Recent studies have shown that Rab2A, together with the kinesin motor protein KIF5B, functionally partitions the ERGIC into two spatially and functionally distinct domains: a Golgi-proximal, ERGIC-53-positive domain that supports conventional, Golgi-dependent trafficking (referred to as conventional ERGIC, C-ERGIC), and a spatially separated TMED10-enriched subdomain specialized for unconventional secretion (termed unconventional ERGIC, U-ERGIC) (Sun et al, 2024) (Fig. 4A). In this model, the C-ERGIC remains juxtaposed to the Golgi apparatus, enabling efficient Golgi-dependent transport, whereas the U-ERGIC is positioned distal to the Golgi, thereby limiting Golgi access and promoting Golgi-bypass trafficking routes (Sun et al, 2024) (Fig. 4B).

The subsequent fate of ERGIC-derived carriers engaged in Golgi-independent trafficking remains incompletely understood. One possible route involves the trans-Golgi network (TGN). Supporting this idea, recent work demonstrated that the unconventional secretion of Galectin-9 depends on TMED10 and ATG9 and engages TGN-associated trafficking pathways (Zhang et al, 2025c). These observations suggest that, at least for a subset of cargoes, ERGIC-derived unconventional carriers may interface with or transit through the TGN.

Consistent with this concept, nutrient deprivation in *Saccharomyces cerevisiae* induces the formation of specialized tubulovesicular structures near ER exit sites, termed compartments for unconventional protein secretion (CUPS), which are regulated by Grh1, the yeast homolog of mammalian GRASP proteins (Bruns et al, 2011). CUPS exhibit striking structural and functional similarities to the mammalian ERGIC and are thought to serve as initiation sites for vesicle-mediated unconventional secretion in yeast (Curwin et al, 2016). Recent studies have further identified CUPS as COPII- and COPI-independent membrane compartments derived from early Golgi cisternae that cooperate with a modified TGN to collect and sort unconventionally secreted cargo proteins (Curwin et al, 2025). Together, these findings support a model in which multiple noncanonical carriers, including ERGIC- and Golgi-derived intermediates, contribute to UcPS vesicle biogenesis.

Another potential route for Golgi-independent unconventional secretion involves secretory autophagy. As discussed earlier (see the section “Regulation of autophagy by the ERGIC”), the ERGIC plays a critical role in autophagosome biogenesis, raising the possibility that ERGIC-derived COPII vesicles deliver UcPS cargoes directly into nascent autophagosomes (Ge et al, 2013). In this scenario, ERGIC-mediated cargo translocation would be coupled to secretory autophagy, potentially followed by maturation through multivesicular bodies or other endocytic compartments prior to extracellular release. However, the regulatory mechanisms that coordinate the dual fates of autophagosomes—lysosomal degradation versus secretion—remain poorly understood.

Notably, a specialized pericentrosomal ERGIC subdomain marked by Rab1A, termed the pericentrosomal intermediate compartment (pcIC), has been identified in close proximity to the centrosome. This compartment shows intimate association with Rab11-positive endocytic recycling compartments (Marie et al, 2009; Saraste, 2016). Based on these observations, it is tempting to speculate that the pcIC serves as a hub for direct cargo exchange between ERGIC-derived unconventional carriers and endocytic recycling vesicles, thereby integrating secretory and endocytic pathways during UcPS.

Neuronal cells exhibit a conserved trafficking strategy that relies on the ERGIC and directly interfaces with the endocytic recycling system. During nerve growth factor (NGF)-induced differentiation of PC12 cells, ERGIC membranes and ERES become highly enriched at synaptic terminals, whereas the Golgi apparatus and COPI components remain largely confined to the cell body (Sannerud et al, 2006). This striking spatial segregation suggests the presence of a specialized transport pathway that bypasses the canonical Golgi route.

Consistent with this notion, hundreds of neuronal surface proteins—including neurotransmitter receptors, cell adhesion molecules, and voltage-gated ion channels—are delivered to the plasma membrane in a manner that is resistant to brefeldin A (BFA), a classical inhibitor of ER-to-Golgi trafficking. Although these proteins typically appear in highly glycosylated forms, their BFA insensitivity implies the existence of Golgi-independent or noncanonical processing routes (Hanus et al, 2016). Notably, many neuronal dendrites lack conventional Golgi stacks but are enriched in ERGIC membranes and endocytic recycling compartments that exhibit close spatial and functional association (Bowen et al, 2017; Hanus et al, 2014).

A representative example is the delivery of the AMPA-type glutamate receptor subunit GluA1 to dendritic membranes. This process does not require the Golgi apparatus but instead depends on coordinated trafficking between the ERGIC and endocytic recycling compartments (Bowen et al, 2017). Similarly, in dendritic immune cells, the ERGIC-enriched SNARE protein SEC22B interacts with syntaxin 4 (STX4) on phagosomes to mediate partial membrane fusion, a mechanism that plays a critical role in antigen cross-presentation (Cebrian et al, 2011).

Together, these findings support a model in which the ERGIC functions as a regulatory node for UcPS by directly exchanging membrane and cargo with the TGN, autophagic, and endolysosomal system (Fig. 4C). Parallel observations in *Saccharomyces cerevisiae* further reinforce this view, pointing to an evolutionarily conserved role for ERGIC-like compartments as central trafficking hubs that integrate secretory, endocytic, and autophagic pathways. Despite these advances, the molecular mechanisms by which the ERGIC coordinates its diverse roles in Golgi trafficking, unconventional secretion, and autophagy remain incompletely understood and warrant further investigation.

## Regulation of STING activation at the ERGIC

The cyclic GMP–AMP synthase (cGAS)-stimulator of interferon genes (STING) pathway serves as a major cytosolic DNA-sensing system in innate immunity, where cGAS detects aberrant DNA and generates cyclic GMP–AMP (cGAMP) to activate STING (Sun et al, 2013; Wu et al, 2013). Accumulating evidence positions the ERGIC as a central platform for STING activation, signaling diversification, and signal termination. In resting cells, STING resides in the ER, and its exit from the ER constitutes the rate-limiting step for downstream signaling (Dobbs et al, 2015). Early studies demonstrated that STING translocation from the ER to the ERGIC and Golgi is essential for both innate immune activation and autophagy induction, whereas disease-associated STING mutations drive constitutive ER exit and aberrant signaling (Dobbs et al, 2015; Gui et al, 2019).

**Figure 5:**
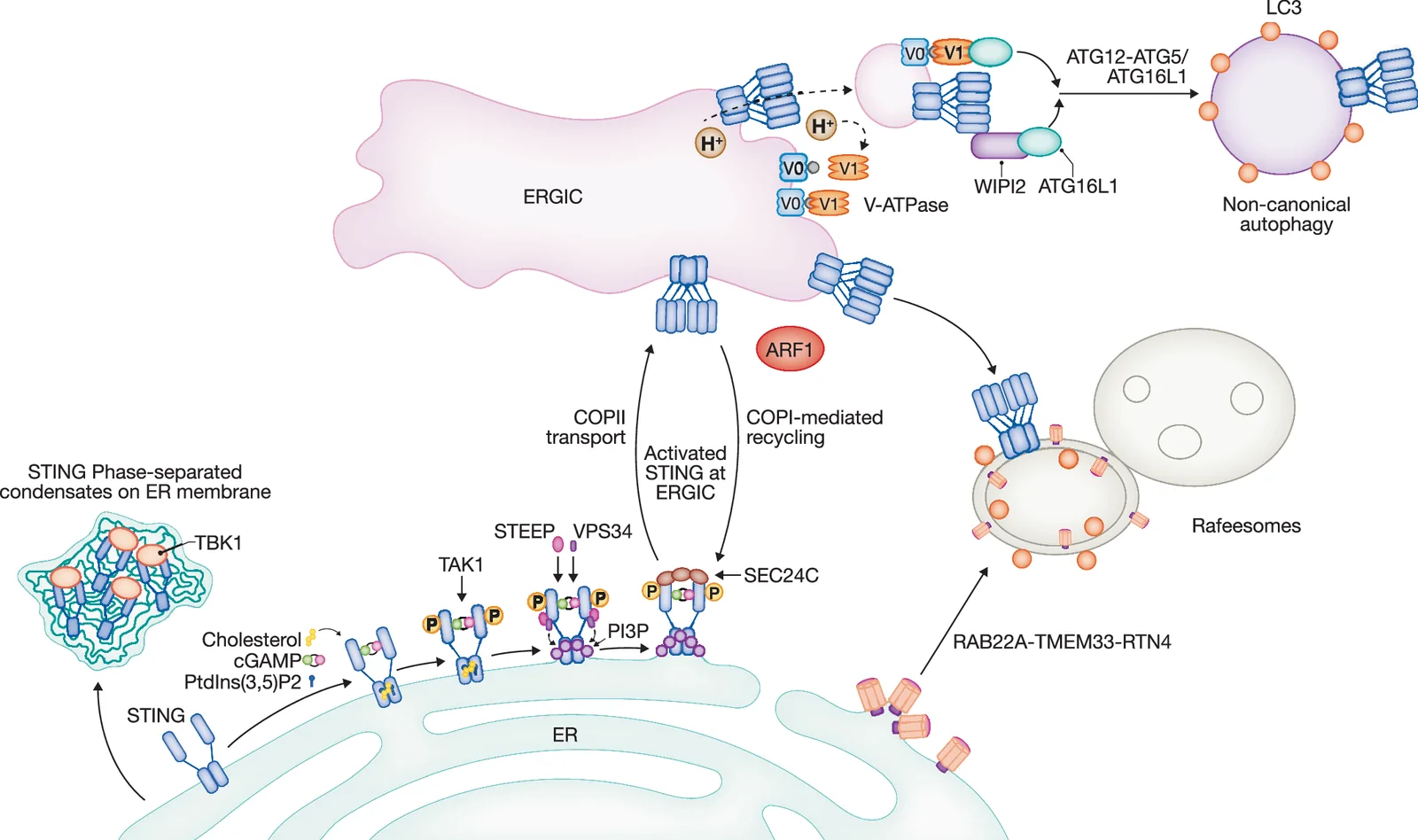
ERGIC as a central hub for STING activation, trafficking, and autophagy. Upon activation at the ER, STING undergoes oligomerization driven by cGAMP and lipid cofactors, including PtdIns(3,5)P₂ and cholesterol, and is exported via COPII-dependent trafficking involving SEC24C, STEEP, and the checkpoint kinase TAK1, with STEEP promoting VPS34-dependent PI3P production and membrane remodeling. At the ERGIC, STING serves as a signaling platform to drive noncanonical autophagy. Mechanistically, STING promotes V-ATPase assembly and recruits WIPI2 and ATG16L1, facilitating LC3 lipidation on single membranes. In parallel, ER/ERGIC-derived membranes contribute to rafeesome formation via the Rab22A–TMEM33–RTN4 complex. STING signaling is further constrained by ARF1-mediated COPI recycling and by the formation of phase-separated STING condensates anchored to the ER membrane, which sequester TBK1 and retain STING to buffer excessive innate immune signaling.

Multiple regulatory layers converge to control STING ER exit and ERGIC targeting. Recent work has identified an evolutionarily conserved EEΦxΦ motif in STING that is recognized by the COPII cargo adapter SEC24C, providing a direct molecular basis for its ER export (Lyu et al, 2026). In addition to protein-based regulatory cues, lipid signals have emerged as critical co-regulators of STING ER exit and oligomerization. Phosphatidylinositol 3,5-bisphosphate (PtdIns(3,5)P₂), generated by the phosphoinositide kinase PIKfyve, has been identified as an endogenous lipid ligand of STING that cooperates with the STING agonist 2’3’-cyclic guanosine monophosphate-adenosine monophosphate (2’3’-cGAMP) to promote its oligomerization and ER exit. Structural analyses further reveal that PtdIns(3,5)P₂ and cholesterol bind at the inter-dimer interface of STING, directly stabilizing higher-order assemblies, thereby providing a mechanistic and structural basis for lipid-dependent STING activation (Li et al, 2026; Tan et al, 2026). The STING ER exit protein (STEEP) promotes VPS34-complex1-mediated PI3P production and ER membrane curvature, thereby facilitating COPII-dependent STING trafficking to the ERGIC (Zhang et al, 2020a). More recently, TAK1 was identified as a key checkpoint kinase that phosphorylates STING at Ser355, enabling its interaction with STEEP, oligomerization, and subsequent translocation to the ERGIC for activation (Ma et al, 2023) (Fig. 5). In parallel, COPII- and COPI-mediated trafficking dynamically balance STING localization: TBK1-dependent phosphorylation events selectively favor COPII-driven ER export of activated STING toward the ERGIC and cis-Golgi, while opposing pathways restrain excessive signaling (Nan et al, 2025).

Beyond acting as a signaling hub, the ERGIC also serves as a membrane and organizational platform for STING-induced autophagy. Upon activation, STING-containing ERGIC membranes directly support LC3 lipidation and autophagosome formation through a noncanonical pathway that depends on WIPI2 and ATG5 but bypasses the ULK1 and VPS34 complexes (Gui et al, 2019). Mechanistically, STING oligomerizes and forms a proton-conducting pore on ERGIC/Golgi-derived vesicles, driving local deacidification (Liu et al, 2023; Xun et al, 2024). This pH change promotes V0–V1 assembly of the V-ATPase, which recruits ATG16L1 via its WD40 domain to STING-positive single membranes (Fischer et al, 2020; Hooper et al, 2022). In parallel, STING directly recruits WIPI2 to these vesicles (Wan et al, 2023) and WIPI2 itself binds ATG16L1 (Dooley et al, 2014), providing a second convergent input onto ATG16L1. Together, both the V-ATPase–ATG16L1 and WIPI2–ATG16L1 axes concentrate the ATG12–ATG5/ATG16L1 E3-like complex at STING-positive membranes, catalyzing LC3 lipidation on a single membrane consistent with the conjugation of ATG8 to single membranes (CASM) model of noncanonical autophagy (Durgan and Florey, 2022; Huang et al, 2025a; Xun et al, 2024) (Fig. 5). In addition, the ER-localized Ca2^+^ pump ATP2A2/SERCA2 regulates STING polymerization and trafficking while licensing SEC62-dependent reticulophagy, thereby coupling ERGIC-localized STING signaling to selective ER turnover (Yang et al, 2025).

The ERGIC further integrates STING signaling with lysosomal degradation and unconventional trafficking routes. Activated STING can escort aberrant or non-self cargoes to lysosomes, exemplified by STING-mediated sorting of LINE-1 ORF1p to Rab7-positive lysosomes via the ERGIC and Golgi, independently of interferon signaling (Huang et al, 2025b). Moreover, activated STING can be packaged into RAB22A-dependent rafeesomes—hybrid organelles formed by fusion of noncanonical autophagosomes with early endosomes, enabling extracellular STING release and propagation of antitumor immunity (Gao et al, 2022; Han et al, 2023b). Recent work shows that rafeesome biogenesis is initiated by a secretory ER-phagy pathway driven by the Rab22A–TMEM33–RTN4 complex, which remodels ER membranes to generate RTN4-positive noncanonical autophagosomes prior to endosomal fusion (Fig. 5) (Zheng et al, 2025). These findings highlight ER/ERGIC-derived membranes as upstream platforms linking STING activation to noncanonical autophagy and unconventional secretion.

Termination and spatial confinement of STING signaling are likewise coordinated through ERGIC-centered trafficking. The small GTPase ARF1 promotes retrograde transport of STING from the ERGIC and Golgi back to the ER, thereby preventing sustained type I interferon signaling. Disease-associated ARF1 mutations disrupt this recycling process, resulting in pathological STING accumulation at the ERGIC/Golgi (Hirschenberger et al, 2023). Jiang and colleagues revealed that STING can form membranous biocondensates, termed STING phase-separators, at the ER in DNA virus-infected or 2′3′-cGAMP-treated cells. These phase-separators, which require STING transmembrane domains, an intrinsically disordered region, and a dimerization domain, constrain STING and TBK1 within ER membranes to prevent overactivation of innate immune signaling. Impairment of STING condensation, as in STING-E336G/E337G mutants, leads to enhanced type I interferon responses, highlighting a spatial and biophysical mechanism for restraining STING signaling beyond trafficking-based regulation (Yu et al, 2021) (Fig. 5).

Collectively, these studies establish the ERGIC as a spatiotemporal control center that integrates STING trafficking, activation, autophagy induction, cargo degradation, and signal resolution. Rather than serving as a passive transit compartment, the ERGIC actively shapes the amplitude, duration, and functional output of STING signaling across innate immunity, autophagy, and disease.

## Viruses assembly and budding at the ERGIC

Emerging evidence has identified the ERGIC as a pivotal organelle for a broad range of enveloped viruses, including members of the *Coronaviridae*, *Poxviridae*, *Arteriviridae*, *Arenaviridae*, *Bunyaviridae*, *Filoviridae*, and *Orthomyxoviridae* families. ERGIC provides specialized membrane structures for virion assembly and budding and mediates the trafficking of viral structural proteins. Different viral families exploit the ERGIC through distinct molecular mechanisms, with coronaviruses being the best-characterized group that specifically assembles at this compartment, followed by some other enveloped viruses (Table 1).

**Table 1:** Summary of ERGIC’s role in virus assembly and budding.

Viral family	Representative viruses	Function of ERGIC	Key host factors
*Coronaviridae*	SARS-CoV-2, SARS-CoV, MERS-CoV, MHV, TGEV, IBV	Budding and assembly platform, unconventional secretion	ARF1, ERGIC-53
*Poxviridae*	Vaccinia Virus	Assembly platform	Vimentin
*Arteriviridae*	PRRSV	Assembly platform	
*Bunyaviridae*	Hantavirus	Replication and assembly platform	Dynein, ERGIC-53
*Arenaviridae*	Junin virus, Lassa virus	Glycoprotein trafficking	ERGIC-53
*Filoviridae*	Ebola virus, Marburg virus	Glycoprotein trafficking	ERGIC-53
*Orthomyxoviridae*	Influenza virus	Glycoprotein trafficking	ERGIC-53

### Coronavirus assembly and budding at the ERGIC

C*oronaviruses* (e.g., SARS-CoV-2, SARS-CoV, MERS-CoV, mouse hepatitis virus [MHV], transmissible gastroenteritis virus [TGEV], Infectious bronchitis virus [IBV]) are the primary viral family identified to specifically assemble at the ERGIC. As discussed in the section ”Establishment of the concept of ER–Golgi Intermediate Compartment”, early studies of MHV-infected cells demonstrated that virion assembly is intracellular and associated with secretory organelles, particularly the ER and Golgi apparatus (Sturman and Holmes, 1983). Subsequent electron microscopy identified viral budding at tubulovesicular ER–Golgi interface membranes—later defined as ERGIC—marked by ERGIC-53, which is incorporated into virions and critical for infectivity and spread (Klaus et al, 2013; Salanueva et al, 1999; Tooze et al, 1984). Rottier and colleagues confirmed the ERGIC as a conserved assembly site across α-, β-, and γ-coronaviruses (Klumperman et al, 1994). Nevertheless, accumulating evidence indicates that SARS‑CoV‑2 assembly is not restricted to the ERGIC alone, but occurs at both ERGIC and Golgi membranes, forming an ERGIC‑Golgi assembly continuum that supports efficient virion biogenesis (Cortese et al, 2020; Scherer et al, 2022).

**Figure 6:**
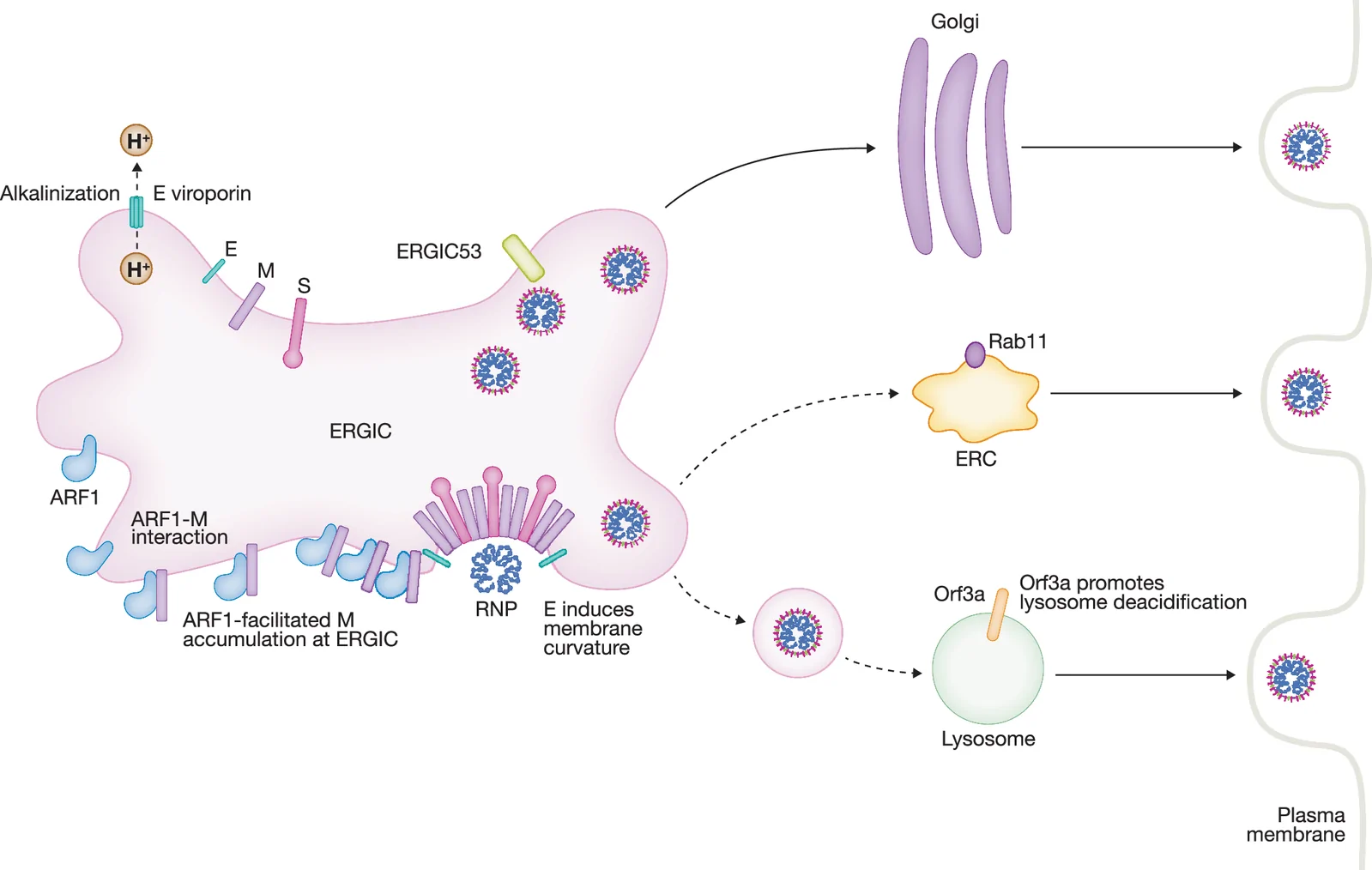
Coronavirus assembly at the ERGIC and egress via distinct pathways. Structural proteins M, E, and S accumulate at the ERGIC, where they assemble around nascent genome-nucleocapsid complexes. Mature virions traverse the Golgi complex or utilize a Golgi-independent pathway (Rab11-positive recycling endosomes or secretory lysosomes) for extracellular secretion. ARF1 promotes M protein accumulation at the ERGIC and orchestrates virion morphogenesis there via molecular interactions with M, E, S, and RNP. The E protein elevates ERGIC pH and induces membrane curvature to support viral assembly and budding. Orf3a protein mediates lysosomal deacidification to facilitate lysosomal exocytosis.

Coronaviral structural proteins, including Spike (S), Membrane (M), and Envelope (E) proteins, are synthesized in the ER and trafficked to the ERGIC via ERES, although the precise molecular mechanisms regulating this trafficking remain incompletely defined(Nal et al, 2005). Recent work identified ARF1 as a key host factor that interacts with SARS-CoV-2 M protein to promote its ERGIC accumulation and efficient assembly—disrupting this interaction impairs viral propagation (Zhang et al, 2025a). Viral genomic encapsidation involves inward budding of N protein-coated RNA at ERGIC membranes, coordinated by viral structural/nonstructural proteins and accessory factors. These viral factors may enhance assembly either by engaging host cargo receptors and trafficking machinery or by reshaping lipid metabolism to generate a membrane environment favorable for budding (Hogue and Machamer, 2007; Sicari et al, 2020). In this regard, the distinctive lipid composition of the ERGIC—particularly its phosphatidylinositol species—has emerged as an important determinant of coronavirus assembly (Cluett et al, 1997) (Fig. 6).

Post-assembly, coronaviruses utilize host secretory pathways for egress. Classical models propose that ERGIC-derived carriers transport virions to the Golgi for conventional secretion, a mechanism supported by the finding that monensin-induced Golgi disruption blocks TGEV release (Salanueva et al, 1999). Consistent with this, BFA strongly inhibits SARS-CoV-2 particle release (Zhang et al, 2025b). However, there is also evidence for Golgi-independent secretion routes, such as the Endocytic Recycling Compartment (ERC) pathway. This route is supported by the colocalization of Infectious Bronchitis Virus (IBV) M protein with Rab11, suggesting a Golgi-bypass interaction between the ERGIC and ERC may facilitate direct viral transfer (Saraste et al, 2022). In addition, a lysosomal exocytosis pathway has been described, whereby viruses like MHV and SARS-CoV-2 utilize the viral protein Orf3a to drive BFA-resistant, de-acidified lysosome-mediated release (Chen et al, 2021; Ghosh et al, 2020)(Fig. 6).

The coexistence of multiple routes likely provides a flexible, context-dependent system for viral release. This complexity, however, raises an unresolved question regarding glycoprotein processing: SARS-CoV-2 S protein requires Golgi-resident enzymes for proper glycosylation (Watanabe et al, 2020). But the SARS-CoV-2 S protein shuffles between the ER and cis-Golgi dependent on coatomer. The controlled dissociation of S from coatomer enables its retention in the ERGIC and virion incorporation (Dey et al, 2023; Dey et al, 2022). One plausible hypothesis is a virus-induced relocalization of Golgi enzymes to ERGIC subdomains via luminal/ lipid environment remodeling (Westerbeck and Machamer, 2019). Notably, SARS-CoV-2 E protein forms a calcium-permeable viroporin in ERGIC membranes, altering ionic milieu to enhance pathogenicity and inflammation (Medeiros-Silva et al, 2023). Another work further showed that the E protein mildly alkalinizes the ERGIC lumen (Wang et al, 2023)—supporting a model where coronaviruses actively remodel ERGIC physiology rather than exploiting it as a passive platform.

### Other virus assembly at ERGIC

Beyond coronaviruses, numerous other enveloped viral families—including *Poxviridae*, *Arteriviridae*, and *Bunyaviridae*—also rely on the ERGIC as a critical platform for their assembly processes.

The *Poxviridae* family, exemplified by Vaccinia Virus (VV), replicates in the cytoplasm but critically depends on the ERGIC as a membrane source and assembly hub. Early electron microscopy and analyses of VV assembly mutants have confirmed that the crescent-shaped membranes of immature virions (IVs) derive from ERGIC-derived compartments. Confocal microscopy further demonstrates the enrichment of ERGIC components at viral assembly sites, where they colocalize with viral core proteins (e.g., p39) and vimentin filaments—these filaments serve as scaffolds for viral factories and facilitate membrane recruitment (Risco et al, 2002). This entire process is coordinated by VV envelope proteins, viral membrane assembly proteins (VMAPs), and host factors. Notably, two ERGIC-localized VV envelope proteins, p21 and p15, are phosphorylated and exert distinct functional roles: p21 orchestrates the organization of recruited viral membranes, while p15 acts as a viral factor that mediates ERGIC-derived membrane recruitment to initiate viral assembly (Weisberg et al, 2017). After ERGIC-dependent assembly, VV matures into intracellular mature virions (IMVs) by acquiring additional membranes from the trans-Golgi or early endosomes, with ESCRT complexes mediating MVB-dependent egress (Huttunen et al, 2021; Sivan et al, 2016).

*Arteriviruses* such as Porcine Reproductive and Respiratory Syndrome Virus (PRRSV) utilize the ER and ERGIC for coordinated assembly of membrane and nucleocapsid components. PRRSV envelope is composed of major envelope proteins (GP5 and M protein) and minor envelope proteins (GP2a, GP3, GP4, E protein, ORF5a) (Wissink et al, 2005). During assembly, the nucleocapsid (N) protein, initially located within the ER/ERGIC, interacts with these envelope proteins (Bai et al, 2025b). This process is facilitated by the viral non-structural protein nsp2, which acts as a molecular magnet to herd structural proteins together, thereby streamlining the formation of new virions (Bai et al, 2025b; Guo et al, 2021).

Hantaviruses, members of the *Bunyaviridae* family, require ERGIC for their replication, assembly, and protein trafficking. Hantaviral nucleocapsid protein N is trafficked to the ERGIC via dynein-mediated microtubule transport and assembles with viral RNA into ribonucleoprotein(RNP) complexes at this compartment (Ramanathan et al, 2007). Viral glycoprotein precursors Gn/Gc achieve ER-to-ERGIC trafficking and initial maturation in an ERGIC-53-dependent manner, before subsequent processing in the Golgi apparatus (Klaus et al, 2013). The integration of viral nucleocapsids and glycoproteins within the ERGIC and early Golgi compartments results in the production of infectious hantaviral particles.

### ERGIC-53 mediates the trafficking of viral proteins

In addition to its role in viral assembly, the ERGIC also mediates the transport of various viral proteins. As a cargo receptor that mediates glycoprotein trafficking within the early exocytic pathway, ERGIC-53 associates with the glycoproteins (GPs) of multiple viral families, including *Arenaviridae* (e.g., Junin virus [JUNV], Lassa virus [LASV]), *Filoviridae* (e.g., Ebola virus [EBOV], Marburg virus [MARV]), *Bunyaviridae* (Hantavirus), *Orthomyxoviridae* (Influenza virus) (Klaus et al, 2013). In the case of arenaviruses, ERGIC-53 binds to their GPs through a lectin-independent mode, translocates to viral budding sites, and becomes incorporated into mature virions. ERGIC-53 is a critical factor for the propagation of arenaviruses, coronaviruses, and filoviruses: its depletion does not abrogate the formation of GP-containing viral particles, but renders these particles noninfectious, in part because they lose the ability to attach to host cells (Klaus et al, 2013).

Despite considerable advances, current models of ERGIC-centered viral assembly rely heavily on electron and fluorescence microscopy observations, which fail to fully capture the dynamic and heterogeneous properties of these subcellular compartments. Key questions thus remain unanswered, including: (1) which ERGIC subdomains specifically support viral assembly; (2) why this compartment is preferentially exploited over other secretory organelles—a bias potentially attributable to its unique role as a trafficking hub, distinct lipid composition that favors membrane curvature, and intrinsic dynamic characteristics; and (3) what host and viral factors govern this spatial specificity. Answering these questions will be critical for mechanistically understanding the biogenesis of diverse enveloped viruses and may also uncover novel host-directed therapeutic targets that disrupt viral assembly and egress.

## ERGIC-like structures under cellular stress

Cells possess a sophisticated protein quality control system that monitors proper protein folding and ensures correct protein localization. The accumulation of misfolded proteins in the ER triggers ER stress, activating a cascade of events collectively termed the unfolded protein response (UPR)(Sun and Brodsky, 2019). The UPR comprises three major signaling pathways mediated by inositol-requiring enzyme 1 alpha (IRE1), protein kinase R (PERK), and activating transcription factor 6 (ATF6). UPR activation restores proteostasis through multiple mechanisms: enhancing ER protein-folding capacity, transiently suppressing global protein synthesis, and accelerating degradation of misfolded proteins (Karagoz et al, 2019). During ER stress in both yeast and mammalian cells, specialized structures called “ER whorls” are induced (Bernales et al, 2006; Xu et al, 2021). In mammalian cells, their formation correlates with PERK kinase activation and requires SAR1 protein and COPII complex activity, which collectively promote the generation of tubular ER whorl precursors. These precursors mature through SEC22B-mediated membrane fusion. Notably, this process appears to limit SEC61 functionality, thereby reducing overall protein synthesis (Xu et al, 2021). ER whorl formation depends on COPII regulation and SNARE protein SEC22B, and their high membrane curvature resembles ERGIC characteristics, suggesting ER whorls may represent a specialized form of ERGIC. In addition to ER whorls, a recently described ER-derived tubulovesicular structure termed the ER tubular body (ER-TB) forms in mammalian cells under conditions of ER stress and ER-to-Golgi transport blockade (Song et al, 2025), providing an alternative trafficking route under secretory stress. ER-TB biogenesis depends on the tubular ER-phagy receptors ATL3 and RTN3L and supports Golgi-independent unconventional secretion of selected transmembrane cargoes. Although molecularly distinct from the ERGIC, ER-TB exhibits ERGIC-like morphological features and functions as a stress-induced trafficking intermediate (Song et al, 2025). Similarly, nutrient-starved yeast cells develop Grh1-regulated tubulovesicular CUPS structures (see the section “ERGIC—the “Hub” of UcPS”). CUPS’ structural, positional, and functional features indicate it may be an ER-derived, stress-induced ERGIC-like compartment.

## Perspectives

**Figure 7:**
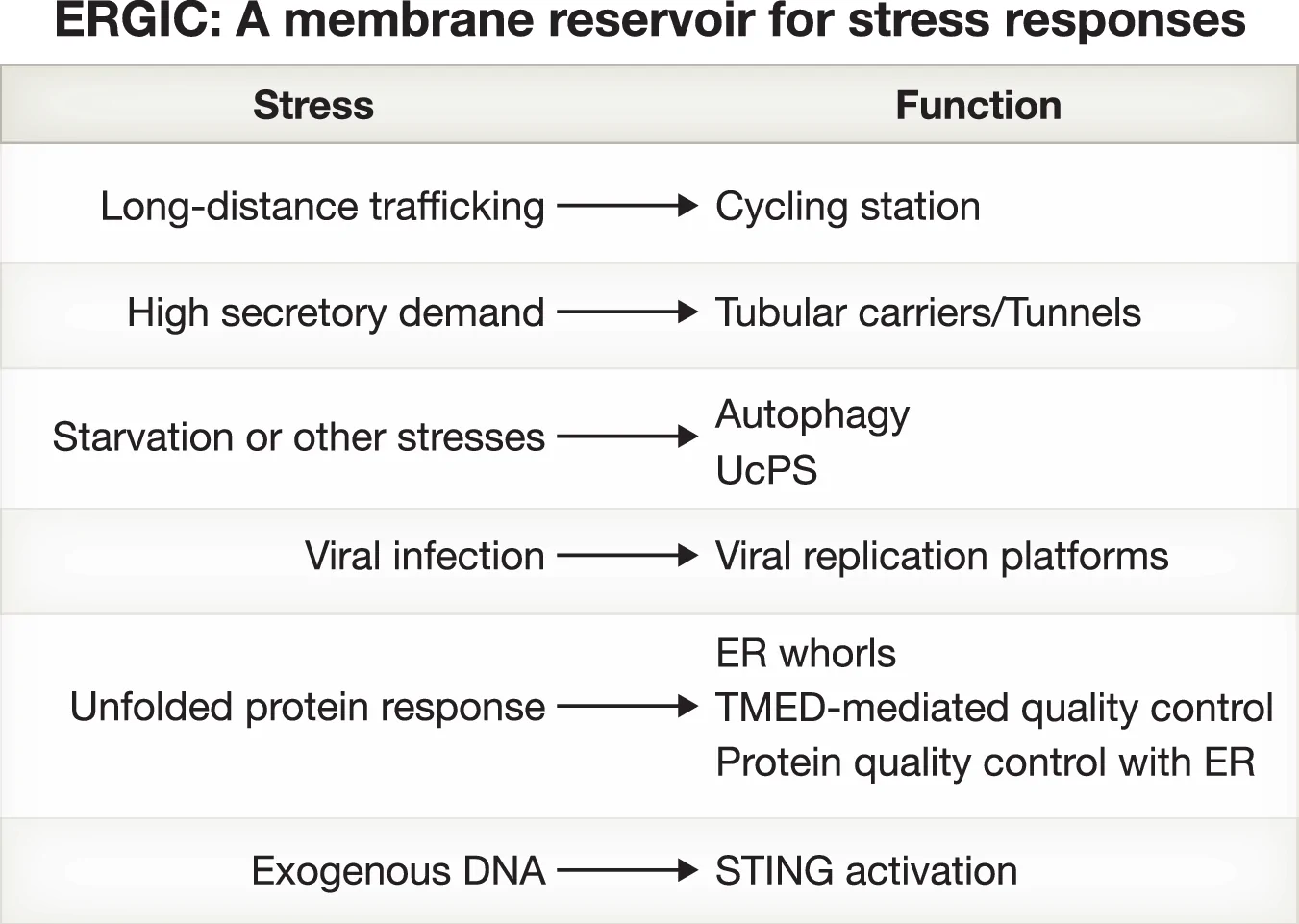
The ERGIC as a membrane reservoir in various cellular stress responses. The ERGIC adapts to diverse cellular stresses by supporting multiple functions, including trafficking, secretion, autophagy, viral replication, protein quality control, and STING-mediated innate immune signaling.

ERGIC is a ubiquitous and highly dynamic component of the endomembrane system that performs diverse biological functions. Under physiological conditions, it primarily coordinates cargo transport between the ER and Golgi. However, during cellular stress, ERGIC assumes more critical roles—such as restoring cellular homeostasis through autophagy and transmitting stress signals via unconventional protein secretion. These multifaceted functions rely on ERGIC’s unique tubulovesicular architecture and its capacity for vesicular trafficking and cargo sorting. Collectively, these properties position ERGIC as a central hub regulating membrane transport and directional cargo flow in both homeostatic and stress conditions, thereby influencing cellular functional states. Given its pivotal role in the endomembrane system, certain pathogens (e.g., coronaviruses) may exploit ERGIC to subvert normal host membrane functions, facilitating their replication and dissemination. Therefore, the ERGIC is not merely a cycling compartment, as assumed two decades ago, but instead represents a dynamic membrane reservoir that is mobilized in response to cellular stress, including high-demand secretion, starvation, and pathogen infection (Fig. 7).

Nevertheless, our understanding of ERGIC’s involvement in cellular processes and its structural dynamics remains limited, and several fundamental questions regarding its organization and functions remain unresolved, particularly regarding how it modulates diverse cellular activities under varying physiological or pathological contexts (see Box 1). The evolution of ERGIC may represent an adaptation to the complex transport demands between the ER and Golgi in mammalian cells, especially given the challenges of long-distance ER–Golgi trafficking and the high demand for secretion. ERGIC is directly linked to multiple stress-responsive processes, including autophagy, unconventional protein secretion, proteostasis, and viral assembly. Under specific stress conditions, ERGIC-like structures—such as CUPS and ER whorls—emerge and play vital roles in maintaining intracellular stability.

In addition, our understanding of the morphological characteristics of ERGIC across different species and the underlying reasons for such heterogeneity remains incomplete. For instance: (i) In non-vertebrate organisms, a stable, morphologically defined ERGIC comparable to that in mammalian cells has long been considered absent. However, accumulating evidence indicates that ERGIC-like or ER-derived tubulovesicular compartments can form under specific physiological or stress conditions, and functional homologs and key proteins (e.g., ERGIC-53, KDEL receptors, and TMED protein family members) are conserved. It remains unclear whether ERGIC exists in alternative forms in non-vertebrate cells or is induced under specific stress conditions. For example, nutrient starvation in yeast triggers the formation of CUPS, a membrane structure implicated in unconventional secretion pathways that may represent a specialized form of ERGIC (Bruns et al, 2011; Curwin et al, 2016). (ii) Although the structure and function of ERGIC in mammalian cells have been extensively studied, knowledge regarding its presence, structural features, and operational mechanisms in other vertebrate cell types remains limited.

Recent advances in optical and electron microscopy technologies have significantly enhanced our understanding of ERGIC’s morphological characteristics, dynamic behaviors, and its functional compartmentalization and regulatory mechanisms under changing cellular conditions. These imaging breakthroughs not only facilitate deeper insights into ERGIC’s multifunctional roles but may also help identify other potential endomembrane structures associated with ERGIC, such as CUPS and ER whorls. When combined with biochemical isolation and omics technologies, these approaches enable more comprehensive and detailed analysis of ERGIC’s molecular composition, which is crucial for elucidating the precise mechanisms underlying its diverse functions. Comparative studies of ERGIC’s structural variations and biological roles across different species will provide valuable evolutionary perspectives on how this compartment has adapted to increasing biological complexity. Looking forward, interdisciplinary approaches integrating mathematical modeling and physiological studies will be essential for investigating ERGIC’s structural and functional regulation under various physiological and pathological conditions. Such research will yield a more complete understanding of ERGIC’s roles in both normal and disease states, potentially providing novel theoretical foundations and therapeutic directions for related human diseases.

Box 1 Outstanding questions in ERGIC biology Although the endoplasmic reticulum–Golgi intermediate compartment (ERGIC) has long been viewed as a trafficking station between the ER and the Golgi, recent studies suggest that it functions as a multifunctional regulatory hub. However, several fundamental questions regarding its organization and functions remain unresolved.

**1. What defines the tubulovesicular architecture and dynamics of the ERGIC?**

The ERGIC has traditionally been defined as a membrane compartment characterized by a distinctive tubulovesicular morphology and highly dynamic behavior. However, the molecular mechanisms that generate and maintain these structural and dynamic properties remain poorly understood. Addressing this question will require high-resolution live-cell imaging, quantitative ultrastructural analyses, and approaches capable of tracking ERGIC membranes over time. These efforts should be combined with genetic or proteomic screening strategies to identify the key factors involved, including membrane-shaping proteins and regulators of membrane remodeling.

**2. How does the ERGIC accommodate multiple cellular functions?**

In addition to its classical role in cargo trafficking between the endoplasmic reticulum and the Golgi apparatus, the ERGIC has emerged as a multifunctional platform involved in diverse processes, including autophagosome formation and unconventional protein secretion. Although evidence suggests that functional subcompartments may exist within the ERGIC, it remains unclear how these distinct activities are spatially and temporally coordinated. Furthermore, the upstream signals that determine which function is engaged under specific cellular conditions remain largely unknown. Super-resolution imaging combined with systematic signaling and genetic screens will be instrumental in resolving these questions.

**3. How does protein translocation occur at the ERGIC during unconventional protein secretion?**

The ERGIC has recently been identified as a key platform for unconventional protein secretion. However, the mechanisms by which cytosolic proteins gain access to ERGIC membranes remain largely unresolved. Elucidating this process will require structural dissection of the translocation machinery as well as biochemical and functional reconstitution of the translocation complex to determine how cytosolic cargoes cross or integrate into ERGIC membranes.

**4. How does the ERGIC mediate Golgi-bypass trafficking?**

Another emerging role of the ERGIC is in mediating Golgi-bypass trafficking pathways. Despite this functional assignment, the downstream compartments involved in this route remain poorly defined. Understanding this pathway will require live-cell imaging of ERGIC membrane dynamics together with comprehensive mapping of interacting organelles and membrane compartments, allowing the reconstruction of their dynamic interactions and trafficking routes.

**5. What is the fundamental nature of the ERGIC?**

Recent studies suggest that the ERGIC may represent a dynamic membrane reservoir that becomes particularly important during cellular stress, facilitating the formation of specialized compartments involved in endomembrane remodeling. This raises several fundamental questions: why does the ER–Golgi system maintain such a membrane pool, and how is it mobilized under stress conditions? Moreover, the ERGIC may possess additional, yet-unrecognized functions. A comprehensive analysis of ERGIC structure and function across diverse physiological and pathological contexts will therefore be essential for understanding its broader roles in cell biology.

## Supplementary information

Peer Review File

## References

[CR1] Aber R, Chan W, Mugisha S, Jerome-Majewska LA (2019) Transmembrane emp24 domain proteins in development and disease. Genet Res 101:e14 10.1017/S0016672319000090 PMC7045115 31878985

[CR2] Antonny B, Madden D, Hamamoto S, Orci L, Schekman R (2001) Dynamics of the COPII coat with GTP and stable analogues. Nat Cell Biol 3:531–537 10.1038/35078500 11389436

[CR3] Appenzeller C, Andersson H, Kappeler F, Hauri HP (1999) The lectin ERGIC-53 is a cargo transport receptor for glycoproteins. Nat Cell Biol 1:330–334 10.1038/14020 10559958

[CR4] Appenzeller-Herzog C, Hauri HP (2006) The ER-Golgi intermediate compartment (ERGIC): in search of its identity and function. J Cell Sci 119:2173–2183 10.1242/jcs.03019 16723730

[CR5] Appenzeller-Herzog C, Roche AC, Nufer O, Hauri HP (2004) pH-induced conversion of the transport lectin ERGIC-53 triggers glycoprotein release. J Biol Chem 279:12943–12950 10.1074/jbc.M313245200 14718532

[CR6] Aridor M (2026) Beyond vesicular traffic: the ERES as the gateway for biosynthetic secretion. Subcell Biochem 111:3–36 10.1007/978-3-032-16833-7_1 41718970

[CR7] Aridor M, Bannykh SI, Rowe T, Balch WE (1995) Sequential coupling between COPII and COPI vesicle coats in endoplasmic reticulum to Golgi transport. J Cell Biol 131:875–893 10.1083/jcb.131.4.875 PMC2200014 7490291

[CR8] Bai Y, Pan Y, Liu X (2025a) Mechanistic insights into gasdermin-mediated pyroptosis. Nat Rev Mol Cell Biol 26:501–521 10.1038/s41580-025-00837-0 40128620

[CR9] Bai YZ, Wang S, Sun Y, Liu YG, Zhang HL, Wang Q, Huang R, Rao CH, Xu SJ, Tian ZJ et al (2025b) The full-length nsp2 replicase contributes to viral assembly in highly pathogenic PRRSV-2. J Virol 99:e0182124 10.1128/jvi.01821-24 PMC11784222 39601570

[CR10] Bannykh SI, Rowe T, Balch WE (1996) The organization of endoplasmic reticulum export complexes. J Cell Biol 135:19–35 10.1083/jcb.135.1.19 PMC2121027 8858160

[CR11] Barlowe C, Orci L, Yeung T, Hosobuchi M, Hamamoto S, Salama N, Rexach MF, Ravazzola M, Amherdt M, Schekman R (1994) COPII: a membrane coat formed by Sec proteins that drive vesicle budding from the endoplasmic reticulum. Cell 77:895–907 10.1016/0092-8674(94)90138-4 8004676

[CR12] Bazua-Valenti S, Brown MR, Zavras J, Riedl Khursigara M, Grinkevich E, Sidhom EH, Keller KH, Racette M, Dvela-Levitt M, Quintanova C et al (2024) Disrupted uromodulin trafficking is rescued by targeting TMED cargo receptors. J Clin Invest 134(24):e180347 10.1172/JCI180347 PMC11645142 39680459

[CR13] Beck R, Rawet M, Wieland FT, Cassel D (2009) The COPI system: molecular mechanisms and function. FEBS Lett 583:2701–2709 10.1016/j.febslet.2009.07.032 19631211

[CR14] Ben-Tekaya H, Kahn RA, Hauri HP (2010) ADP ribosylation factors 1 and 4 and group VIA phospholipase A(2) regulate morphology and intraorganellar traffic in the endoplasmic reticulum-Golgi intermediate compartment. Mol Biol Cell 21:4130–4140 10.1091/mbc.E10-01-0022 PMC2993742 20881058

[CR15] Ben-Tekaya H, Miura K, Pepperkok R, Hauri HP (2005) Live imaging of bidirectional traffic from the ERGIC. J Cell Sci 118:357–367 10.1242/jcs.01615 15632110

[CR16] Bernales S, McDonald KL, Walter P (2006) Autophagy counterbalances endoplasmic reticulum expansion during the unfolded protein response. PLoS Biol 4:e423 10.1371/journal.pbio.0040423 PMC1661684 17132049

[CR17] Bethune J, Wieland FT (2018) Assembly of COPI and COPII vesicular coat proteins on membranes. Annu Rev Biophys 47:63–83 10.1146/annurev-biophys-070317-033259 29345989

[CR18] Bowen AB, Bourke AM, Hiester BG, Hanus C, Kennedy MJ (2017) Golgi-independent secretory trafficking through recycling endosomes in neuronal dendrites and spines. eLife 6:e27362 10.7554/eLife.27362 PMC5624785 28875935

[CR19] Brandizzi F, Barlowe C (2013) Organization of the ER-Golgi interface for membrane traffic control. Nat Rev Mol Cell Biol 14:382–392 10.1038/nrm3588 PMC4064004 23698585

[CR20] Breuza L, Halbeisen R, Jeno P, Otte S, Barlowe C, Hong W, Hauri HP (2004) Proteomics of endoplasmic reticulum-Golgi intermediate compartment (ERGIC) membranes from brefeldin A-treated HepG2 cells identifies ERGIC-32, a new cycling protein that interacts with human Erv46. J Biol Chem 279:47242–47253 10.1074/jbc.M406644200 15308636

[CR21] Brier LW, Zhang M, Ge L (2016) Mechanistically dissecting autophagy: insights from in vitro reconstitution. J Mol Biol 428:1700–1713 10.1016/j.jmb.2016.02.024 PMC5599101 26946034

[CR22] Bruns C, McCaffery JM, Curwin AJ, Duran JM, Malhotra V (2011) Biogenesis of a novel compartment for autophagosome-mediated unconventional protein secretion. J Cell Biol 195:979–992 10.1083/jcb.201106098 PMC3241719 22144692

[CR23] Budnik A, Stephens DJ (2009) ER exit sites-localization and control of COPII vesicle formation. FEBS Lett 583:3796–3803 10.1016/j.febslet.2009.10.038 19850039

[CR24] Cabrera M, Muniz M, Hidalgo J, Vega L, Martin ME, Velasco A (2003) The retrieval function of the KDEL receptor requires PKA phosphorylation of its C-terminus. Mol Biol Cell 14:4114–4125 10.1091/mbc.E03-04-0194 PMC207004 14517323

[CR25] Cai H, Yu S, Menon S, Cai Y, Lazarova D, Fu C, Reinisch K, Hay JC, Ferro-Novick S (2007) TRAPPI tethers COPII vesicles by binding the coat subunit Sec23. Nature 445:941–944 10.1038/nature05527 17287728

[CR26] Cancino J, Capalbo A, Di Campli A, Giannotta M, Rizzo R, Jung JE, Di Martino R, Persico M, Heinklein P, Sallese M et al (2014) Control systems of membrane transport at the interface between the endoplasmic reticulum and the Golgi. Dev Cell 30:280–294 10.1016/j.devcel.2014.06.018 25117681

[CR27] Cebrian I, Visentin G, Blanchard N, Jouve M, Bobard A, Moita C, Enninga J, Moita LF, Amigorena S, Savina A (2011) Sec22b regulates phagosomal maturation and antigen crosspresentation by dendritic cells. Cell 147:1355–1368 10.1016/j.cell.2011.11.021 22153078

[CR28] Centonze FG, Reiterer V, Nalbach K, Saito K, Pawlowski K, Behrends C, Farhan H (2019) LTK is an ER-resident receptor tyrosine kinase that regulates secretion. J Cell Biol 218:2470–2480 10.1083/jcb.201903068 PMC6683734 31227593

[CR29] Chen D, Zheng Q, Sun L, Ji M, Li Y, Deng H, Zhang H (2021) ORF3a of SARS-CoV-2 promotes lysosomal exocytosis-mediated viral egress. Dev Cell 56:3250–3263.e3255 10.1016/j.devcel.2021.10.006 PMC8502680 34706264

[CR30] Chen RH, Costa-Filho AJ, Debnath J, Galli T, Ge L, Goberdhan D, Guo W, He K, Jacob R, Kang T et al (2025) Beyond the secretory pathway: new insights into protein release. Traffic 26:e70022 10.1111/tra.70022 PMC12590341 41195561

[CR31] Chia J, Wang SC, Wee S, Gill DJ, Tay F, Kannan S, Verma CS, Gunaratne J, Bard FA (2021) Src activates retrograde membrane traffic through phosphorylation of GBF1. eLife 10:e68678 10.7554/eLife.68678 PMC8727025 34870592

[CR32] Claude A (1946) Fractionation of mammalian liver cells by differential centrifugation; experimental procedures and results. J Exp Med 84:61–89 20988751

[CR33] Claude A (1970) Growth and differentiation of cytoplasmic membranes in the course of lipoprotein granule synthesis in the hepatic cell. I. Elaboration of elements of the Golgi complex. J Cell Biol 47:745–766 10.1083/jcb.47.3.745 PMC2108163 5497550

[CR34] Claude A, Fullam EF (1946) The preparation of sections of guinea pig liver for electron microscopy. J Exp Med 83:499–503 PMC2135593 19871546

[CR35] Claude A, Zhao BP, Kuziemsky CE, Dahan S, Berger SJ, Yan JP, Armold AD, Sullivan EM, Melancon P (1999) GBF1: a novel Golgi-associated BFA-resistant guanine nucleotide exchange factor that displays specificity for ADP-ribosylation factor 5. J Cell Biol 146:71–84 PMC2199737 10402461

[CR36] Cluett EB, Kuismanen E, Machamer CE (1997) Heterogeneous distribution of the unusual phospholipid semilysobisphosphatidic acid through the Golgi complex. Mol Biol Cell 8:2233–2240 10.1091/mbc.8.11.2233 PMC25704 9362065

[CR37] Contreras FX, Ernst AM, Haberkant P, Bjorkholm P, Lindahl E, Gonen B, Tischer C, Elofsson A, von Heijne G, Thiele C et al (2012) Molecular recognition of a single sphingolipid species by a protein’s transmembrane domain. Nature 481:525–529 10.1038/nature10742 22230960

[CR38] Cortese M, Lee JY, Cerikan B, Neufeldt CJ, Oorschot VMJ, Kohrer S, Hennies J, Schieber NL, Ronchi P, Mizzon G et al (2020) Integrative imaging reveals SARS-CoV-2-induced reshaping of subcellular morphologies. Cell Host Microbe 28:853–866 e855 10.1016/j.chom.2020.11.003 PMC7670925 33245857

[CR39] Cunningham WP, Morre DJ, Mollenhauer HH (1966) Structure of isolated plant Golgi apparatus revealed by negative staining. J Cell Biol 28:169–179 10.1083/jcb.28.2.169 PMC2106932 4161888

[CR40] Curwin AJ, Brouwers N, Alonso YAM, Teis D, Turacchio G, Parashuraman S, Ronchi P, Malhotra V (2016) ESCRT-III drives the final stages of CUPS maturation for unconventional protein secretion. eLife 5:e16299 10.7554/eLife.16299 PMC4868542 27115345

[CR41] Curwin AJ, Kurokawa K, Bigliani G, Brouwers N, Nakano A, Malhotra V (2025) The pathway of unconventional protein secretion involves CUPS and a modified trans-Golgi network. J Cell Biol 224:e202312120 10.1083/jcb.202312120 PMC11867701 40015244

[CR42] Dalton AJ, Felix MD (1954) Cytologic and cytochemical characteristics of the Golgi substance of epithelial cells of the epididymis in situ, in homogenates and after isolation. Am J Anat 94:171–207 10.1002/aja.1000940202 13148119

[CR43] Davis S, Wang J, Zhu M, Stahmer K, Lakshminarayan R, Ghassemian M, Jiang Y, Miller EA, Ferro-Novick S (2016) Sec24 phosphorylation regulates autophagosome abundance during nutrient deprivation. eLife 5:e21167 10.7554/eLife.21167 PMC5148606 27855785

[CR44] Dey D, Qing E, He Y, Chen Y, Jennings B, Cohn W, Singh S, Gakhar L, Schnicker NJ, Pierce BG et al (2023) A single C-terminal residue controls SARS-CoV-2 spike trafficking and incorporation into VLPs. Nat Commun 14:8358 10.1038/s41467-023-44076-3 PMC10724246 38102143

[CR45] Dey D, Singh S, Khan S, Martin M, Schnicker NJ, Gakhar L, Pierce BG, Hasan SS (2022) An extended motif in the SARS-CoV-2 spike modulates binding and release of host coatomer in retrograde trafficking. Commun Biol 5:115 10.1038/s42003-022-03063-y PMC8825798 35136165

[CR46] Dimou E, Nickel W (2018) Unconventional mechanisms of eukaryotic protein secretion. Curr Biol 28:R406–R410 10.1016/j.cub.2017.11.074 29689224

[CR47] Ding J, Wang K, Liu W, She Y, Sun Q, Shi J, Sun H, Wang DC, Shao F (2016) Pore-forming activity and structural autoinhibition of the gasdermin family. Nature 535:111–116 10.1038/nature18590 27281216

[CR48] Ding X, Jiang X, Tian R, Zhao P, Li L, Wang X, Chen S, Zhu Y, Mei M, Bao S et al (2019) RAB2 regulates the formation of autophagosome and autolysosome in mammalian cells. Autophagy 15:1774–1786 10.1080/15548627.2019.1596478 PMC6735470 30957628

[CR49] Dobbs N, Burnaevskiy N, Chen D, Gonugunta VK, Alto NM, Yan N (2015) STING activation by translocation from the ER is associated with infection and autoinflammatory disease. Cell Host Microbe 18:157–168 10.1016/j.chom.2015.07.001 PMC4537353 26235147

[CR50] Dominguez M, Dejgaard K, Fullekrug J, Dahan S, Fazel A, Paccaud JP, Thomas DY, Bergeron JJ, Nilsson T (1998) gp25L/emp24/p24 protein family members of the cis-Golgi network bind both COP I and II coatomer. J Cell Biol 140:751–765 10.1083/jcb.140.4.751 PMC2141742 9472029

[CR51] Dooley HC, Razi M, Polson HE, Girardin SE, Wilson MI, Tooze SA (2014) WIPI2 links LC3 conjugation with PI3P, autophagosome formation, and pathogen clearance by recruiting Atg12-5-16L1. Mol Cell 55:238–252 10.1016/j.molcel.2014.05.021 PMC4104028 24954904

[CR52] D’Souza-Schorey C, Chavrier P (2006) ARF proteins: roles in membrane traffic and beyond. Nat Rev Mol Cell Biol 7:347–358 10.1038/nrm1910 16633337

[CR53] Du Y, Fan X, Song C, Chang W, Xiong J, Deng L, Ji WK (2024) Sec23IP recruits VPS13B/COH1 to ER exit site-Golgi interface for tubular ERGIC formation. J Cell Biol 223:e202402083 10.1083/jcb.202402083 PMC11457499 39352497

[CR54] Durgan J, Florey O (2022) Many roads lead to CASM: diverse stimuli of noncanonical autophagy share a unifying molecular mechanism. Sci Adv 8:eabo1274 10.1126/sciadv.abo1274 PMC9604613 36288315

[CR55] Dvela-Levitt M, Kost-Alimova M, Emani M, Kohnert E, Thompson R, Sidhom EH, Rivadeneira A, Sahakian N, Roignot J, Papagregoriou G et al (2019) Small molecule targets TMED9 and promotes lysosomal degradation to reverse proteinopathy. Cell 178:521–535 e523 10.1016/j.cell.2019.07.002 31348885

[CR56] Evavold CL, Ruan J, Tan Y, Xia S, Wu H, Kagan JC (2018) The pore-forming protein gasdermin D regulates interleukin-1 secretion from living macrophages. Immunity 48:35–44 e36 10.1016/j.immuni.2017.11.013 PMC5773350 29195811

[CR57] Farhan H, Raote I, Campelo F, Ge L, Hirschberg K, Forrester A, Zanetti G, Lippincott-Schwartz J, Pastor-Pareja JC, Perez F et al (2025) Towards a unified framework for the function of endoplasmic reticulum exit sites. Nat Rev Mol Cell Biol 26:957–969 10.1038/s41580-025-00899-0 41023495

[CR58] Fischer TD, Wang C, Padman BS, Lazarou M, Youle RJ (2020) STING induces LC3B lipidation onto single-membrane vesicles via the V-ATPase and ATG16L1-WD40 domain. J Cell Biol 219:e202009128 10.1083/jcb.202009128 PMC7716379 33201170

[CR59] Flickinger CJ (1969) Fenestrated cisternae in the Golgi apparatus of the epididymis. Anat Rec 163:39–53 10.1002/ar.1091630105 5763134

[CR60] Fougere L, Grison M, Laquel P, Montrazi M, Cordelieres F, Fernandez-Monreal M, Poujol C, Uemura T, Nakano A, Ito Y et al (2025) ER-to-Golgi trafficking through a dynamic intermediate cis-Golgi tubular network in Arabidopsis. Nat Cell Biol 27:424–437 10.1038/s41556-025-01624-x 40000850

[CR61] Gao Y, Zheng X, Chang B, Lin Y, Huang X, Wang W, Ding S, Zhan W, Wang S, Xiao B et al (2022) Intercellular transfer of activated STING triggered by RAB22A-mediated non-canonical autophagy promotes antitumor immunity. Cell Res 32:1086–1104 10.1038/s41422-022-00731-w PMC9715632 36280710

[CR62] Ge L, Baskaran S, Schekman R, Hurley JH (2014a) The protein-vesicle network of autophagy. Curr Opin Cell Biol 29:18–24 10.1016/j.ceb.2014.02.005 24681112

[CR63] Ge L, Melville D, Zhang M, Schekman R (2013) The ER-Golgi intermediate compartment is a key membrane source for the LC3 lipidation step of autophagosome biogenesis. eLife 2:e00947 10.7554/eLife.00947 PMC3736544 23930225

[CR64] Ge L, Schekman R (2014) The ER-Golgi intermediate compartment feeds the phagophore membrane. Autophagy 10:170–172 10.4161/auto.26787 PMC4389871 24220263

[CR65] Ge L, Wilz L, Schekman R (2015) Biogenesis of autophagosomal precursors for LC3 lipidation from the ER-Golgi intermediate compartment. Autophagy 11:2372–2374 10.1080/15548627.2015.1105422 PMC4835199 26565421

[CR66] Ge L, Zhang M, Kenny SJ, Liu D, Maeda M, Saito K, Mathur A, Xu K, Schekman R (2017) Remodeling of ER-exit sites initiates a membrane supply pathway for autophagosome biogenesis. EMBO Rep 18:1586–1603 10.15252/embr.201744559 PMC5579361 28754694

[CR67] Ge L, Zhang M, Schekman R (2014b) Phosphatidylinositol 3-kinase and COPII generate LC3 lipidation vesicles from the ER-Golgi intermediate compartment. eLife 3:e04135 10.7554/eLife.04135 PMC4270069 25432021

[CR68] Ghosh S, Dellibovi-Ragheb TA, Kerviel A, Pak E, Qiu Q, Fisher M, Takvorian PM, Bleck C, Hsu VW, Fehr AR et al (2020) β-coronaviruses use lysosomes for egress instead of the biosynthetic secretory pathway. Cell 183:1520–1535.e1514 10.1016/j.cell.2020.10.039 PMC7590812 33157038

[CR69] Gilbert A, Jadot M, Leontieva E, Wattiaux-De Coninck S, Wattiaux R (1998) Delta F508 CFTR localizes in the endoplasmic reticulum-Golgi intermediate compartment in cystic fibrosis cells. Exp Cell Res 242:144–152 10.1006/excr.1998.4101 9665812

[CR70] Gomez-Navarro N, Miller E (2016) Protein sorting at the ER-Golgi interface. J Cell Biol 215:769–778 10.1083/jcb.201610031 PMC5166505 27903609

[CR71] Gommel DU, Memon AR, Heiss A, Lottspeich F, Pfannstiel J, Lechner J, Reinhard C, Helms JB, Nickel W, Wieland FT (2001) Recruitment to Golgi membranes of ADP-ribosylation factor 1 is mediated by the cytoplasmic domain of p23. EMBO J 20:6751–6760 10.1093/emboj/20.23.6751 PMC125325 11726511

[CR72] Graef M, Friedman JR, Graham C, Babu M, Nunnari J (2013) ER exit sites are physical and functional core autophagosome biogenesis components. Mol Biol Cell 24:2918–2931 10.1091/mbc.E13-07-0381 PMC3771953 23904270

[CR73] Gui X, Yang H, Li T, Tan X, Shi P, Li M, Du F, Chen ZJ (2019) Autophagy induction via STING trafficking is a primordial function of the cGAS pathway. Nature 567:262–266 10.1038/s41586-019-1006-9 PMC9417302 30842662

[CR74] Guo R, Yan X, Li Y, Cui J, Misra S, Firth AE, Snijder EJ, Fang Y (2021) A swine arterivirus deubiquitinase stabilizes two major envelope proteins and promotes production of viral progeny. PLoS Pathog 17:e1009403 10.1371/journal.ppat.1009403 PMC7971519 33735221

[CR75] Hammond C, Helenius A (1994) Quality control in the secretory pathway: retention of a misfolded viral membrane glycoprotein involves cycling between the ER, intermediate compartment, and Golgi apparatus. J Cell Biol 126:41–52 10.1083/jcb.126.1.41 PMC2120101 8027184

[CR76] Han Y, Li S, Ge L (2023a) Biogenesis of autophagosomes from the ERGIC membrane system. J Genet Genomics 50:3–6 10.1016/j.jgg.2022.07.001 35835319

[CR77] Han Y, Zheng J, Ge L (2023b) Activated STING1 rides the Rafeesome. Autophagy 19:3230–3233 10.1080/15548627.2023.2240154 PMC10621249 37543953

[CR78] Hanna MGT, Block S, Frankel EB, Hou F, Johnson A, Yuan L, Knight G, Moresco JJ, Yates JR 3rd, Ashton R et al (2017) TFG facilitates outer coat disassembly on COPII transport carriers to promote tethering and fusion with ER-Golgi intermediate compartments. Proc Natl Acad Sci USA 114:E7707–E7716 10.1073/pnas.1709120114 PMC5604033 28851831

[CR79] Hanus C, Geptin H, Tushev G, Garg S, Alvarez-Castelao B, Sambandan S, Kochen L, Hafner AS, Langer JD, Schuman EM (2016) Unconventional secretory processing diversifies neuronal ion channel properties. eLife 5:e20609 10.7554/eLife.20609 PMC5077297 27677849

[CR80] Hanus C, Kochen L, Tom Dieck S, Racine V, Sibarita JB, Schuman EM, Ehlers MD (2014) Synaptic control of secretory trafficking in dendrites. Cell Rep 7:1771–1778 10.1016/j.celrep.2014.05.028 PMC5321479 24931613

[CR81] Hata Y, Slaughter CA, Sudhof TC (1993) Synaptic vesicle fusion complex contains unc-18 homologue bound to syntaxin. Nature 366:347–351 10.1038/366347a0 8247129

[CR82] Hauri HP, Kappeler F, Andersson H, Appenzeller C (2000) ERGIC-53 and traffic in the secretory pathway. J Cell Sci 113(Pt 4):587–596 10.1242/jcs.113.4.587 10652252

[CR83] Hermosilla R, Oueslati M, Donalies U, Schonenberger E, Krause E, Oksche A, Rosenthal W, Schulein R (2004) Disease-causing V(2) vasopressin receptors are retained in different compartments of the early secretory pathway. Traffic 5:993–1005 10.1111/j.1600-0854.2004.00239.x 15522100

[CR84] Hirschenberger M, Lepelley A, Rupp U, Klute S, Hunszinger V, Koepke L, Merold V, Didry-Barca B, Wondany F, Bergner T et al (2023) ARF1 prevents aberrant type I interferon induction by regulating STING activation and recycling. Nat Commun 14:6770 10.1038/s41467-023-42150-4 PMC10620153 37914730

[CR85] Hogue BG, Machamer CE (2007) Coronavirus structural proteins and virus assembly. Nidoviruses 179–200

[CR86] Hooper KM, Jacquin E, Li T, Goodwin JM, Brumell JH, Durgan J, Florey O (2022) V-ATPase is a universal regulator of LC3-associated phagocytosis and non-canonical autophagy. J Cell Biol 221:e202105112 10.1083/jcb.202105112 PMC9082624 35511089

[CR87] Huang T, Sun C, Du F, Chen ZJ (2025a) STING-induced noncanonical autophagy regulates endolysosomal homeostasis. Proc Natl Acad Sci USA 122:e2415422122 10.1073/pnas.2415422122 PMC11874320 39982740

[CR88] Huang Y, Xu F, Wang L, Mei S, Zhao F, Wang L, Xie Y, Wei L, Hu Y, Gao Z et al (2025b) STING inhibits LINE-1 retrotransposition through sorting ORF1p to lysosomes for degradation. EMBO Rep 26:4607–4630 10.1038/s44319-025-00551-0 PMC12457603 40825873

[CR89] Hurley JH, Young LN (2017) Mechanisms of autophagy initiation. Annu Rev Biochem 86:225–244 10.1146/annurev-biochem-061516-044820 PMC5604869 28301741

[CR90] Huttunen M, Samolej J, Evans RJ, Yakimovich A, White IJ, Kriston-Vizi J, Martin-Serrano J, Sundquist WI, Frickel EM, Mercer J (2021) Vaccinia virus hijacks ESCRT-mediated multivesicular body formation for virus egress. Life Sci Alliance 4:e202000910 10.26508/lsa.202000910 PMC8321658 34145027

[CR91] Imamura I, Kawaguchi S, Suzuki S, Kamiya Y, Ohnishi Y, Ueda J, Nashiki K, Takegawa K, Tabuchi M, Tanaka N (2025) Identification of an ERGIC-like compartment in fission yeast: Emp43 functions as a lectin-like cargo receptor for glycosylated proteins. Mol Microbiol 125:27–46 10.1111/mmi.70033 41203586

[CR92] Ishihara N, Hamasaki M, Yokota S, Suzuki K, Kamada Y, Kihara A, Yoshimori T, Noda T, Ohsumi Y (2001) Autophagosome requires specific early Sec proteins for its formation and NSF/SNARE for vacuolar fusion. Mol Biol Cell 12:3690–3702 10.1091/mbc.12.11.3690 PMC60286 11694599

[CR93] Ishikawa Y, Ito S, Nagata K, Sakai LY, Bachinger HP (2016) Intracellular mechanisms of molecular recognition and sorting for transport of large extracellular matrix molecules. Proc Natl Acad Sci USA 113:E6036–E6044 10.1073/pnas.1609571113 PMC5068301 27679847

[CR94] Itin C, Roche AC, Monsigny M, Hauri HP (1996) ERGIC-53 is a functional mannose-selective and calcium-dependent human homologue of leguminous lectins. Mol Biol Cell 7:483–493 10.1091/mbc.7.3.483 PMC275899 8868475

[CR95] Jamieson JD, Palade GE (1967) Intracellular transport of secretory proteins in the pancreatic exocrine cell. I. Role of the peripheral elements of the Golgi complex. J Cell Biol 34:577–596 10.1083/jcb.34.2.577 PMC2107305 6035647

[CR96] Jeong YT, Simoneschi D, Keegan S, Melville D, Adler NS, Saraf A, Florens L, Washburn MP, Cavasotto CN, Fenyo D et al (2018) The ULK1-FBXW5-SEC23B nexus controls autophagy. eLife 7:e42253 10.7554/eLife.42253 PMC6351106 30596474

[CR97] Jiao M, Wang C, Tang X, Dai C, Zhang N, Fan A, Qian Z, Liu S, Zhang F, Li B et al (2024) Active secretion of IL-33 from astrocytes is dependent on TMED10 and promotes central nervous system homeostasis. Brain Behav Immun 119:539–553 10.1016/j.bbi.2024.04.028 38663774

[CR98] Jin H, Komita M, Aoe T (2017) The role of BiP retrieval by the KDEL receptor in the early secretory pathway and its effect on protein quality control and neurodegeneration. Front Mol Neurosci 10:222 10.3389/fnmol.2017.00222 PMC5511815 28769758

[CR99] Jin L, Pahuja KB, Wickliffe KE, Gorur A, Baumgartel C, Schekman R, Rape M (2012) Ubiquitin-dependent regulation of COPII coat size and function. Nature 482:495–500 10.1038/nature10822 PMC3292188 22358839

[CR100] Johnson A, Bhattacharya N, Hanna M, Pennington JG, Schuh AL, Wang L, Otegui MS, Stagg SM, Audhya A (2015) TFG clusters COPII-coated transport carriers and promotes early secretory pathway organization. EMBO J 34:811–827 10.15252/embj.201489032 PMC4369316 25586378

[CR101] Kabeya Y, Mizushima N, Ueno T, Yamamoto A, Kirisako T, Noda T, Kominami E, Ohsumi Y, Yoshimori T (2000) LC3, a mammalian homologue of yeast Apg8p, is localized in autophagosome membranes after processing. EMBO J 19:5720–5728 10.1093/emboj/19.21.5720 PMC305793 11060023

[CR102] Kakuta S, Yamamoto H, Negishi L, Kondo-Kakuta C, Hayashi N, Ohsumi Y (2012) Atg9 vesicles recruit vesicle-tethering proteins Trs85 and Ypt1 to the autophagosome formation site. J Biol Chem 287:44261–44269 10.1074/jbc.M112.411454 PMC3531741 23129774

[CR103] Kappeler F, Klopfenstein DR, Foguet M, Paccaud JP, Hauri HP (1997) The recycling of ERGIC-53 in the early secretory pathway. ERGIC-53 carries a cytosolic endoplasmic reticulum-exit determinant interacting with COPII. J Biol Chem 272:31801–31808 10.1074/jbc.272.50.31801 9395526

[CR104] Karagoz GE, Acosta-Alvear D, Walter P (2019) The unfolded protein response: detecting and responding to fluctuations in the protein-folding capacity of the endoplasmic reticulum. Cold Spring Harb Perspect Biol 11:a033886 10.1101/cshperspect.a033886 PMC6719602 30670466

[CR105] Karanasios E, Walker SA, Okkenhaug H, Manifava M, Hummel E, Zimmermann H, Ahmed Q, Domart MC, Collinson L, Ktistakis NT (2016) Autophagy initiation by ULK complex assembly on ER tubulovesicular regions marked by ATG9 vesicles. Nat Commun 7:12420 10.1038/ncomms12420 PMC4987534 27510922

[CR106] Kayagaki N, Stowe IB, Lee BL, O’Rourke K, Anderson K, Warming S, Cuellar T, Haley B, Roose-Girma M, Phung QT et al (2015) Caspase-11 cleaves gasdermin D for non-canonical inflammasome signalling. Nature 526:666–671 10.1038/nature15541 26375259

[CR107] Kim J, Gee HY, Lee MG (2018) Unconventional protein secretion—new insights into the pathogenesis and therapeutic targets of human diseases. J Cell Sci 131:jcs213686 10.1242/jcs.213686 29941450

[CR108] Klaus JP, Eisenhauer P, Russo J, Mason AB, Do D, King B, Taatjes D, Cornillez-Ty C, Boyson JE, Thali M et al (2013) The intracellular cargo receptor ERGIC-53 is required for the production of infectious arenavirus, coronavirus, and filovirus particles. Cell Host Microbe 14:522–534 10.1016/j.chom.2013.10.010 PMC3999090 24237698

[CR109] Klumperman J, Locker JK, Meijer A, Horzinek MC, Geuze HJ, Rottier PJ (1994) Coronavirus M proteins accumulate in the Golgi complex beyond the site of virion budding. J Virol 68:6523–6534 10.1128/jvi.68.10.6523-6534.1994 PMC237073 8083990

[CR110] Klumperman J, Schweizer A, Clausen H, Tang BL, Hong W, Oorschot V, Hauri HP (1998) The recycling pathway of protein ERGIC-53 and dynamics of the ER-Golgi intermediate compartment. J Cell Sci 111(Pt 22):3411–3425 10.1242/jcs.111.22.3411 9788882

[CR111] Ladinsky MS, Mastronarde DN, McIntosh JR, Howell KE, Staehelin LA (1999) Golgi structure in three dimensions: functional insights from the normal rat kidney cell. J Cell Biol 144:1135–1149 10.1083/jcb.144.6.1135 PMC2150572 10087259

[CR112] Lahtinen U, Hellman U, Wernstedt C, Saraste J, Pettersson RF (1996) Molecular cloning and expression of a 58-kDa cis-Golgi and intermediate compartment protein. J Biol Chem 271:4031–4037 10.1074/jbc.271.8.4031 8626736

[CR113] Lamb CA, Nuhlen S, Judith D, Frith D, Snijders AP, Behrends C, Tooze SA (2016) TBC1D14 regulates autophagy via the TRAPP complex and ATG9 traffic. EMBO J 35:281–301 10.15252/embj.201592695 PMC4741301 26711178

[CR114] Lamb CA, Yoshimori T, Tooze SA (2013) The autophagosome: origins unknown, biogenesis complex. Nat Rev Mol Cell Biol 14:759–774 10.1038/nrm3696 24201109

[CR115] Lee MC, Miller EA, Goldberg J, Orci L, Schekman R (2004) Bi-directional protein transport between the ER and Golgi. Annu Rev Cell Dev Biol 20:87–123 10.1146/annurev.cellbio.20.010403.105307 15473836

[CR116] Lee MC, Orci L, Hamamoto S, Futai E, Ravazzola M, Schekman R (2005) Sar1p N-terminal helix initiates membrane curvature and completes the fission of a COPII vesicle. Cell 122:605–617 10.1016/j.cell.2005.07.025 16122427

[CR117] Levine B, Kroemer G (2019) Biological functions of autophagy genes: a disease perspective. Cell 176:11–42 10.1016/j.cell.2018.09.048 PMC6347410 30633901

[CR118] Levy E, Poinsot P, Spahis S (2019) Chylomicron retention disease: genetics, biochemistry, and clinical spectrum. Curr Opin Lipidol 30:134–139 10.1097/MOL.0000000000000578 30640893

[CR119] Lewis MJ, Pelham HR (1992) Ligand-induced redistribution of a human KDEL receptor from the Golgi complex to the endoplasmic reticulum. Cell 68:353–364 10.1016/0092-8674(92)90476-s 1310258

[CR120] Li J, Tan JX, Chen ZJ, Zhang X, Bai XC (2026) Regulation of STING activation by phosphoinositide and cholesterol. Nature 652:499–507 10.1038/s41586-025-10076-0 PMC13224288 41639452

[CR121] Li S, Yan R, Xu J, Zhao S, Ma X, Sun Q, Zhang M, Li Y, Liu JG, Chen L et al (2022) A new type of ERGIC-ERES membrane contact mediated by TMED9 and SEC12 is required for autophagosome biogenesis. Cell Res 32:119–138 10.1038/s41422-021-00563-0 PMC8461442 34561617

[CR122] Li S, Zhang M, Ge L (2021) A new type of membrane contact in the ER-Golgi system regulates autophagosome biogenesis. Autophagy 17:4499–4501 10.1080/15548627.2021.1972406 PMC8726631 34643464

[CR123] Li T, Yang F, Heng Y, Zhou S, Wang G, Wang J, Wang J, Chen X, Yao ZP, Wu Z et al (2023) TMED10 mediates the trafficking of insulin-like growth factor 2 along the secretory pathway for myoblast differentiation. Proc Natl Acad Sci USA 120:e2215285120 10.1073/pnas.2215285120 PMC10655563 37931110

[CR124] Lipatova Z, Belogortseva N, Zhang XQ, Kim J, Taussig D, Segev N (2012) Regulation of selective autophagy onset by a Ypt/Rab GTPase module. Proc Natl Acad Sci USA 109:6981–6986 10.1073/pnas.1121299109 PMC3344974 22509044

[CR125] Lippincott-Schwartz J, Donaldson JG, Schweizer A, Berger EG, Hauri HP, Yuan LC, Klausner RD (1990) Microtubule-dependent retrograde transport of proteins into the ER in the presence of brefeldin A suggests an ER recycling pathway. Cell 60:821–836 10.1016/0092-8674(90)90096-w 2178778

[CR126] Liu B, Carlson RJ, Pires IS, Gentili M, Feng E, Hellier Q, Schwartz MA, Blainey PC, Irvine DJ, Hacohen N (2023) Human STING is a proton channel. Science 381:508–514 10.1126/science.adf8974 PMC11260435 37535724

[CR127] Liu L, Zhang L, Hao X, Wang Y, Zhang X, Ge L, Wang P, Tian B, Zhang M (2024) Coronavirus envelope protein activates TMED10-mediated unconventional secretion of inflammatory factors. Nat Commun 15:8708 10.1038/s41467-024-52818-0 PMC11461611 39379362

[CR128] Lolicato F, Saleppico R, Griffo A, Meyer A, Scollo F, Pokrandt B, Muller HM, Ewers H, Hahl H, Fleury JB et al (2022) Cholesterol promotes clustering of PI(4,5)P2 driving unconventional secretion of FGF2. J Cell Biol 221:e202106123 10.1083/jcb.202106123 PMC9526255 36173379

[CR129] Lord C, Ferro-Novick S, Miller EA (2013) The highly conserved COPII coat complex sorts cargo from the endoplasmic reticulum and targets it to the Golgi. Cold Spring Harb Perspect Biol 5:a013367 10.1101/cshperspect.a013367 PMC3552504 23378591

[CR130] Lorente-Rodriguez A, Barlowe C (2011) Entry and exit mechanisms at the cis-face of the Golgi complex. Cold Spring Harb Perspect Biol 3:a005207 10.1101/cshperspect.a005207 PMC3119910 21482742

[CR131] Lorincz P, Toth S, Benko P, Lakatos Z, Boda A, Glatz G, Zobel M, Bisi S, Hegedus K, Takats S et al (2017) Rab2 promotes autophagic and endocytic lysosomal degradation. J Cell Biol 216:1937–1947 10.1083/jcb.201611027 PMC5496615 28483915

[CR132] Lotti LV, Torrisi MR, Pascale MC, Bonatti S (1992) Immunocytochemical analysis of the transfer of vesicular stomatitis virus G glycoprotein from the intermediate compartment to the Golgi complex. J Cell Biol 118:43–50 10.1083/jcb.118.1.43 PMC2289513 1320035

[CR133] Lucocq JM, Brada D, Roth J (1986) Immunolocalization of the oligosaccharide trimming enzyme glucosidase II. J Cell Biol 102:2137–2146 10.1083/jcb.102.6.2137 PMC2114248 3519622

[CR134] Lynch-Day MA, Bhandari D, Menon S, Huang J, Cai H, Bartholomew CR, Brumell JH, Ferro-Novick S, Klionsky DJ (2010) Trs85 directs a Ypt1 GEF, TRAPPIII, to the phagophore to promote autophagy. Proc Natl Acad Sci USA 107:7811–7816 10.1073/pnas.1000063107 PMC2867920 20375281

[CR135] Lyu H, Xing C, Huai W, Song K, Jeltema D, Zhang H, Zhang X, Yan N (2026) STING signaling modulation by COPII cargo recognition. Cell 189:2971–2987 10.1016/j.cell.2026.02.029 PMC13052199 41887218

[CR136] Ma M, Dang Y, Chang B, Wang F, Xu J, Chen L, Su H, Li J, Ge B, Chen C et al (2023) TAK1 is an essential kinase for STING trafficking. Mol Cell 83:3885–3903 e3885 10.1016/j.molcel.2023.09.009 37832545

[CR137] Ma W, Goldberg J (2016) TANGO1/cTAGE5 receptor as a polyvalent template for assembly of large COPII coats. Proc Natl Acad Sci USA 113:10061–10066 10.1073/pnas.1605916113 PMC5018777 27551091

[CR138] Maldutyte J, Li XH, Gomez-Navarro N, Robertson EG, Miller EA (2025) ER export via SURF4 uses diverse mechanisms of both client and coat engagement. J Cell Biol 224:e202406103 10.1083/jcb.202406103 PMC11557686 39531033

[CR139] Malhotra V, Erlmann P (2015) The pathway of collagen secretion. Annu Rev Cell Dev Biol 31:109–124 10.1146/annurev-cellbio-100913-013002 26422332

[CR140] Manton I (1960) On a reticular derivative from Golgi bodies in the meristem of Anthroceros. J Biophys Biochem Cytol 8:221–231 10.1083/jcb.8.1.221 PMC2224922 13766334

[CR141] Marie M, Dale HA, Sannerud R, Saraste J (2009) The function of the intermediate compartment in pre-Golgi trafficking involves its stable connection with the centrosome. Mol Biol Cell 20:4458–4470 10.1091/mbc.E08-12-1229 PMC2762134 19710425

[CR142] Maul GG, Brinkley BR (1970) The golgi apparatus during mitosis in human melanoma cells in vitro. Cancer Res 30:2326–2335 5475479

[CR143] McCaughey J, Stephens DJ (2019) ER-to-Golgi transport: a sizeable problem. Trends Cell Biol 29:940–953 10.1016/j.tcb.2019.08.007 31630879

[CR144] Medeiros-Silva J, Dregni AJ, Somberg NH, Duan P, Hong M (2023) Atomic structure of the open SARS-CoV-2 E viroporin. Sci Adv 9:eadi9007 10.1126/sciadv.adi9007 PMC10575589 37831764

[CR145] Melia TJ (2023) Growing thin—how bulk lipid transport drives expansion of the autophagosome membrane but not of its lumen. Curr Opin Cell Biol 83:102190 10.1016/j.ceb.2023.102190 PMC10528516 37385155

[CR146] Miller EA, Beilharz TH, Malkus PN, Lee MC, Hamamoto S, Orci L, Schekman R (2003) Multiple cargo binding sites on the COPII subunit Sec24p ensure capture of diverse membrane proteins into transport vesicles. Cell 114:497–509 10.1016/s0092-8674(03)00609-3 12941277

[CR147] Miller EA, Schekman R (2013) COPII - a flexible vesicle formation system. Curr Opin Cell Biol 25:420–427 10.1016/j.ceb.2013.04.005 PMC3736695 23702145

[CR148] Mollenhauer HH (1959) Permanganate fixation of plant cells. J Biophys Biochem Cytol 6:431–436 10.1083/jcb.6.3.431 PMC2224711 14423414

[CR149] Mollenhauer HH, Morre DJ (1966) Tubular connections between dictyosomes and forming secretory vesicles in plant Golgi apparatus. J Cell Biol 29:373–376 10.1083/jcb.29.2.373 PMC2106909 5961348

[CR150] Mollenhauer HH, Morre DJ (1998) The tubular network of the Golgi apparatus. Histochem Cell Biol 109:533–543 10.1007/s004180050253 9681633

[CR151] Monetta P, Slavin I, Romero N, Alvarez C (2007) Rab1b interacts with GBF1 and modulates both ARF1 dynamics and COPI association. Mol Biol Cell 18:2400–2410 10.1091/mbc.E06-11-1005 PMC1924811 17429068

[CR152] Nagae M, Hirata T, Morita-Matsumoto K, Theiler R, Fujita M, Kinoshita T, Yamaguchi Y (2016) 3D structure and interaction of p24beta and p24delta Golgi dynamics domains: implication for p24 complex formation and cargo transport. J Mol Biol 428:4087–4099 10.1016/j.jmb.2016.08.023 27569046

[CR153] Nakatogawa H (2020) Mechanisms governing autophagosome biogenesis. Nat Rev Mol Cell Biol 21:439–458 10.1038/s41580-020-0241-0 32372019

[CR154] Nal B, Chan C, Kien F, Siu L, Tse J, Chu K, Kam J, Staropoli I, Crescenzo-Chaigne B, Escriou N et al (2005) Differential maturation and subcellular localization of severe acute respiratory syndrome coronavirus surface proteins S, M and E. J Gen Virol 86:1423–1434 10.1099/vir.0.80671-0 15831954

[CR155] Nan Y, Cui D, Guo J, Ma X, Wang J, Guo L, Li T, Yang M, Huang G, Xu A et al (2025) STING COPII ER export trafficking and signaling primed by phosphorylation switches. Adv Sci 12:e03660 10.1002/advs.202503660 PMC12463132 40598830

[CR156] Nie C, Wang H, Wang R, Ginsburg D, Chen XW (2018) Dimeric sorting code for concentrative cargo selection by the COPII coat. Proc Natl Acad Sci USA 115:E3155–E3162 10.1073/pnas.1704639115 PMC5889621 29555761

[CR157] Nishio M, Kamiya Y, Mizushima T, Wakatsuki S, Sasakawa H, Yamamoto K, Uchiyama S, Noda M, McKay AR, Fukui K et al (2010) Structural basis for the cooperative interplay between the two causative gene products of combined factor V and factor VIII deficiency. Proc Natl Acad Sci USA 107:4034–4039 10.1073/pnas.0908526107 PMC2840101 20142513

[CR158] Novick P, Field C, Schekman R (1980) Identification of 23 complementation groups required for post-translational events in the yeast secretory pathway. Cell 21:205–215 10.1016/0092-8674(80)90128-2 6996832

[CR159] Novick P, Schekman R (1979) Secretion and cell-surface growth are blocked in a temperature-sensitive mutant of Saccharomyces cerevisiae. Proc Natl Acad Sci USA 76:1858–1862 10.1073/pnas.76.4.1858 PMC383491 377286

[CR160] Nufer O, Kappeler F, Guldbrandsen S, Hauri HP (2003) ER export of ERGIC-53 is controlled by cooperation of targeting determinants in all three of its domains. J Cell Sci 116:4429–4440 10.1242/jcs.00759 13130098

[CR161] Nyfeler B, Kamiya Y, Boehlen F, Yamamoto K, Kato K, de Moerloose P, Hauri HP, Neerman-Arbez M (2008) Deletion of 3 residues from the C-terminus of MCFD2 affects binding to ERGIC-53 and causes combined factor V and factor VIII deficiency. Blood 111:1299–1301 10.1182/blood-2007-09-112854 17971482

[CR162] Palade G (1975) Intracellular aspects of the process of protein synthesis. Science 189:867 10.1126/science.189.4206.867-b 17812524

[CR163] Palade GE (1955) A small particulate component of the cytoplasm. J Biophys Biochem Cytol 1:59–68 10.1083/jcb.1.1.59 PMC2223592 14381428

[CR164] Palade GE (1956) The endoplasmic reticulum. J Biophys Biochem Cytol 2:85–98 10.1083/jcb.2.4.85 PMC2229739 13357527

[CR165] Palade GE, Claude A (1949) The nature of the Golgi apparatus; parallelism between intercellular myelin figures and Golgi apparatus in somatic cells. J Morphol 85:35–69 10.1002/jmor.1050850103 18135694

[CR166] Palade GE, Porter KR (1954) Studies on the endoplasmic reticulum: I. Its identification in cells in situ. J Exp Med 100:641–656 10.1084/jem.100.6.641 PMC2136401 13211920

[CR167] Pallotta MT, Nickel W (2020) FGF2 and IL-1beta—explorers of unconventional secretory pathways at a glance. J Cell Sci 133:jcs250449 10.1242/jcs.250449 33154173

[CR168] Pannwitt S, Stangl M, Schneider D (2019) Lipid binding controls dimerization of the coat protein p24 transmembrane helix. Biophys J 117:1554–1562 10.1016/j.bpj.2019.09.021 PMC6838755 31627840

[CR169] Parchure A, Tejada H, Xi Z, Kim Y, Su M, Yan Y, Julca-Zevallos O, Alcazar-Roman AR, Villemeur M, Liu X et al (2025) TUG protein acts through a disordered region to organize the early secretory pathway. Nat Commun 16:5518 10.1038/s41467-025-60691-8 PMC12218103 40593538

[CR170] Pastor-Cantizano N, Montesinos JC, Bernat-Silvestre C, Marcote MJ, Aniento F (2016) p24 family proteins: key players in the regulation of trafficking along the secretory pathway. Protoplasma 253:967–985 10.1007/s00709-015-0858-6 26224213

[CR171] Peter F, Plutner H, Zhu H, Kreis TE, Balch WE (1993) Beta-COP is essential for transport of protein from the endoplasmic reticulum to the Golgi in vitro. J Cell Biol 122:1155–1167 10.1083/jcb.122.6.1155 PMC2119854 8376457

[CR172] Popoff V, Adolf F, Brugger B, Wieland F (2011a) COPI budding within the Golgi stack. Cold Spring Harb Perspect Biol 3:a005231 10.1101/cshperspect.a005231 PMC3220356 21844168

[CR173] Popoff V, Langer JD, Reckmann I, Hellwig A, Kahn RA, Brugger B, Wieland FT (2011b) Several ADP-ribosylation factor (Arf) isoforms support COPI vesicle formation. J Biol Chem 286:35634–35642 10.1074/jbc.M111.261800 PMC3195608 21844198

[CR174] Porter KR, Claude A, Fullam EF (1945) A study of tissue culture cells by electron microscopy: methods and preliminary observations. J Exp Med 81:233–246 10.1084/jem.81.3.233 PMC2135493 19871454

[CR175] Presley JF, Cole NB, Schroer TA, Hirschberg K, Zaal KJ, Lippincott-Schwartz J (1997) ER-to-Golgi transport visualized in living cells. Nature 389:81–85 10.1038/38001 9288971

[CR176] Puri C, Rubinsztein DC (2022) TMED9-SEC12, an important “contact” for autophagy. Cell Res 32:111–112 10.1038/s41422-021-00579-6 PMC8807701 34625661

[CR177] Qiu H, Wu X, Ma X, Li S, Cai Q, Ganzella M, Ge L, Zhang H, Zhang M (2024) Short-distance vesicle transport via phase separation. Cell 187:2175–2193 e2121 10.1016/j.cell.2024.03.003 38552623

[CR178] Rabouille C (2017) Pathways of unconventional protein secretion. Trends Cell Biol 27:230–240 10.1016/j.tcb.2016.11.007 27989656

[CR179] Ramanathan HN, Chung DH, Plane SJ, Sztul E, Chu YK, Guttieri MC, McDowell M, Ali G, Jonsson CB (2007) Dynein-dependent transport of the hantaan virus nucleocapsid protein to the endoplasmic reticulum-Golgi intermediate compartment. J Virol 81:8634–8647 10.1128/JVI.00418-07 PMC1951367 17537852

[CR180] Raote I, Malhotra V (2021) Tunnels for protein export from the endoplasmic reticulum. Annu Rev Biochem 90:605–630 10.1146/annurev-biochem-080120-022017 33503381

[CR181] Raote I, Ortega-Bellido M, Santos AJ, Foresti O, Zhang C, Garcia-Parajo MF, Campelo F, Malhotra V (2018) TANGO1 builds a machine for collagen export by recruiting and spatially organizing COPII, tethers and membranes. eLife 7:e32723 10.7554/eLife.32723 PMC5851698 29513218

[CR182] Raote I, Rosendahl AH, Hakkinen HM, Vibe C, Kucukaylak I, Sawant M, Keufgens L, Frommelt P, Halwas K, Broadbent K et al (2024) TANGO1 inhibitors reduce collagen secretion and limit tissue scarring. Nat Commun 15:3302 10.1038/s41467-024-47004-1 PMC11043333 38658535

[CR183] Raposo G, van Santen HM, Leijendekker R, Geuze HJ, Ploegh HL (1995) Misfolded major histocompatibility complex class I molecules accumulate in an expanded ER-Golgi intermediate compartment. J Cell Biol 131:1403–1419 10.1083/jcb.131.6.1403 PMC2120650 8522600

[CR184] Raykhel I, Alanen H, Salo K, Jurvansuu J, Nguyen VD, Latva-Ranta M, Ruddock L (2007) A molecular specificity code for the three mammalian KDEL receptors. J Cell Biol 179:1193–1204 10.1083/jcb.200705180 PMC2140024 18086916

[CR185] Risco C, Rodríguez JR, López-Iglesias C, Carrascosa JL, Esteban M, Rodríguez D (2002) Endoplasmic reticulum-Golgi intermediate compartment membranes and vimentin filaments participate in vaccinia virus assembly. J Virol 76:1839–1855 10.1128/JVI.76.4.1839-1855.2002 PMC135913 11799179

[CR186] Ronzier E, Satpute-Krishnan P (2025) TMED9 coordinates the clearance of misfolded GPI-anchored proteins out of the ER and into the Golgi. PLoS Biol 23:e3003084 10.1371/journal.pbio.3003084 PMC12052135 40203033

[CR187] Roth J, Zuber C (2017) Quality control of glycoprotein folding and ERAD: the role of N-glycan handling, EDEM1 and OS-9. Histochem Cell Biol 147:269–284 10.1007/s00418-016-1513-9 27803995

[CR188] Rothman JE, Wieland FT (1996) Protein sorting by transport vesicles. Science 272:227–234 10.1126/science.272.5259.227 8602507

[CR189] Saenz JB, Sun WJ, Chang JW, Li J, Bursulaya B, Gray NS, Haslam DB (2009) Golgicide A reveals essential roles for GBF1 in Golgi assembly and function. Nat Chem Biol 5:157–165 10.1038/nchembio.144 PMC3500152 19182783

[CR190] Saito K, Chen M, Bard F, Chen S, Zhou H, Woodley D, Polischuk R, Schekman R, Malhotra V (2009) TANGO1 facilitates cargo loading at endoplasmic reticulum exit sites. Cell 136:891–902 10.1016/j.cell.2008.12.025 19269366

[CR191] Saito K, Yamashiro K, Ichikawa Y, Erlmann P, Kontani K, Malhotra V, Katada T (2011) cTAGE5 mediates collagen secretion through interaction with TANGO1 at endoplasmic reticulum exit sites. Mol Biol Cell 22:2301–2308 10.1091/mbc.E11-02-0143 PMC3128532 21525241

[CR192] Salanueva IJ, Carrascosa JL, Risco C (1999) Structural maturation of the transmissible gastroenteritis coronavirus. J Virol 73:7952–7964 10.1128/jvi.73.10.7952-7964.1999 PMC112809 10482542

[CR193] Sannerud R, Marie M, Nizak C, Dale HA, Pernet-Gallay K, Perez F, Goud B, Saraste J (2006) Rab1 defines a novel pathway connecting the pre-Golgi intermediate compartment with the cell periphery. Mol Biol Cell 17:1514–1526 10.1091/mbc.E05-08-0792 PMC1415313 16421253

[CR194] Santos AJ, Raote I, Scarpa M, Brouwers N, Malhotra V (2015) TANGO1 recruits ERGIC membranes to the endoplasmic reticulum for procollagen export. eLife 4:e10982 10.7554/eLife.10982 PMC4709264 26568311

[CR195] Saraste J (2016) Spatial and functional aspects of ER-Golgi Rabs and tethers. Front Cell Dev Biol 4:28 10.3389/fcell.2016.00028 PMC4834429 27148530

[CR196] Saraste J, Enyioko M, Dale H, Prydz K, Machamer C (2022) Evidence for the role of Rab11-positive recycling endosomes as intermediates in coronavirus egress from epithelial cells. Histochem Cell Biol 158:241–251 10.1007/s00418-022-02115-y PMC9124743 35604431

[CR197] Saraste J, Kuismanen E (1984) Pre- and post-Golgi vacuoles operate in the transport of Semliki Forest virus membrane glycoproteins to the cell surface. Cell 38:535–549 10.1016/0092-8674(84)90508-7 6432345

[CR198] Saraste J, Marie M (2018) Intermediate compartment (IC): from pre-Golgi vacuoles to a semi-autonomous membrane system. Histochem Cell Biol 150:407–430 10.1007/s00418-018-1717-2 PMC6182704 30173361

[CR199] Saraste J, Palade GE, Farquhar MG (1986) Temperature-sensitive steps in the transport of secretory proteins through the Golgi complex in exocrine pancreatic cells. Proc Natl Acad Sci USA 83:6425–6429 10.1073/pnas.83.17.6425 PMC386516 3462704

[CR200] Saraste J, Palade GE, Farquhar MG (1987) Antibodies to rat pancreas Golgi subfractions: identification of a 58-kD cis-Golgi protein. J Cell Biol 105:2021–2029 10.1083/jcb.105.5.2021 PMC2114852 3316245

[CR201] Saraste J, Prydz K (2026) Unconventional protein transport across the Golgi ribbon. Subcell Biochem 110:67–93 10.1007/978-3-032-06936-8_4 41240308

[CR202] Saraste J, Svensson K (1991) Distribution of the intermediate elements operating in ER to Golgi transport. J Cell Sci 100(Pt 3):415–430 10.1242/jcs.100.3.415 1808196

[CR203] Saraste J, Marie M (2015) Intermediate compartment: a sorting station between the endoplasmic reticulum and the Golgi apparatus. Encyclopedia Cell Biol 2: 168–182

[CR204] Satoh T, Suzuki K, Yamaguchi T, Kato K (2014) Structural basis for disparate sugar-binding specificities in the homologous cargo receptors ERGIC-53 and VIP36. PLoS ONE 9:e87963 10.1371/journal.pone.0087963 PMC3912170 24498414

[CR205] Satpute-Krishnan P, Ajinkya M, Bhat S, Itakura E, Hegde RS, Lippincott-Schwartz J (2014) ER stress-induced clearance of misfolded GPI-anchored proteins via the secretory pathway. Cell 158:522–533 10.1016/j.cell.2014.06.026 PMC4121523 25083867

[CR206] Saxena S, Foresti O, Liu A, Androulaki S, Pena Rodriguez M, Raote I, Aridor M, Cui B, Malhotra V (2024) Endoplasmic reticulum exit sites are segregated for secretion based on cargo size. Dev Cell 59:2593–2608 e2596 10.1016/j.devcel.2024.06.009 PMC11813558 38991587

[CR207] Scales SJ, Pepperkok R, Kreis TE (1997) Visualization of ER-to-Golgi transport in living cells reveals a sequential mode of action for COPII and COPI. Cell 90:1137–1148 10.1016/s0092-8674(00)80379-7 9323141

[CR208] Schekman R, Orci L (1996) Coat proteins and vesicle budding. Science 271:1526–1533 10.1126/science.271.5255.1526 8599108

[CR209] Scherer KM, Mascheroni L, Carnell GW, Wunderlich LCS, Makarchuk S, Brockhoff M, Mela I, Fernandez-Villegas A, Barysevich M, Stewart H et al (2022) SARS-CoV-2 nucleocapsid protein adheres to replication organelles before viral assembly at the Golgi/ERGIC and lysosome-mediated egress. Sci Adv 8:eabl4895 10.1126/sciadv.abl4895 PMC10954198 34995113

[CR210] Schindler R, Itin C, Zerial M, Lottspeich F, Hauri HP (1993) ERGIC-53, a membrane protein of the ER-Golgi intermediate compartment, carries an ER retention motif. Eur J Cell Biol 61:1–9 8223692

[CR211] Schweizer A, Fransen JA, Bachi T, Ginsel L, Hauri HP (1988) Identification, by a monoclonal antibody, of a 53-kD protein associated with a tubulo-vesicular compartment at the cis-side of the Golgi apparatus. J Cell Biol 107:1643–1653 10.1083/jcb.107.5.1643 PMC2115344 3182932

[CR212] Schweizer A, Fransen JA, Matter K, Kreis TE, Ginsel L, Hauri HP (1990) Identification of an intermediate compartment involved in protein transport from endoplasmic reticulum to Golgi apparatus. Eur J Cell Biol 53:185–196 1964413

[CR213] Schweizer A, Matter K, Ketcham CM, Hauri HP (1991) The isolated ER-Golgi intermediate compartment exhibits properties that are different from ER and cis-Golgi. J Cell Biol 113:45–54 10.1083/jcb.113.1.45 PMC2288923 2007626

[CR214] Sesso A, de Faria FP, Iwamura ES, Correa H (1994) A three-dimensional reconstruction study of the rough ER-Golgi interface in serial thin sections of the pancreatic acinar cell of the rat. J Cell Sci 107(Pt 3):517–528 8006070

[CR215] Shi J, Zhao Y, Wang K, Shi X, Wang Y, Huang H, Zhuang Y, Cai T, Wang F, Shao F (2015) Cleavage of GSDMD by inflammatory caspases determines pyroptotic cell death. Nature 526:660–665 10.1038/nature15514 26375003

[CR216] Shima T, Kirisako H, Nakatogawa H (2019) COPII vesicles contribute to autophagosomal membranes. J Cell Biol 218:1503–1510 10.1083/jcb.201809032 PMC6504894 30787039

[CR217] Shomron O, Nevo-Yassaf I, Aviad T, Yaffe Y, Zahavi EE, Dukhovny A, Perlson E, Brodsky I, Yeheskel A, Pasmanik-Chor M et al (2021) COPII collar defines the boundary between ER and ER exit site and does not coat cargo containers. J Cell Biol 220:e201907224 10.1083/jcb.201907224 PMC8054201 33852719

[CR218] Sicari D, Chatziioannou A, Koutsandreas T, Sitia R, Chevet E (2020) Role of the early secretory pathway in SARS-CoV-2 infection. J Cell Biol 219:e202006005 10.1083/jcb.202006005 PMC7480111 32725137

[CR219] Sirkis DW, Aparicio RE, Schekman R (2017) Neurodegeneration-associated mutant TREM2 proteins abortively cycle between the ER and ER-Golgi intermediate compartment. Mol Biol Cell 28:2723–2733 10.1091/mbc.E17-06-0423 PMC5620379 28768830

[CR220] Sivan G, Weisberg AS, Americo JL, Moss B (2016) Retrograde transport from early endosomes to the trans-Golgi network enables membrane wrapping and egress of Vaccinia virus virions. J Virol 90:8891–8905 10.1128/JVI.01114-16 PMC5021392 27466413

[CR221] Solis GP, Bilousov O, Koval A, Luchtenborg AM, Lin C, Katanaev VL (2017) Golgi-resident galphao promotes protrusive membrane dynamics. Cell 170:1258 10.1016/j.cell.2017.08.045 28886387

[CR222] Song MS, Sim HJ, Eun SH, Jung MK, Hwang SJ, Ham MH, Kwak K, Lee HJ, Kim JY, Jang DG et al (2025) Tubular ER structures shaped by ER-phagy receptors engage in stress-induced Golgi bypass. Dev Cell 60:1568–1585 e1511 10.1016/j.devcel.2025.01.011 39919755

[CR223] Sparn C, Meyer A, Saleppico R, Nickel W (2022) Unconventional secretion mediated by direct protein self-translocation across the plasma membranes of mammalian cells. Trends Biochem Sci 47:699–709 10.1016/j.tibs.2022.04.001 35490075

[CR224] Stadel D, Millarte V, Tillmann KD, Huber J, Tamin-Yecheskel BC, Akutsu M, Demishtein A, Ben-Zeev B, Anikster Y, Perez F et al (2015) TECPR2 cooperates with LC3C to regulate COPII-dependent ER export. Mol Cell 60:89–104 10.1016/j.molcel.2015.09.010 26431026

[CR225] Strating JR, Martens GJ (2009) The p24 family and selective transport processes at the ER-Golgi interface. Biol Cell 101:495–509 10.1042/BC20080233 19566487

[CR226] Strating JR, van Bakel NH, Leunissen JA, Martens GJ (2009) A comprehensive overview of the vertebrate p24 family: identification of a novel tissue-specifically expressed member. Mol Biol Evol 26:1707–1714 10.1093/molbev/msp099 19429673

[CR227] Sturman LS, Holmes KV (1983) The molecular biology of coronaviruses. Adv Virus Res 28:35–112 10.1016/S0065-3527(08)60721-6 PMC7131312 6362367

[CR228] Sudhof TC, Rothman JE (2009) Membrane fusion: grappling with SNARE and SM proteins. Science 323:474–477 10.1126/science.1161748 PMC3736821 19164740

[CR229] Sun L, Wu J, Du F, Chen X, Chen ZJ (2013) Cyclic GMP-AMP synthase is a cytosolic DNA sensor that activates the type I interferon pathway. Science 339:786–791 10.1126/science.1232458 PMC3863629 23258413

[CR230] Sun Y, Tao X, Han Y, Lin X, Tian R, Wang H, Chang P, Sun Q, Ge L, Zhang M (2024) A dual role of ERGIC-localized Rabs in TMED10-mediated unconventional protein secretion. Nat Cell Biol 26:1077–1092 10.1038/s41556-024-01445-4 38926505

[CR231] Sun Z, Brodsky JL (2019) Protein quality control in the secretory pathway. J Cell Biol 218:3171–3187 10.1083/jcb.201906047 PMC6781448 31537714

[CR232] Suzuki K, Akioka M, Kondo-Kakuta C, Yamamoto H, Ohsumi Y (2013) Fine mapping of autophagy-related proteins during autophagosome formation in *Saccharomyces cerevisiae*. J Cell Sci 126:2534–2544 10.1242/jcs.122960 23549786

[CR233] Tan D, Cai Y, Wang J, Zhang J, Menon S, Chou HT, Ferro-Novick S, Reinisch KM, Walz T (2013) The EM structure of the TRAPPIII complex leads to the identification of a requirement for COPII vesicles on the macroautophagy pathway. Proc Natl Acad Sci USA 110:19432–19437 10.1073/pnas.1316356110 PMC3845172 24218626

[CR234] Tan JX, Lv B, Li J, Li T, Du F, Chen X, Zhang X, Bai XC, Chen ZJ (2026) PtdIns(3,5)P(2) is an endogenous ligand of STING in innate immune signalling. Nature 652:490–498 10.1038/s41586-025-10084-0 PMC13222494 41639454

[CR235] Tanigawa G, Orci L, Amherdt M, Ravazzola M, Helms JB, Rothman JE (1993) Hydrolysis of bound GTP by ARF protein triggers uncoating of Golgi-derived COP-coated vesicles. J Cell Biol 123:1365–1371 10.1083/jcb.123.6.1365 PMC2290881 8253837

[CR236] Theiler R, Fujita M, Nagae M, Yamaguchi Y, Maeda Y, Kinoshita T (2014) The alpha-helical region in p24gamma2 subunit of p24 protein cargo receptor is pivotal for the recognition and transport of glycosylphosphatidylinositol-anchored proteins. J Biol Chem 289:16835–16843 10.1074/jbc.M114.568311 PMC4059126 24778190

[CR237] Tisdale EJ, Jackson MR (1998) Rab2 protein enhances coatomer recruitment to pre-Golgi intermediates. J Biol Chem 273:17269–17277 10.1074/jbc.273.27.17269 9642298

[CR238] Tisdale EJ, Plutner H, Matteson J, Balch WE (1997) p53/58 binds COPI and is required for selective transport through the early secretory pathway. J Cell Biol 137:581–593 10.1083/jcb.137.3.581 PMC2139878 9151666

[CR239] Tojima T, Suda Y, Jin N, Kurokawa K, Nakano A (2024) Spatiotemporal dissection of the Golgi apparatus and the ER-Golgi intermediate compartment in budding yeast. eLife 13:e92900 10.7554/eLife.92900 PMC10950332 38501165

[CR240] Tooze J, Tooze S, Warren G (1984) Replication of coronavirus MHV-A59 in sac- cells: determination of the first site of budding of progeny virions. Eur J Cell Biol 33:281–293 6325194

[CR241] Tooze SA, Tooze J, Warren G (1988) Site of addition of N-acetyl-galactosamine to the E1 glycoprotein of mouse hepatitis virus-A59. J Cell Biol 106:1475–1487 10.1083/jcb.106.5.1475 PMC2115043 2836431

[CR242] van Vliet AR, Chiduza GN, Maslen SL, Pye VE, Joshi D, De Tito S, Jefferies HBJ, Christodoulou E, Roustan C, Punch E et al (2022) ATG9A and ATG2A form a heteromeric complex essential for autophagosome formation. Mol Cell 82:4324–4339 e4328 10.1016/j.molcel.2022.10.017 36347259

[CR243] Vavassori S, Cortini M, Masui S, Sannino S, Anelli T, Caserta IR, Fagioli C, Mossuto MF, Fornili A, van Anken E et al (2013) A pH-regulated quality control cycle for surveillance of secretory protein assembly. Mol Cell 50:783–792 10.1016/j.molcel.2013.04.016 PMC3699783 23685074

[CR244] Viotti C (2016) ER to Golgi-dependent protein secretion: the conventional pathway. Methods Mol Biol 1459:3–29 10.1007/978-1-4939-3804-9_1 27665548

[CR245] Volpicelli-Daley LA, Li Y, Zhang CJ, Kahn RA (2005) Isoform-selective effects of the depletion of ADP-ribosylation factors 1-5 on membrane traffic. Mol Biol Cell 16:4495–4508 10.1091/mbc.E04-12-1042 PMC1237059 16030262

[CR246] Wan W, Qian C, Wang Q, Li J, Zhang H, Wang L, Pu M, Huang Y, He Z, Zhou T et al (2023) STING directly recruits WIPI2 for autophagosome formation during STING-induced autophagy. EMBO J 42:e112387 10.15252/embj.2022112387 PMC10106988 36872914

[CR247] Wang H, Zhang M, Ge L (2022) Cholesterol: enhancing FGF2 translocation in unconventional secretion. J Cell Biol 221:e202210007 10.1083/jcb.202210007 PMC9582227 36255389

[CR248] Wang J, Davis S, Menon S, Zhang J, Ding J, Cervantes S, Miller E, Jiang Y, Ferro-Novick S (2015) Ypt1/Rab1 regulates Hrr25/CK1delta kinase activity in ER-Golgi traffic and macroautophagy. J Cell Biol 210:273–285 10.1083/jcb.201408075 PMC4508898 26195667

[CR249] Wang J, Menon S, Yamasaki A, Chou HT, Walz T, Jiang Y, Ferro-Novick S (2013) Ypt1 recruits the Atg1 kinase to the preautophagosomal structure. Proc Natl Acad Sci USA 110:9800–9805 10.1073/pnas.1302337110 PMC3683756 23716696

[CR250] Wang WA, Carreras-Sureda A, Demaurex N (2023) SARS-CoV-2 infection alkalinizes the ERGIC and lysosomes through the viroporin activity of the viral envelope protein. J Cell Sci 136:jcs260685 10.1242/jcs.260685 PMC10112968 36807531

[CR251] Wang Y, Huang M, Mu X, Song W, Guo Q, Zhang M, Liu Y, Chen YG, Ge L (2024) TMED10-mediated unconventional secretion of IL-33 regulates intestinal epithelium differentiation and homeostasis. Cell Res 34:258–261 10.1038/s41422-023-00891-3 PMC10907340 38185700

[CR252] Watanabe S, Kise Y, Yonezawa K, Inoue M, Shimizu N, Nureki O, Inaba K (2024) Structure of full-length ERGIC-53 in complex with MCFD2 for cargo transport. Nat Commun 15:2404 10.1038/s41467-024-46747-1 PMC10944485 38493152

[CR253] Watanabe Y, Allen JD, Wrapp D, McLellan JS, Crispin M (2020) Site-specific glycan analysis of the SARS-CoV-2 spike. Science 369:330–333 10.1126/science.abb9983 PMC7199903 32366695

[CR254] Waters MG, Serafini T, Rothman JE (1991) Coatomer’: a cytosolic protein complex containing subunits of non-clathrin-coated Golgi transport vesicles. Nature 349:248–251 10.1038/349248a0 1898986

[CR255] Weigel AV, Chang CL, Shtengel G, Xu CS, Hoffman DP, Freeman M, Iyer N, Aaron J, Khuon S, Bogovic J et al (2021) ER-to-Golgi protein delivery through an interwoven, tubular network extending from ER. Cell 184:2412–2429 e2416 10.1016/j.cell.2021.03.035 33852913

[CR256] Weisberg AS, Maruri-Avidal L, Bisht H, Hansen BT, Schwartz CL, Fischer ER, Meng X, Xiang Y, Moss B (2017) Enigmatic origin of the poxvirus membrane from the endoplasmic reticulum shown by 3D imaging of vaccinia virus assembly mutants. Proc Natl Acad Sci USA 114:E11001–E11009 10.1073/pnas.1716255114 PMC5754806 29203656

[CR257] Wendeler MW, Paccaud JP, Hauri HP (2007) Role of Sec24 isoforms in selective export of membrane proteins from the endoplasmic reticulum. EMBO Rep 8:258–264 10.1038/sj.embor.7400893 PMC1808030 17255961

[CR258] Westerbeck JW, Machamer CE (2019) The infectious bronchitis coronavirus envelope protein alters Golgi pH to protect the spike protein and promote the release of infectious virus. J Virol 93:10–1128 10.1128/JVI.00015-19 PMC6532078 30867314

[CR259] Wilson DW, Lewis MJ, Pelham HR (1993) pH-dependent binding of KDEL to its receptor in vitro. J Biol Chem 268:7465–7468 8385108

[CR260] Wissink EH, Kroese MV, van Wijk HA, Rijsewijk FA, Meulenberg JJ, Rottier PJ (2005) Envelope protein requirements for the assembly of infectious virions of porcine reproductive and respiratory syndrome virus. J Virol 79:12495–12506 10.1128/JVI.79.19.12495-12506.2005 PMC1211556 16160177

[CR261] Wong-Dilworth L, Rodilla-Ramirez C, Fox E, Restel SD, Stockhammer A, Adarska P, Bottanelli F (2023) STED imaging of endogenously tagged ARF GTPases reveals their distinct nanoscale localizations. J Cell Biol 222:e202205107 10.1083/jcb.202205107 PMC10140647 37102998

[CR262] Wu J, Sun L, Chen X, Du F, Shi H, Chen C, Chen ZJ (2013) Cyclic GMP-AMP is an endogenous second messenger in innate immune signaling by cytosolic DNA. Science 339:826–830 10.1126/science.1229963 PMC3855410 23258412

[CR263] Xiao L, Pi X, Goss AC, El-Baba T, Ehrmann JF, Grinkevich E, Bazua-Valenti S, Padovano V, Alper SL, Carey D et al (2024) Molecular basis of TMED9 oligomerization and entrapment of misfolded protein cargo in the early secretory pathway. Sci Adv 10:eadp2221 10.1126/sciadv.adp2221 PMC11414720 39303030

[CR264] Xu D, Hay JC (2004) Reconstitution of COPII vesicle fusion to generate a pre-Golgi intermediate compartment. J Cell Biol 167:997–1003 10.1083/jcb.200408135 PMC2172600 15611329

[CR265] Xu F, Du W, Zou Q, Wang Y, Zhang X, Xing X, Li Y, Zhang D, Wang H, Zhang W et al (2021) COPII mitigates ER stress by promoting formation of ER whorls. Cell Res 31:141–156 10.1038/s41422-020-00416-2 PMC8026990 32989223

[CR266] Xun J, Zhang Z, Lv B, Lu D, Yang H, Shang G, Tan JX (2024) A conserved ion channel function of STING mediates noncanonical autophagy and cell death. EMBO Rep 25:544–569 10.1038/s44319-023-00045-x PMC10897221 38177926

[CR267] Yamamoto K, Fujii R, Toyofuku Y, Saito T, Koseki H, Hsu VW, Aoe T (2001) The KDEL receptor mediates a retrieval mechanism that contributes to quality control at the endoplasmic reticulum. EMBO J 20:3082–3091 10.1093/emboj/20.12.3082 PMC150210 11406585

[CR268] Yan R, Chen K, Wang B, Xu K (2022) SURF4-induced tubular ERGIC selectively expedites ER-to-Golgi transport. Dev Cell 57:512–525 e518 10.1016/j.devcel.2021.12.018 PMC8891076 35051356

[CR269] Yang K, Feng Z, Pastor-Pareja JC (2024) p24-Tango1 interactions ensure ER-Golgi interface stability and efficient transport. J Cell Biol 223:e202309045 10.1083/jcb.202309045 PMC10932740 38470362

[CR270] Yang X, Lv L, Zhang Y, Zhang Z, Zeng S, Zhang X, Wang Q, Dorf M, Li S, Fu B (2025) ATP2A2 regulates STING1/MITA-driven signal transduction including selective autophagy. Autophagy 21:2230–2245 10.1080/15548627.2025.2496786 PMC12459357 40265346

[CR271] Yu X, Zhang L, Shen J, Zhai Y, Jiang Q, Yi M, Deng X, Ruan Z, Fang R, Chen Z et al (2021) The STING phase-separator suppresses innate immune signalling. Nat Cell Biol 23:330–340 10.1038/s41556-021-00659-0 33833429

[CR272] Yuan L, Kenny SJ, Hemmati J, Xu K, Schekman R (2018) TANGO1 and SEC12 are copackaged with procollagen I to facilitate the generation of large COPII carriers. Proc Natl Acad Sci USA 115:E12255–E12264 10.1073/pnas.1814810115 PMC6310809 30545919

[CR273] Zanetti G, Pahuja KB, Studer S, Shim S, Schekman R (2011) COPII and the regulation of protein sorting in mammals. Nat Cell Biol 14:20–28 10.1038/ncb2390 22193160

[CR274] Zavodszky E, Hegde RS (2019) Misfolded GPI-anchored proteins are escorted through the secretory pathway by ER-derived factors. eLife 8:e46740 10.7554/eLife.46740 PMC6541436 31094677

[CR275] Zeligs JD, Wollman SH (1979) Mitosis in rat thyroid epithelial cells in vivo. I. Ultrastructural changes in cytoplasmic organelles during the mitotic cycle. J Ultrastruct Res 66:53–77 10.1016/s0022-5320(79)80065-9 423323

[CR276] Zhang BC, Nandakumar R, Reinert LS, Huang J, Laustsen A, Gao ZL, Sun CL, Jensen SB, Troldborg A, Assil S et al (2020a) STEEP mediates STING ER exit and activation of signaling. Nat Immunol 21:868–879 10.1038/s41590-020-0730-5 PMC7610351 32690950

[CR277] Zhang C, Ma HM, Wu S, Shen JM, Zhang N, Xu YL, Li CX, He P, Ge MK, Chu XL et al (2024) Secreted PTEN binds PLXDC2 on macrophages to drive antitumor immunity and tumor suppression. Dev Cell 59:3072–3088 e3078 10.1016/j.devcel.2024.08.003 39197453

[CR278] Zhang C, Min YQ, Xue H, Zhang H, Liu K, Tian Y, Yang Z, Zhao Z, Yang H, Shan C et al (2025a) Host protein ARF1 is a proviral factor for SARS-CoV-2 and a candidate broad-spectrum therapeutic target. Nat Commun 16:6326 10.1038/s41467-025-61431-8 PMC12241596 40634337

[CR279] Zhang J, Kennedy A, de Melo Jorge DM, Xing L, Reid W, Bui S, Joppich J, Rose M, Ercan S, Tang Q et al (2025b) SARS-CoV-2 remodels the Golgi apparatus to facilitate viral assembly and secretion. PLoS Pathog 21:e1013295 10.1371/journal.ppat.1013295 PMC12208438 40540531

[CR280] Zhang JZ, Davletov BA, Sudhof TC, Anderson RG (1994) Synaptotagmin I is a high affinity receptor for clathrin AP-2: implications for membrane recycling. Cell 78:751–760 10.1016/s0092-8674(94)90442-1 8087843

[CR281] Zhang M, Kenny SJ, Ge L, Xu K, Schekman R (2015) Translocation of interleukin-1beta into a vesicle intermediate in autophagy-mediated secretion. eLife 4:e11205 10.7554/eLife.11205 PMC4728131 26523392

[CR282] Zhang M, Liu L, Lin X, Wang Y, Li Y, Guo Q, Li S, Sun Y, Tao X, Zhang D et al (2020b) A translocation pathway for vesicle-mediated unconventional protein secretion. Cell 181:637–652 e615 10.1016/j.cell.2020.03.031 32272059

[CR283] Zhang M, Schekman R (2013) Cell biology. Unconventional secretion, unconventional solutions. Science 340:559–561 10.1126/science.1234740 23641104

[CR284] Zhang W, Ji C, Li X, He T, Jiang W, Liu Y, Wu M, Zhao Y, Chen X, Wang X et al (2025c) Autophagy-independent role of ATG9A vesicles as carriers for galectin-9 secretion. Nat Commun 16:4259 10.1038/s41467-025-59605-5 PMC12059159 40335523

[CR285] Zhao X, Claude A, Chun J, Shields DJ, Presley JF, Melancon P (2006) GBF1, a cis-Golgi and VTCs-localized ARF-GEF, is implicated in ER-to-Golgi protein traffic. J Cell Sci 119:3743–3753 10.1242/jcs.03173 16926190

[CR286] Zheng J, Ge L (2022) Diverse cellular strategies for the export of leaderless proteins. Natl Sci Open 1:20220018

[CR287] Zheng J, Wang H, Sun Y, Chang P, Deng X, Zhang L, Zhu L, Zhu K, Peng D, Deng H et al (2026a) TMEDs mediate versatile cargo transport in vesicle-dependent unconventional secretion. J Cell Biol 225:e202503075 10.1083/jcb.202503075 41364076

[CR288] Zheng J, Xu C, Wang Z, Yao T, Wang H, Lin X, Zhu K, Liu Y, Peng D, Chen F et al (2026b) A sphingomyelin-sensing pathway governing metabolic inflammation. LangTaoSha Preprint Server. 10.65215/LTSpreprints.2026.01.18.000088

[CR289] Zheng X, Fang D, Shan H, Xiao B, Wei D, Ouyang Y, Huo L, Zhang Z, Wu Y, Zhang R et al (2025) The assembly of RAB22A/TMEM33/RTN4 initiates a secretory ER-phagy pathway. Cell Discov 11:41 10.1038/s41421-025-00792-2 PMC12041605 40301304

[CR290] Zoppino FC, Militello RD, Slavin I, Alvarez C, Colombo MI (2010) Autophagosome formation depends on the small GTPase Rab1 and functional ER exit sites. Traffic 11:1246–1261 10.1111/j.1600-0854.2010.01086.x 20545908

[CR291] Zuber C, Fan JY, Guhl B, Parodi A, Fessler JH, Parker C, Roth J (2001) Immunolocalization of UDP-glucose:glycoprotein glucosyltransferase indicates involvement of pre-Golgi intermediates in protein quality control. Proc Natl Acad Sci USA 98:10710–10715 10.1073/pnas.191359198 PMC58531 11535823

