# Supplementary material for: The ER–Golgi intermediate compartment: a central hub integrating membrane trafficking and stress responses
Source: EMBO Rep. 2026 Aug 22;27(18):5187–213. doi: 10.1038/s44319-026-00908-z (PMC13601593; doi:10.1038/s44319-026-00908-z)
Supplement: Supplementary file 1 [file 44319_2026_908_MOESM1_ESM.pdf]

EMBO reports

The ER-Golgi Intermediate Compartment: A Central Hub Integrating Membrane Trafficking and Stress Responses

Yingying Guo, Jianfei Zheng, Lei Liu, Shulin Li, Min Zhang, and Liang Ge

Corresponding author(s): Liang Ge (liangge@mail.tsinghua.edu.cn) , Min Zhang (zhangmin143@mail.tsinghua.edu.cn)

Review Timeline:

Submission Date:	1st Feb 26
Editorial Decision:	2nd Mar 26
Revision Received:	11th Apr 26
Accepted:	6th Jul 26

Editor: Martina Rembold

Transaction Report:

(Note: With the exception of the correction of typographical or spelling errors that could be a source of ambiguity, letters and reports are not edited. Depending on transfer agreements, referee reports obtained elsewhere may or may not be included in this compilation. Referee reports are anonymous unless the Referee chooses to sign their reports.)

1st Editorial Decision 2nd Mar 2026

Dear Liang,

Thank you for the submission of your review to EMBO reports. We have now received the full set of referee reports that is pasted below.

As you will see, all three referees consider the review interesting, comprehensive and timely, and have overall rather minor criticisms and suggestions how to further improve the article. Referee #1 points out that there is some overlap with a recent review, which you might want to review, but some overlap is certainly unavoidable given the comprehensive nature of your review.

From the editorial side, we need a paragraph called "Box 1 - in need of answers" that briefly outlines the major questions that are still open in a given field in the form of a few bullet points. These questions can be accompanied by a brief explanation of what would be needed to address them and may provide helpful towards setting the stage for future experimentation in the field. We further need a Disclosure and competing interests statement.

Please submit along with your revised review a detailed point-by-point response to the referee suggestions to facilitate the evaulation of your review.

As for timing, if you could submit the revised article by mid-April, I could include it in our second June issue. If you anticipate a problem meeting this deadline, then please let me know and we can aim for another issue. You can submit your revised files by logging onto our online manuscript tracking system.

I hope that the referees' comments do not prove too problematic to address and I look forward to receiving your next version.

Kind regards,

Martina

Martina Rembold, PhD
Senior Editor
EMBO reports

==================

Referee #1:

The manuscript offers a very comprehensive review of both historical and more recent evidence of ERGIC membranes playing a plethora of roles in the secretory pathway under various conditions. At times, the review may be seen as being too verbose, going into extensive detail. Additionally, there is overlap between this review and another recent review from Aridor (Subcell Biochem. 2026;111:3-36). The figures presented are somewhat repetitive and it would be more helpful to include imaging data from cells (fluorescence and EM) to correlate the models with reality. Overall however, the review should make for good reference material for experts and non-experts alike.

Referee #2:

The manuscript by Guo and colleagues provides a comprehensive and timely review of ERGIC structure and function. The topic of the review should be of interest to the broad readership of EMBO Reports. I have only minor suggestions to further strengthen the review.

1. On page 4, line 110; suggest writing "Lippincott-Schwartz and colleagues identified a BFA insensitive cycling compartment that was positive for P53 and distinct from Golgi proteins."

2. On page 10, lines 302-306, these two sentences are virtually identical to the two sentences on page 5, lines 151-154. In the second instance on page 10, suggest revising to emphasize how this organization supports a tunnel model.

3. On page 12, line 348, suggest writing "its fission yeast orthologue Emp43"

4. On page 14, line 428, suggest rewriting this passage to include the references and structural studies [PMID: 30846601 and PMID: 38626766] on the bound and unbound states of KDEL receptor. The Newstead lab findings indicate conformational

change in the KDEL receptor and not oligomerization is important for COPI packaging.

5. On page 20, line 603, the interpretation is that COPII vesicles are a principal membrane source for phagophore formation. My understanding is that there is some debate on the primary source and that there may be multiple sources depending on cell type and energetic status. I suggest mentioning this with references, that in addition to COPII vesicles other membranes may contribute to phagophore expansion, for example work on the lipid transfer complex (ATG2A-ATG9A) from the Tooze lab [PMID: 36347259] and a review on this topic [PMID: 37385155].

Referee #3:

Guo and colleagues submitted a very interesting and well-written review on the multifaceted roles of the ERGIC. The authors address all the relevant aspects of this compartment, from its discovery to the most recent literature. I particularly appreciated the introductory section describing the early observations and experiments that led to the identification of the ERGIC, as well as the level of detail dedicated to this historical perspective. The review then provides the reader with comprehensive information on the ERGIC and its interactions with the ER and Golgi, covering trafficking, signaling, and emerging regulatory functions. Overall, the review is well written and of broad interest. A few minor improvements might be required to improve clarity:

Figures should be more thoroughly cited and referred to throughout the text. At present, cross-references to figures are limited and should be expanded to better guide the reader.

Line 863: Golgi fragmentation does not necessarily indicate an alternative route of viral egress. Several reports suggest that the lysosomal route, particularly for SARS-CoV-2, may not represent the primary pathway, and brefeldin A treatment strongly inhibits particle release (10.1371/journal.ppat.1013295). In addition, the sentence is currently unclear and should be rephrased for clarity. Finally several reports show assembly also at the Golgi, showing that both the ERGIC and the Golgi membranes contribute to SARS-CoV-2 assembly (10.1126/sciadv.abl4895; 10.1016/j.chom.2020.11.003)

Figure 4B is only partially informative. Given that the review discusses STING translocation and signaling at the ERGIC, including a panel illustrating these aspects would be more informative.

A few minor typos are present (e.g., in vitro and in vivo should be italicized). In line 114, "positive" appears to be misspelled and should be corrected.

Referee #1:

Comment: The manuscript offers a very comprehensive review of both historical and more recent evidence of ERGIC membranes playing a plethora of roles in the secretory pathway under various conditions. At times, the review may be seen as being too verbose, going into extensive detail. Additionally, there is overlap between this review and another recent review from Aridor (Subcell Biochem. 2026; 111:3-36). The figures presented are somewhat repetitive and it would be more helpful to include imaging data from cells (fluorescence and EM) to correlate the models with reality. Over all however, the review should make for good reference material for experts and non-experts alike.

Response: We appreciate the reviewer's positive evaluation of our manuscript and the constructive suggestions provided. In response to the reviewer's comments, we have incorporated the recently published review by Aridor (Subcell Biochem. 2026; 111:3–36), which is now cited in the revised manuscript (page 7, lines 212-214) and highlighted for clarity.

In addition, we have included representative imaging data to link the conceptual models with experimental observations. Fluorescence microscopy and electron microscopy images have been added to Figure 4A and Figure 4B, respectively. The Figure 4 legend has been revised accordingly (page 53, lines 1890-1911), and the relevant descriptions in the main text have been updated to reflect these additions (pages 25-26, lines 769-775).

Specifically, because ERGIC participates in multiple trafficking pathways and performs distinct functions in different cellular contexts, we present these mechanisms in separate figures to improve conceptual clarity and avoid potential confusion. We believe that this organization, together with the newly added imaging data in Figures 4A and 4B, helps readers more easily distinguish the specific functional roles of ERGIC discussed throughout the review.

In addition, to ensure that the review reflects the most up-to-date advances in the field, we have incorporated several recently published studies, including work published in 2026, that provide new mechanistic insights into ERGIC function and STING trafficking. These have been cited and integrated into the revised manuscript at the relevant sections

Referee #2:

The manuscript by Guo and colleagues provides a comprehensive and timely review of ERGIC structure and function. The topic of the review should be of interest to the broad readership of EMBO Reports. I have only minor suggestions to further strengthen the review.

1. On page 4, line 110; suggest writing "Lippincott-Schwartz and colleagues identified a BFA insensitive cycling compartment that was positive for P53 and distinct from Golgi proteins."

Response: We appreciate the reviewer's helpful suggestion. The sentence has been revised accordingly and now reads: "Lippincott-Schwartz and colleagues identified a BFA insensitive cycling compartment that was positive for P53 and distinct from Golgi proteins." (pages 4-5, line 122-124).

2. On page 10, lines 302-306, these two sentences are virtually identical to the two sentences on page 5, lines 151-154. In the second instance on page 10, suggest revising to emphasize how this

organization supports a tunnel model.

Response: We appreciate the reviewer's constructive suggestion. We have revised the sentences on page 11 (lines 333–339) to avoid repetition and to better emphasize how the spatial organization of COPII and COPI coats along tubular ERGIC carriers supports the proposed tunnel model. The revised text now reads: "In these tubular carriers, COPII components localize predominantly to the basal region at the ER exit site (ERES) interface, where they are thought to regulate cargo sorting and membrane remodeling. By contrast, COPI coats decorate more distal regions of the tubular ERGIC. This spatial segregation of COPII and COPI along the tubular carriers supports the proposed tunnel model, in which elongated ERGIC tubules provide a conduit for directional cargo movement toward the Golgi."

3. On page 12, line 348, suggest writing "its fission yeast orthologue Emp43"

Response: We appreciate the reviewer's helpful suggestion. The text has been revised accordingly and now reads "its fission yeast orthologue Emp43" (page 13, line 384).

4. On page 14, line 428, suggest rewriting this passage to include the references and structural studies [PMID: 30846601 and PMID: 38626766] on the bound and unbound states of KDEL receptor. The Newstead lab findings indicate conformational change in the KDEL receptor and not oligomerization is important for COPI packaging.

Response: We appreciate the reviewer's helpful suggestion and for pointing out these important structural studies. Following this advice, we have revised the relevant passage to incorporate these references and to clarify that pH-dependent conformational changes in the KDEL receptor, rather than oligomerization, are key for COPI recruitment and packaging. The revised text now reads: "Further structural studies have revealed the molecular basis of KDEL receptor activation. Crystal structures of the receptor in cargo-bound and unbound states demonstrate that KDEL binding in the acidic Golgi lumen induces conformational rearrangements that enable pH-dependent recognition and retrieval of ER-resident proteins." (page 16, lines 471-475)

5. On page 20, line 603, the interpretation is that COPII vesicles are a principal membrane source for phagophore formation. My understanding is that there is some debate on the primary source and that there may be multiple sources depending on cell type and energetic status. I suggest mentioning this with references, that in addition to COPII vesicles other membranes may contribute to phagophore expansion, for example work on the lipid transfer complex (ATG2A-ATG9A) from the Tooze lab [PMID: 36347259] and a review on this topic [PMID: 37385155].

Response: We appreciate the reviewer's insightful suggestion. We have revised the text to clarify that COPII vesicles are not the sole membrane source for phagophore expansion, and we incorporated the suggested references on lipid transfer mediated by the ATG2A–ATG9A complex. The revised text now reads: "This coordinated regulation ensures the accurate positioning of COPII vesicles at the PAS and facilitates their functional cooperation with Atg9-positive vesicles during early phagophore formation. Once seeding the membrane at the PAS, bulk lipid transport mediated by ATG2 to support autophagosome membrane growth." (page22, lines 665-666)

Referee #3:

1. Guo and colleagues submitted a very interesting and well-written review on the multifaceted roles of the ERGIC.

Figures should be more thoroughly cited and referred to throughout the text. At present, cross-references to figures are limited and should be expanded to better guide the reader.

Response: We appreciate the reviewer's helpful suggestion. We have revised the manuscript to improve cross-referencing between the text and figures. Additional figure citations have been incorporated throughout the manuscript to better guide the reader.

2. Line 863: Golgi fragmentation does not necessarily indicate an alternative route of viral egress. Several reports suggest that the lysosomal route, particularly for SARS-CoV-2, may not represent the primary pathway, and brefeldin A treatment strongly inhibits particle release (10.1371/journal.ppat.1013295). In addition, the sentence is currently unclear and should be rephrased for clarity. Finally several reports show assembly also at the Golgi, showing that both the ERGIC and the Golgi membranes contribute to SARS-CoV-2 assembly (10.1126/sciadv.abl4895; 10.1016/j.chom.2020.11.003)

Response: We appreciate the reviewer's helpful suggestions. We agree that Golgi fragmentation does not necessarily indicate the presence of an alternative viral egress route, and that non-canonical secretory pathways, such as the lysosomal route, may not represent the primary pathway for coronavirus release. Accordingly, the relevant sentence has been revised for clarity in the manuscript (page 31, lines 939–942).

In addition, following the reviewer's suggestion, we have incorporated the cited references (10.1371/journal.ppat.1013295; 10.1126/sciadv.abl4895; 10.1016/j.chom.2020.11.003) and expanded the discussion to clarify that SARS-CoV-2 assembly can occur at both ERGIC and Golgi membranes, forming an ERGIC–Golgi assembly continuum. We also added discussion of the inhibitory effect of brefeldin A on viral particle release and summarized alternative Golgi-independent secretion routes described in the literature (page 32, lines 959–971).

3. Figure 4B is only partially informative. Given that the review discusses STING translocation and signaling at the ERGIC, including a panel illustrating these aspects would be more informative.

Response: We appreciate the reviewer's constructive suggestion. To better illustrate the complex regulatory landscape of STING at the ERGIC, we have added a new schematic illustration (Figure 5) to the revised manuscript. The corresponding figure legend and description have also been included in the relevant section of the text (pages 53-54, line 1912-1922). Accordingly, the original Figures 5 and 6 have been renumbered as Figures 6 and 7 in the revised manuscript.

4. A few minor typos are present (e.g., in vitro and in vivo should be italicized). In line 114, "positive" appears to be misspelled and should be corrected.

Response: We greatly appreciate the reviewer's helpful suggestion. We have performed a thorough manual check and ensured that all instances of in vitro and in vivo are now correctly italicized. The spelling error for "positive" on line 126 (and throughout the manuscript) has been corrected.

Prof. Liang Ge
Tsinghua university
School of Life Sciences
China

Dear Liang,

Thank you very much for sending the revised manuscript text and figures. I have uploaded all of them to the system. II am pleased to inform you that your manuscript has been accepted for publication in EMBO reports. Your figures will be edited by the external team of graphics editors we work with. Once their drafts are ready, you will be contacted for your approval. Once this is done, your manuscript will be processed for publication by EMBO Press. It will be copy edited and you will receive page proofs prior to publication. Please note that you will be contacted by Springer Nature Author Services to complete licensing information.

Should you be planning a Press Release on your article, please notify the production team via this form in order to coordinate publication and release dates: https://forms.cloud.microsoft/e/eH4A8JVP6g

If you have any questions, please do not hesitate to contact the Editorial Office. Thank you for your contribution to EMBO Reports. It was a pleasure working with you on this article.

With best wishes,

Martina

Martina Rembold, PhD
Senior Editor
EMBO reports

>>> Please note that it is EMBO Reports policy for the transcript of the editorial process (containing referee reports and your response letter) to be published as an online supplement to each paper. If you do NOT want this, you will need to inform the Editorial Office via email immediately. More information is available here: https://link.springer.com/partners/embo-press/editorial-policies#Peer%20review
